# Abstracts of the 9th Tanzania Health Summit: Dar Es Salaam, Tanzania.

**DOI:** 10.1186/s12919-023-00273-y

**Published:** 2023-08-22

## A1. Call-back strategy an approach for scaling up Covid-19 vaccination among PLHIV in Geita region, afya jumuishi project

### Edson Magadula^1^, Shukrani Mbwaga^1^, Florian Mutasingwa^1^, Emelda Herman^1^

#### ^1^Management and Development for Health (MDH), Dar es salaam, Tanzania

##### **Correspondence:** Edson Magadula (emagadula@mdh.or.tz)

*BMC Proceedings 2023*, **17(13):** A1

**Background**

Covid-19 pandemic continues to rage and affect our country with notable 37,510 cases and 841 deaths in Tanzania. In December 2022, WHO authorized the use of vaccines under emergency control to prevent COVID-19 associated mortality and morbidity. By January 31st, 2022, 14,733 PLHIVs (25.2%) vaccinated out of 58,509 in Geita, the need for urgent intervention in accelerating the vaccination in this priority group to 90% by June 2022.

**Methodology**

In February 2022, MDH in collaboration with R/CHMT introduced call-back strategy in the region. This strategy involved HCWs and CBHs who were oriented. Generated lists of 27,060 PLHIVs on 3&6MMD and shared to 44 tier 1 and tier 2 facilities for pulling files and calling for invitation to attend at the facility for vaccination. Those who returned were refunded transport costs. List of unreachable clients was left to CBHs, peers and lay counselors for physical follow-up.

**Results**

By June 30th, a total of 22,686 PLHIVs (83% from the shared list) were called and fully vaccinated. This contributed to 42% of total vaccinations (53,286 PLHIVs) with an overall achievement of 91% of the target. Through call back strategy 8400 doses of Moderna Vaccines which were nearly to expire were rescued.

**Conclusion**

The approach helped in reaching unvaccinated PLHIV at higher risk of Covid19 burden who were on 3&6 MMD, involving refund of transport costs. Innovations within implementation are key in accelerating timely achievements in program indicators along with monitoring of consumables and preventing wastage of resources.

## A2. The role of local mentors in speeding up accreditation for medical laboratories: experience from Tanzania

### Violasia W. Mushi^1^, Ibrahim Ideva^1^, David Temba^1^, ReginaldJulius^2^, Nzovu Ulenga^1^, Alex Magesa^2^, Solomon Mwaigwisya^1^, Amani Shao^1^

#### ^1^Management and Development for Health, Dar es salaam, Tanzania; ^2^Tanzania Ministry of Health, Dodoma, Tanzania

##### **Correspondence:** Violasia W. Mushi (vmushi@mdh.or.tz)

*BMC Proceedings 2023,* **17(13):** A2

**Background**

Quality Management Systems (QMS) in medical laboratories is the key to patient safety. However, achieving the QMS standard needs political will, collaborative efforts and resources for capacity building and supportive supervision. We aim to share experiences from Tanzania since 2009 when the country began interventions for improving QMS in medical laboratories.

**Methodology**

A retrospective analysis of program data between 2010 and 2022. ASLM 3 or more stars was the criteria for enrolment into accreditation program. Enrolled laboratories receive mentorship, QMS trainings, and supportive supervisions. Between 2009 to 2018 international mentors conducted at least three rounds of mentorships per year. From 2018 the qualified local mentors conducted three rounds of two-weeks mentorship per year then two rounds of one-week mentorship by international mentors. Laboratory managers and quality officers were attached to accredited laboratories for practical, in-class trainings and supportive supervision by the Ministry of Health with other stakeholders.

**Results**

Between 2009 and 2018 when we had no local mentors in the accreditation processes, 12 out of 15 laboratories were accredited with ISO 15189 standards. Between 2018 and 2022 June, the local mentors were involved and among 85 laboratories enrolled for accreditation, 43 laboratories were accredited with ISO 15189 standards, 5 laboratories did not qualify, 12 pending for assessment while 20 were on mentorship program prior assessment.

**Conclusion**

The involvement of local mentors and collaborative efforts by various stakeholders resulted in a four-fold increase in the accreditation of health laboratories within a short period. We recommend capacity building to local mentors and the use of available accredited laboratories to facilitate the unaccredited laboratories to achieve ISO 15189 accreditation.

## A3. Strengthening oxygen utilization and respiratory care ecosystem in Tanzania

### Jackson Mugyabuso^1^, Alex Sanga^2^, John Gamaliel^2^, Amos Mugisha^1^

#### ^1^PATH Tanzania, Dar es salaam, Tanzania; ^2^Ministry of Health, Dodoma, Tanzania

##### **Correspondence:** Jackson Mugyabuso (jmugyabuso@path.org)

*BMC Proceedings 2023,* **17(13):** A3

**Background**

Strengthening oxygen availability in Tanzania needs stakeholders’ collaboration. Equipment monitoring is essential to ensure uninterrupted functionality and seamless availability of oxygen. For sustainability, we recommend incorporating budget for medical oxygen devices and maintenance in comprehensive council health plans (CCHPS).

**Methodology**

In 2020, during COVID 19 pandemic, MOH collaborated with PATH to strengthen availability of oxygen through participation in National oxygen technical working group, assessment of oxygen system for availability and functionality of equipment, and training needs on EECC and IPC. 251 health facilities from eight regions of Morogoro, Dodoma, Singida, Kagera, Mara, Njombe, Kigoma and Simiyu were assessed. Needs were quantified, procured, and trainings were conducted.

**Results**

As results for assessment, 3,809 respiratory devices (i.e., oxygen concentrators, suction machines, anesthesia machines and patient monitors) in supported regions were repaired.1,126 oxygen accessories for Dodoma & Kagera regions were procured. Some 877 clinicians, nurses from 117 health facilities were trained on EECC and IPC. Some 65 (biomedical engineers, ICT officers, and HCWs) trained on medical equipment and infrastructure management information system (MEIMIS). 8 laptops were procured to monitor equipment functional status, and 6 biomedical repair kits were provided.

**Conclusion**

Tanzania like other Low- and Middle-Income Countries (LMICs) experienced challenges in medical oxygen demand during Covid-19 pandemic. Most health facilities lacked functional biomedical equipment and human resource capacity to provide essential emergency & critical care (EECC).

PATH through UNITAID and Bill and Melinda Gates foundation support, collaborated with Ministry of Health (MOH) and Presidents’ Office of Regional Administration and Local Government (PORALG) to coordinate respiratory care response. We are reporting strengthening of respiratory care system in Tanzania.

## A4. Assessing laboratory capacity for anti-microbial resistance detection in sentinel sites in Tanzania

### Siril Kullaya^1^ Bachana Rubegwa^1^, John Gamaliel^1^, Amos Mugisha^1^, Lindsey Shields^2^, Lusubilo Malakbungu^3^, Carrie Reed^4^

#### ^1^PATH Tanzania, Dar es salaam, Tanzania; ^2^PATH Seattle US, Washington, United States of America; ^3^FHI360 Tanzania, Dar es salaam, Tanzania; ^4^USAID Tanzania, Dar es salaam, Tanzania

##### **Correspondence:** Siril Kullaya (skullaya@path.org)

*BMC Proceedings 2023,* **17(13):** A4

**Background**

Antimicrobial resistance (AMR) is one of the greatest threats to public health of our time. Countries are working to strengthen AMR detection and response by operationalizing national action plans, strengthening sentinel surveillance sites, and reporting antimicrobial susceptibility testing (AST) results to national and global systems.

**Methodology**

The assessment was conducted in 2019-2020 in 4 AMR sentinel sites of Tanzania in Kigoma, Morogoro, Dodoma and Dar es Salaam regions using CDC Laboratory Assessment of Antibiotic Resistance Testing Capacity (LAARC) tool. Site-specific quantitative scores were generated on 15 technical areas. Each facility received a percentage score between 0 and 100 in each of the technical areas. Higher scores indicated better performance in that technical area.

**Results**

Kigoma scored the highest (72%), with gaps in general laboratory facility, laboratory information system, and quality control mechanisms for the identification of bacterial isolates. Morogoro and Temeke laboratories scored equally overall (68%) with critical gaps in the quality control procedures for AST testing. Dodoma scored lowest (64%) of the four IDDS supported sites with low scores in the laboratory information system and quality control procedures for AST.

**Conclusion**

Our results highlight the need for building diagnostic capacity for AMR detection and reporting to combat infectious disease threats and contribute to improvement in global AMR surveillance.

## A5. Neglected detectives: Engaging community health workers (CHW) to increase TB case detection in Kagera region

### Elieza Milonga^1^

#### ^1^Management and Development for Health, Dar es salaam, Tanzania

##### **Correspondence:** Elieza Milonga (emilonga@mdh.or.tz)

*BMC Proceedings 2023,* **17(13):** A5

**Introduction**

Tanzania is among the 30 high burden countries in the world, with almost 41% of the people estimated to have had TB are missed by the health system posing a greater risk of spreading the infection to family, friends, and other community members This has been a result of several key challenges including suboptimal screening practices at facilities and poor health seeking behaviors among the symptomatic and asymptomatic people in the community, CHWs were empowered and fully engaged in TB case detection.

**Methodology**

MDH created a new shape of community structure by placing 216 CHWs (Neglected detectives) at the Centre of ADDO, Traditional Healers, and Bodaboda riders strongly to generate demand for and improve the quality of TB prevention, treatment, and care services across all 323 health facilities through contact tracing, sample collection, and support sample referrals.

**Results**

CHWs have contributed on average 48% of all notified TB cases since 2019 2021 while pushing up the average change rates of total notification from 10.3% to 21% before and after the engagement of CHWs( 2014|7, 2018 |21) therefore, empowering and fully engaging CHWs is an effective way of increasing TB case detection.

**Implication**

Despite all the efforts made so far, Catastrophic cost reduces income in both individuals by 50% and households by 33% leading to delayed care seeking when TB symptoms set in, and Poor adherence to anti TB regimens leading to an increased threat of drug resistant TB.

## A6. Clinical characteristics, determinants, and outcome of sars-cov-2 infected patients with renal dysfunction attending Kilimanjaro Christian Medical Centre

### Sweetness N. Laizer^1^, Modesta Mitao^2^, Kajiru Kilonzo^1^, Gilbert Waria^1^, Nyasatu Chamba^1^,Mercy Naftal^2^, Sweetness Laizer^1^

#### ^1^Kilimanjaro Christian Medical University College, Moshi, Tanzania; ^2^Kilimanjaro Clinical Research Institute, Moshi, Tanzania

##### **Correspondence:** Sweetness N. Laizer (s.laizer@kcri.ac.tz)

*BMC Proceedings 2023,* **17(13):** A6

**Introduction**

The SARS-CoV-2 pandemic has resulted in a continuous rise in morbidity and mortality globally. Renal dysfunction is one of the most common manifestations of SARS-CoV-2 that increases mortality among the affected. However, there is a paucity of information on the determinants and outcomes of renal dysfunction among SARS-CoV-2 patients.Objective: To determine the clinical characteristics, determinants, and outcome of hospital mortality of SARS-CoV-2 infected patients with renal dysfunction attending KCMC.

**Methodology**

This was a hospital-based cross-sectional study of patients confirmed with SARS-CoV-2 admitted to the isolation ward at KCMC hospital where data was obtained from the SARS-CoV-2 isolation ward excel spreadsheet database collected during the four waves from 20th March 2020 - 26th Feb 2022. The study included 570 participants with confirmed SARS-CoV-2 admitted to the Isolation ward. Patients with end-stage renal disease and those with missing serum creatinine values were excluded.

**Results**

This study included 491 patients with confirmed SARS-CoV-2 with a mean age of 62.9, 57.1% were male, and hypertension and diabetes mellitus were the most common comorbidities at 49.7% and 30.8%, respectively. 43.2% of patients with SARS-CoV-2 had renal dysfunction. 67.9% and 48.7% of these patients had confusion and presented with severe SARS-CoV-2 respectively. Those aged 60 years and above, males, history of diabetes mellitus, and presence of severe SARS-CoV-2 were significantly associated with higher odds of renal dysfunction among patients with SARS-CoV-2. 54% of SARS-CoV-2 patients with renal dysfunction died.

**Conclusion**

There is increased mortality among SARS-CoV-2 patients with renal dysfunction. Age, male sex, and the presence of diabetes were significant determinants of renal dysfunction among patients with SARS-CoV-2. Further efforts are needed in the early stratification of these patients to aid prompt management.

## A7. The use of front desk nurse to outsource stable clients and improve 6 multi-months dispensing coverage in Simiyu region

### Sarah Kweyamba^1^, Kidenya Sitta^1^, Mathias Abuya^1^, Victor Mponzi^1^, Rita Mutayoba^1^, Edwin Kilimba^1^, Simon Mkawe^1^, Eric Mboggo^1^, Khamis Kulemba^2^

#### ^1^Amref Health Africa, Dar es salaam, Tanzania; ^2^Regional AIDS Control Coordinator (RACC), Dar es salaam, Tanzania

##### **Correspondence:** Sarah Kweyamba (Sarah.Kweyamba@amref.org)

*BMC Proceedings 2023,* **17(13):** A7

**Introduction**

Tanzania adopted the “Test and Start all” strategy aiming at individual testing for HIV and same day ART initiation to all HIV positives1. In August 2021 MOHCDGEC through NACP released a circular for initiating 6MMD to all eligible clients. In Simiyu region implementation started early September 2021. This abstract seeks to unleash the efficacy of front desk approach (FDA) to increase 6MMD coverage among eligible clients and the ultimate goal is to reduce work load to health workers, and increase client satisfaction.

**Methodology**

Learning from other supported health facilities in Tanga, training was conducted to Care and Treatment Clinics personnel to sort and outsource eligible clients. Eligibility criteria included clients on ART >6months, Age ≥5 years, no adverse drug reactions that require regular monitoring, no current Opportunistic Infections and uncontrolled co-morbidities, have good ART adherence of 95%, Undetectable HVL 350 cells/mm3 and being on first- or second-line ART. Additionally, clients who have been on 3 MMD for more than 2 visits were identified through file color coding.

**Results**

With the intervention implemented from September 2021 until February 2022, we managed to scale up 6MMD from 27% in September 2021 (Eligible for 6MMD=17,400; given 6MMD=4,725) to 95% by end of February 2022 (Eligible for 6MMD=23,870; Given 6MMD=22,706). Utilization of FDA has reduced time spent at the clinic from 2 hours to less than 45 minutes, put more clients on 6MMD reduce cost to clients spent on fare and ultimately more satisfaction with services.

**Conclusion**

The FDA is an effective and great approach for rapid scale up 6MMM and ultimately reduce work load to HCWs and spare time for support clients in need of attention. The FDA is an efficient approach which ensures timely initiation of 6MMD/refill, client satisfaction and is highly recommended.

## A8. Impacts of improved cryptococcal disease screening in adults with advanced HIV disease in Maswa district hospital

### Kidenya Sita^1^, Victor Mponzi^1^, Rita Mutayoba^1^, Mathias Abuya^1^, Khamis Kulemba^2^, Simon Mkawe^1^, Erick Mbogo^1^, Edwin Kilimba^1^, Sarah Kweyamba^1^

#### ^1^Amref health Africa, Dar es salaam, Tanzania; ^2^RAS SIMIYU, Simiyu, Tanzania

##### **Correspondence:** Kidenya Sita (Kidenya.Sita@Amref.org)

*BMC Proceedings 2023,* **17(13):** A8

**Introduction**

Despite of introduction of Test and treat since 2016, still half of the people living with HIV present to care with Advanced HIV Disease (AHD) and many people continue to die from HIV-related opportunistic infections. WHO and Tanzania guideline recommends AHD screening to all ART-naive adults and adolescents with CD4 cell count of <200 cells/ μL or WHO stage 3 and 4 if CD4 testing is not available. Clients with AHD need to be screened and treated for cryptococcal disease. Starting ART in clients with untreated asymptomatic antigenemia can be a fatal iatrogenic error.

**Methodology**

Amref Tanzania through CDC financial support in collaboration with R/CHMT, MOH through NACP conducted AHD training, support availability of CD4 catridge and CrAg test kit, technical assistance, mentorship and support data capturing tools as well fluconazole for preemptive therapy.

**Results**

In period of two years, we managed to improve the testing rate for CD4 and CrAg test from 23% in July 2020 to 99% in Jun 2022. Out of 109 ART naïve adult with CD4 count <200 cells/μL, 9 (1.6%) were CrAg positive asymptomatic and all were kept on preemptive therapy. The prevalence of CD4<200 cells/ μL ranged from 20% to 38% and prevalence of CrAg positive among clients with CD4<200 cells/ μL ranged from 13% to 33%.

**Implications**

Quarter to almost half of ART naïve clients present with CD4 <200 cells/ml with CrAg positivity rate as high as 13% to 33%. Screening and treatment help to reduce risk of death from immune reconstitution inflammatory syndrome and full blown Cryptococcal meningitis among clients initiated on ART. We recommend strengthening baseline CD4 testing and scale up cryptococcal disease screening and treatment.

## A9. Assessing implementation processes influencing maternal death surveillance and review among members of maternal death review committees in Dodoma city Tanzania

### Nelson Rumbeli^1^, Furaha August^4^, Nathanael Sirili^2^, Valeria Syrilivestry^3^

#### ^1^Department of Epidemiology and Biostatistics Muhimbili, University of Health and Allied Sciences, Dar es Salaam, Tanzania; ^2^Department of development studies, Muhimbili University of Health and Allied Sciences, Dar es salaam, Tanzania; ^3^Parasitology department, Muhimbili University of Health and Allied Sciences, Dar es salaam, Tanzania; ^4^ Department of gynecology and Obstetrics, Muhimbili University of Health and Allied Sciences, Dar es salaam, Tanzania

##### **Correspondence:** Nelson Rumbeli (nelsonrumbeli8@gmail.com)

*BMC Proceedings 2023,* **17(13):** A9

**Introduction**

About 295 000 deaths were reported following pregnancy complications in 2017, maternal mortality is still a problem. Of these deaths, 94%occurred in LMIC. This challenge is very high in Tanzania with MMR of 556/100,000 per live birth despite different interventions including maternal death review to solve the challenge of maternal. In 2013 (WHO) introduced (MDSR) adopted in Tanzania in 2015. However, the strategy faces some challenges in implementation.

**Methodology**

A qualitative case study was conducted, and conceptualized according to Consolidated implementation framework (CFIR) focusing on implementation process domain, Purposeful sampling was used to recruit 15 members of MDSR Committees. In- depth interviews were conducted with key informants concerning the implementation processes influencing MDSR committees to implement the strategy. Qualitative inductive content analysis was used to analyse data.

**Results,**

We observed that implementation processes that influenced MDSR committee are the inclusiveness in participatory planning process, stakeholder’s readiness and accountability and Collective learning in implementing MDSR strategy.

**Implication**

Focusing on the domain of implementation processes, our study observed that the inclusiveness in participatory planning process, stakeholder’s readiness and accountability and Collective learning can positively influence the implementation of MDSR strategy. Further studies are needed to analysis which and how other domain in consolidated implementation framework affect MDSR implementation.

## A10. Outreach service-an effective approach to reducing teenage pregnancy in katavi region-experience from Amref Health Africa in Tanzania

### Bahati M. Shaame^1^, Rose Olotu^1^, Serafina Mkuwa^1^, Godwin Munuo^1^, Michael Machaku^1^, Aisa Muya^1^, Frida Ngalesoni^1^, Elida Machungwa^2^, Paschal Wilbroad^3^

#### ^1^Amref Health Africa in Tanzania, Dar es salaam, Tanzania; ^2^Regional Reproductive and Child Health Coordinator-Katavi, Katavi, Tanzania; ^3^USAID Tanzania, Dar es salaam, Tanzania

##### **Correspondence:** Bahati M. Shaame (Bahati.Shaame@amref.org)

*BMC Proceedings 2023,* **17(13):** A10

**Background**

Globally, about one-tenth of the pregnancies that occur every year are among teenagers, in Sub-Saharan Africa (WHO). Pregnant teenagers are at increased risk for school dropout, low birth weight delivery, and maternal mortality. In Tanzania, about a quarter (23%) of girls aged between 15 and 19 have either given birth or become pregnant. Katavi is the leading region in Tanzania with a 45% teenage pregnancy and 14% of maternal mortality rate.

Despite the efforts, put in place to combat teenage pregnancies still, the problem exists. To complement the innovations, the project in collaboration with the Reproductive and Child Health Family Planning Unit is implementing community-based family planning (FP) services through outreach services has increasing demand among FP beneficiaries including adolescents and youth in the Katavi region.

**Methodology**

Using trained Community Health workers (CHWs) in collaboration with community leaders FP and SRH demand creation activities are conducted at the community level through different approaches e.g. music drama and magnetic theater. SRH including FP services is then provided in these gatherings. Through SRHR clubs and loyalty cards introduced to secondary schools, teenagers are accessing youth-friendly services at nearby health facilities in a friendlier environment.

**Results**

From 2021 to 2022 June a total of 43636 individuals (M19369 F 24267) were reached with SRH and FP education, whereby 40170 (92%) (M 19002 F 21168) were Youth aged 10-19, 4959 (M 1144 F 3815) were Referred for SRH and FP services and 4956 (99.9%) (M 1964 F 2992) got services of their choice. (Source: DHIS2-2019-2021).

**Conclusion**

Community-led solution using trusted Community structure to tackle Community myths around Family Planning services, strengthening outreach services using CHWs to create demands and HCWs to provide proper counseling for methods Mix and Informed Choices, will facilitate the utilization of SRH and FP services and decreaseteenagee pregnancy.

## A11. Mentor-mothers and PMTCT local mentors, game-changers in improving early infant diagnosis coverage in southern highland regions

### Eliufoo S. Churi^1^, Amos Nsheha^3^, Sally Talike Chalamila^3^, Janeth Mwambona^2^, Rosemary Mrina^4^, Mercy Cyprian Mahenge^3^, Patricia Agaba^4^, Shaban Ndama^4^, David Maganga^1^

#### ^1^HJF Medical Research International (HJFMRI), Mbeya, United Republic of Tanzania; ^2^ Walter Reed Army Institute of Research (WRAIR) | DOD, Robert Grant Ave, United States of America; ^3^ HJF Medical Research International (HJFMRI), Mbeya, United Republic of Tanzania; ^4^ Department of International HIV Prevention and Treatment (IHPT), U.S. Military HIV Research Program (MHRP), Dar es salaam, Tanzania

##### **Correspondence:** Eliufoo S. Churi (echuri@wrp.or.tz)

*BMC Proceedings 2023,* **17(13):** A11

**Introduction**

Henry Jackson Foundation Medical Research International-Tanzania (HJFMRI-T) is complementing the government of Tanzania’s efforts in providing Prevention of Mother To Child Transmission and Early Infant Diagnosis services in Mbeya, Rukwa, Songwe, and Katavi regions. According to the UNAIDS progress report, the latest national coverage of Early Infant Diagnosis (EID) among infants below 2 months is significantly low (46.6%). By October 2021, EID below 2 months EID coverage was 62%.

**Methodology**

HJFMRI-T deployed trained 371 mentor-mothers at 97 PEPFAR-supported facilities. Mentor-mothers are stable peer-led women attached to mother-infant pairs for peer-based psychosocial counseling, conducting mobile appointment reminders, physical visits, and providing health education. Mobile phones, airtime, backpacks, rainboots, and umbrellas were provided to ensure conducive working atmosphere. Meetings to sharpen mentor-mothers’ technical skills and deployed trained PMTCT mentors were conducted to ensure EID demand creation, timely tracking and EID sample collection and packaging was done with quality.

**Results**

After one round of mentor mother meeting mentorship sessions, below 2 moths EID coverage improved from 69% in March 2021 to 74% in September 2021. In a space of one year, a tremendous improvement in the coverage of EID below 2 months, from 62% in October 2020 to 93% in December 2022 was noticed.

**Implication**

Deployment of Mentor-mothers and PMTCT mentors had a significant impact on the improvement of EID coverage and continuity of treatment for Mother-Infant Pairs. Capacity building through onsite coaching and mentorship is vital to ensuring demand creation, EID sample quality, and documentation.

## A12. Reducing neonatal mortality in Malinyi district, Tanzania

### Benatus Sambili^1^, Gissela Makwisa^1^, Peter Hellmold^1^, Federica Laurenti^1^, Michael Hobbins^1^, Karolin Pfeiffer^1^,

#### ^1^SolidarMed, Ifakara, Tanzania

##### **Correspondence:** Benatus Sambili (b.sambili@solidarmed.ch)

*BMC Proceedings 2023,* **17(13):** A12

**Introduction**

In the rural district of Malinyi, a major reason for the high neonatal mortality of 35 per 1000 live births is the lack of adequate newborn care. Kangaroo Mother Care (KMC), the recommended method of care for preterm infants, became a national policy in 2008 in Tanzania. Still, until 2019, none of the seven rural district hospitals in the Morogoro region had neonatal care unit. To overcome this challenge, SolidarMed established a service delivery model enhanced by state-of-the-art equipment at a rural hospital in Malinyi.

**Methodology**

Between 2019 and 2021, SolidarMed in partnership with the district authorities, established a functional Neonatal Care Unit (NCU), Isolation room and KMC Unit at Lugala Hospital. Neonatal care quality was optimized through new infrastructure and equipment, including 2 Continuous Positive Airway Pressure machines, trainings, supportive supervision and ongoing coaching and mentorship from experienced paediatricians to 46 nurses and clinicians. Community awareness about KMC and mother-newborn-child health was raised in all 33 villages of the district through trainings to HCWs and sensitization events by CHWs. Dedicated sensitization material was produced in collaboration with the Ministry of Health.

**Results**

From June 2019 – 2021, a total of 1,099 newborns (47.8% premature babies) were admitted to the neonatal care unit at the hospital, with a survival rate increased from 75.9% (2018, before the start of the project)) to 91.4% (2021). Causes of death are: 63% prematurity, 22% asphyxia, 8% infection, 7% other.

**Implication**

Project results aroused interest among health authorities at the regional level, who expressed interest in scaling up the project in the regional hospital and additional districts. In 2022 SolidarMed started the implementation of this new project in 3 hospitals. An additional research component has been included to compare the costs and effectiveness of interventions for further scale-up in Tanzania and to investigate short-term maintenance of impact by follow-up of newborns after discharge.

## A13. Harnessing longitudinal data to improve adolescent health in Tanzania

### Innocent Yusufu^1^, Mary Mwanyika Sando^1^, Wafaie Fawzi^2^, David Sando^3^, Abbas Ismail^4^, Mashavu Yussuf^1^, Amani Tinkasimile^1^, Frank Mapendo^1^, Agness Mchome^5^

#### ^1^Africa Academy for Public Health, Dar es salaam, Tanzania; ^2^Havard T.H.Chan School of Public Health, Massachusetts, United States of America; ^3^Management and Development for Health, Dar es salaam, Tanzania; ^4^University of Dodoma, Dodoma, Tanzania; ^5^Tanga City Council, Tanga, Tanzania

##### **Correspondence:** Innocent Yusufu (iyusufu@aaph.or.tz)

*BMC Proceedings 2023,* **17(13):** A13

**Background**

25% of Tanga population are adolescents, yet the region lacks information in areas of adolescent health. Adolescents have been overlooked leaving an absence of information on risk factors and fewer health gains in this group compared to others. The objective was to use standardized survey among adolescents to collect key adolescent health indicators related to nutrition, sexual and reproductive health, mental health, physical health, health services utilization and substance use.

**Methodology**

This cross-sectional baseline survey in Tanga employed a mixed method approach. A total of 1,031 in-school adolescents (10-19 years) from 20 schools, 20 school administrators, and 231 out-of-school adolescents from 8 hotspots participated. Descriptive analysis was conducted and results presented in proportions and means with 95% confidence intervals. Subgroup analysis to assess variations in various dimensions of adolescent’s health by gender and age was done.

**Results**

Findings showed that majority (69%) of all surveyed adolescents did not attain dietary diversity. Approximately 18% of the interviewed adolescents were stunted. 6% were underweight, 8% were overweight. Moreover, 36% of the interviewed adolescents were found to be anemic. About 45% of the adolescents preferred to receive sexual and reproductive health information from teachers, and 67% wished to have more classes on the topic. 7% of the interviewed adolescents reported to have ever tried smoking. Overall, 37% of adolescents reported to be physically active for at least 1 hour for 6 to 7 days of the week.

**Conclusion**

Survey results highlights key adolescent health and nutrition intervention areas for Tanga City. With these findings we recommend revamping of ongoing adolescent interventions per the national guidelines and local policies, gleaning from the health seeking behavior and preferred access points for Tanga in-school and out-of-school adolescents.

## A14. Understanding the determinants of effectiveness of the enrolment, and sales forces for the CHF iliyoboreshwa, preliminary analysis of a mixed methods multiple case study in Tanzania

### Albino Kalolo^1^, Katrin Mwimbe^2^, Sia Tesha^3^, Elizeus Rwezaula^3^, Manfred Stoermer^5^, Ally Kebby Abdallah^3^, Ntuli A Kapologwe^4^

#### ^1^St Francis University College of Health and Allied Sciences (SFUCHAS), Ifakara, Tanzania; ^2^Domiya Estate Ltd, Dodoma, Tanzania; ^3^Health Promotion and Systems strengthening (HPSS) project, Dodoma, Tanzania; ^4^Presidents Office-Regional Administration and Local Government (PORALG), Dodoma, Tanzania; ^5^Swiss Tropical and Public Health Institute, Basel, Switzerland

##### **Correspondence:** Albino Kalolo (kaloloa@gmail.com)

*BMC Proceedings 2023,* **17(13):** A14

**Introduction**

Community based insurance schemes have potentials to advance progress to universal health coverage in the settings they are implemented. The persistent problem of low enrolment could be resolved if there are effective mechanisms for enrolment processes, sales forces, scheme branding and the involvement of the private sector. This study assessed the effectiveness of the enrolment and reenrolment processes, branding and marketing for the CHF iliyoboreshwa (iCHF), their determinants in six regions of Tanzania.

**Methodology**

This study employed a mixed methods multiple case study design. The mixed methods entail a concurrent collection and analysis of both quantitative and qualitative data .The multiple case study approach entails collection of data in multiple cases determined by performance in enrolment, branding and marketing in the stride forward to understand the heterogeneity and their determinants. We utilized multiple data collection tools (survey questionnaire, focus group discussion (FGDs), in-depth interviews and a document review checklist).Quantitative data were analysed using inferential statistics and a thematic analysis was employed for qualitative data.

**Results**

We found a presence of functional enrolment and sales mechanisms with suboptimal effectiveness. The enrolment mechanisms were challenged by IMIS technicalities, stock outs of enrolment materials, enrolment workforce challenges (number, performance, motivation, and payment modalities) and limited enrolment options. Predominance of traditional marketing mechanisms (government structure meetings and periodic social marketing). The contextual influencers included: inadequate health insurance knowledge, lack of governance and legal framework for iCHF, dwindling political will, unsatisfied service providers and unresponsive community.

**Conclusion**

The enrolment, marketing and branding of the CHF iliyoboreshwa is influenced by contextual factors that should be addressed as an important effort to improve visibility and uptake of the scheme. Interventions that target enrolment, marketing and branding of the scheme are highly needed in order make the scheme viable and sustainable.

## A15. The prevalence and factors associated with premature birth among post-delivery women in Mbeya region

### Glory Godfrey Mawolle^1^, Fabiola Moshi^1^

#### ^1^The University of Dodoma, Dodoma, Tanzania

##### **Correspondence:** Glory Godfrey Mawolle (glory.mawolle@yahoo.com)

*BMC Proceedings 2023,* **17(13):** A15

**Background**

Prematurity accounts for approximately 75% of neonatal mortality and is the primary cause of neonatal fatalities worldwide. Mbeya region where this study is focused on identifying the prevalence and associated factors of preterm birth has a 31% of neonatal deaths caused by prematurity. The main goal of this study was to assess the prevalence and factors associated with premature birth among post-delivery women in Mbeya Region.

**Methodology**

A hospital-based analytical cross-sectional study was conducted among 233 post-delivery women and their neonates from April to May 2022 in Mbeya region. A census method was used to select all district and referral health facilities and a simple random sampling with lottery replacement technique was used to select study participants. A structured questionnaire was used for data collection. Frequencies, percentages, chi-square analysis, and logistic regression were used in the data analysis using SPSS version 25 to compare and explain the prevalence and contributing variables of preterm delivery. A variable in the final model was judged statistically significant when it had a 95% Confidence Interval (CI) and a p-value of 0.05.

**Results**

The mean maternal age (SD) was 26 ± 6. The prevalence of preterm birth in Mbeya Region was found to be 142 (39.1%). Child space <24months (AOR=3.058; p-value 0.045), not use malarial prophylaxis (AOR=5.418; p-value 0.008), twin pregnancy (AOR=4.657; p-value < 0.001), violence during pregnancy (AOR=2.059; p-value 0.048), lack of social support (AOR=1.993; p-value 0.022) and use of pica during pregnancy (AOR=1.880; p-value 0.029) found to be significant associated with premature birth.

**Conclusion**

In conclusion, not using malarial prophylaxis, child space <24 months, twin pregnancy, use of pica, violence, and lack of social support are shown to be major factors associated with preterm birth. Hence, midwives should emphasize pregnant women on the use of all recommended prophylaxis as recommended by the Ministry of Health under focused antenatal care.

## A16. Parents/caregivers’ food handling practices and aflatoxin dietary exposure among children aged 6 - 23 months in Bukombe district, Geita

### Mariam Said Nakuwa^1^, Richard Mongi^1^, Selestin Ngoma^1^

#### ^1^University of Dodoma, Dodoma, Tanzania

##### **Correspondence:** Mariam Said Nakuwa (lisanakuwa@gmail.com)

*BMC Proceedings 2023,* **17(13):** A16

**Background**

Introduction of safe complementary food to young children is essential for health and well-being of children. Cereals are the main component of complementary food in Tanzania, these foods are prone to aflatoxin contamination. Higher levels of aflatoxin contaminated maize and groundnuts from Bukombe District have been reported. Therefore, the current study aimed to determine prevalence and factors associated with aflatoxin dietary exposure among children aged 6-23 months in Bukombe District.

**Methodology**

An analytical cross-sectional study was used to assess 342 randomly selected parents/caregivers from households with children aged between 6 - 23 months. Samples of complementary flour were collected from willingly 50 households for Aflatoxin quantification by using HPLC and estimates of dietary exposure. Descriptive and inferential statistics were analyzed with SPSS version 25 where frequency, percentage, Chi square and p-values were presented.

**Results**

Most of parents choose maize as the main component of complementary flour, either on its own or in composite. Winnowing is the most processing practice while dehulling is the least, and milled flour stored for more than two weeks. Majority of flour samples were contaminated with aflatoxin, an average of 45.1μg/kg and 66.5μg/kg for aflatoxin B1 and total aflatoxin respectively, most being above reference limit. Contamination was significantly associated with location, economic activity, source, processing and storage time. Through contaminated flours, most of children were exposed to aflatoxin in average of 1.1μg/kg-bw/day, most being exposed above reference limit.

**Conclusion**

Aflatoxin contamination in flour used for complementary feeding is beyond tolerable limits, hence increasing the risk of dietary exposure. Strengthening strategies to control aflatoxin contamination of maize throughout the food chain and teaching parents on processing methods that reduce aflatoxin in cereals are important.

## A17. Improving utilization and demand of MNH/SRH for the reduction of illness and deaths in Rukwa region, Southern part of Tanzania

### Yassin Justine Shonyela^1^, Sikalwanda Kisasu^1^ , Adolf Kaindoa^1^, Martha Lazaro^1^, Oliver Gwaltu^1^, Esteria Kisoka^1^

#### ^1^Plan International, Dar es salaam, Tanzania

##### **Correspondence:** Yassin Justine Shonyela (yassin.shonyela@plan-international.org)

*BMC Proceedings 2023,* **17(13):** A17

**Introduction**

Despite of efforts in place, maternal mortality ratio is still a big concern for the Tanzanian context. About 432 per 100,000 live births occurred in 2012, while in 2015/16 was 556. Similarly, neonatal mortality rate is 25 per 1,000 live births by 2015. Rukwa region, experiences the same challenge including high rate of teenage pregnancies by 29% (2019) and at least 31.4% of births are unassisted by a skilled provider.

**Methodology**

This study aimed to assess the impact of the USR (Uzazi Salama Rukwa) project’s achievements versus the project’s overall intended outcomes, referencing baseline and midterm results in four Councils of Rukwa region. Both quantitative and qualitative methods applied. In-depth interview was successful conducted. A total of 1,399 respondents (aged between 15- 19 and WRA (Women of Reproductive age) aged 20-49) were involved in the study. Data analysis was done using Stata version 15 (STATA Corp Inc.,). Ethical clearance was approved by National Health Research Ethics Review Committee.

**Results**

Majority of respondents; 889 (98.3%) WRA reported to have accessed ANC (Antenatal Care) services from the health facilities. Many of these attended at least once 849 (99.3%) and by skilled personnel (96.2%). The study noted an increase in ANC attendance by 12% from the baseline. Awareness of danger signs during the continuum of care was also assessed. Among 904 women, 49.2% reported to know any danger during pregnancy, 42.2% during delivery, 38.8% after delivery, and 34.1% in new-born. Facility services improved by 34% from baseline. Additional, 96.5% of 890 WRA reported the benefits of having child spacing for about 2-3 years.

**Conclusion**

Study revealed, some men tend to send their wives to give birth at in-law’s house. A recommendation is made for future interventions to include targeted education and sensitization efforts to ensure that all men take an active role during pregnancy, child birth and post-delivery for better maternal and neonatal outcomes.

## A18. “Kagera model” to improve sample collection among eligible clients for genotypic antiretroviral resistance test (GART)

### Emanuel Sarakikya^1^, Suzy Butera^2^, David Temba^1^, Goodluck Ngoda^1^, Kenny Makanyanga^1^, Paschal Pagali^3^, Violeth Mkebezi^2^, Renatus Francis^2^, Isaac Laizer^1^

#### ^1^Management and Development for health, Dar es salaam, Tanzania; ^2^Bukoba Regional Referral Hospital, Kagera, Tanzania; ^3^Kagera - RAS Office, Kagera, Tanzania

##### **Correspondence:** Emanuel Sarakikya (esarakikya@mdh.or.tz)

*BMC Proceedings 2023,* **17(13):** A18

**Introduction**

According to WHO, third line ART is regimen given to PLHIV failing in second line ART. GART has to be done before third line ART initiation. Tanzania Ministry of health has set a model of which clients who are identified eligible for GART are to be referred to Regional referral Hospital for GART sample uptake and further investigation. Kagera model provide a better approach to improve further sample uptake.

**Methodology**

In Kagera Model, Regional Multi-Disciplinary team (RMDT) centrally identify client who are on 2nd line ART with persistent high viral. Then RMDT visit a site for client review collaboratively with Council and Facility MDT aiming at skills capacitation and sustainability. The collected sample will then be processed at a near Laboratory by technical person from R-MDT while training Laboratory member at a site. Processed sample will then be packed and transferred to PCR Lab for GART testing.

**Results**

Before approach of Kagera Model, the regional GART uptake was at 5.3% in March 2021 among eligible clients. After Kagera model implementation the uptake improved to 92% in September 2021 and as of June 2022 the sample for GART collected among eligible attained to 100%. After Kagera model new 6 Laboratory technicians were capacitated in processing of buffy coat sample ready for GART and thus sustainability of sample preparation is observed. Similarly, facility and council MDT have been capacitated to offer better quality service of clients failing in 2nd line and EAC in general.

**Implication**

In hard-to-reach environment, low social-economic status PLHIV client on ART failing on 2nd line, RMDT has managed to collect all sample eligible for GART under “Kagera Model” application. It is recommended Ministry of health to review sample collection model for GART to reach people with social-economic barrier.

## A19. Effect of covid-19 vaccination surge to maximize vaccine uptake in Tabora region

### Benson Mturi^1^, Aminiel Ngomuo^1^, Adam Mrisho^1^, Catherine Bunga^1^

#### ^1^Management and Development for Health (MDH), Dar es salaam, Tanzania

##### **Correspondence:** Benson Mturi (bmturi@mdh.or.tz)

*BMC Proceedings 2023,* **17(13):** A19

**Introduction**

WHO recommends, 70% of adults 18+ years are to be fully vaccinated by Mid-2022. Tabora with a target population of 1,486,877, by 13th June 2022 only 223,031 (15%) were fully vaccinated. This low coverage was contributed by low number of vaccinators involved on routine vaccination days, lack of single dose vaccines (JJ), less engagement of LGAs and misconceptions on Covid-19 vaccine. This necessitated urgent approach to improve vaccination uptake.

**Methodology**

R/CHMT in collaboration with MDH organized surge to improve vaccination uptake in Tabora. Community sensitization meetings were conducted engaging influential government, political and religious leaders. Timely ordering of 453,000 (JJ) doses from IVD together with identifying teams of vaccination from 304 health care facilities. These teams in collaboration with CHWs, conducted door to door sensitization vaccination across the region. Daily monitoring on vaccines consumption from health facilities was done to identify outreaches not progressing well and timely intervening in sensitization.

**Results**

During surge, total of 600 vaccinators form health facilities were deployed in additional to 127 vaccinators existed before surge. Main approach used to reach eligible clients for vaccination was through door to door, and succeed to vaccinate 121 outreaches. Participatory involvement from government and community leaders of all 8 councils during public awareness and sufficient amount of vaccine was effective to improve drastic vaccination coverage from 223,031 (15%) to 886,365 (60%) of targeted population as of 19th July 2022 after successfully completion of two surges executed in mid-June and mid-July.

**Conclusion**

Engaging Government and local influential leaders who are able to speak mother tongue language when explicate the importance of vaccination. Adequate number of vaccinators is paramount in improving vaccine uptake. Still there is high demand of vaccination cards which in some areas affected uptake of vaccine.

## A20. Prevalence, outcome and factors associated with dysmenorrhea among secondary school students in Nyamagana district, Mwanza

### Stephen William Kazimir^1^

#### ^1^Management and Development of Health, Dar es salaam, Tanzania

##### **Correspondence:** Stephen William Kazimir (malimi.william@gmail.com)

*BMC Proceedings 2023,* **17(13):** A20

**Background**

Dysmenorrhea is the painful menstruation which broadly categorized into primary and secondary dysmenorrhea. Dysmenorrhea is very common gynecological problems among young female which affects both psychological and physical aspect of a patient and is among the common cause of absenteeism in school. Despite dysmenorrhea being among the common gynecological problem still there few data concerning its prevalence or the impacts it causes in secondary school students especially in Mwanza region.

Objective: To determine the prevalence, outcome and factors associated with dysmenorrhea among secondary school students in Nyamagana district, Mwanza.

**Methodology**

This study was a cross sectional study involving secondary school students in Nyamagana district. A total of 200 students were involved in this study from five different secondary schools and data was obtained through pretested questionnaires. Data concerning socio demographic, menstruation characteristics and factors associated with dysmenorrhea were obtained. The data was analyzed using frequencies, mean and percentage and for data requiring association, chi square was used, taking p value ≤ 0.005 to be statistically significant.

**Results**

A total of 200 students participated in this study in which 77% reported to have dysmenorrhea. Dysmenorrhea was associated with young age of menarche (p= 0.025) and one member of family experiencing dysmenorrhea (p=0.001). A total of 58 (29%) students reported to miss one or more days in school due to dysmenorrhea and the commonest method used to relief menstrual pain was medication (44.8%) and paracetamol was reported to be used with most of the students.

**Conclusion**

Dysmenorrhea continues to be a health issue that has a substantial impact on female students' attendance at school. More interventions at the school level is needed to ensure that students have the necessary knowledge and are able to seek medical treatment early.

## A21. Peer parents are “part & parcel” in improving HIV viral load suppression among pediatric aged 1-4 years in Tabora region

### Monica Richard^1^, Tausi Kibonge^2^, David Sando^1^, Allan Sayi^1^, Adam Mrisho^1^, Michael Mahande^1^, Amani Shao^1^, Goodluck Lyatuu^1^, Marcelina Mpandachalo^3^

#### ^1^Management And Development for Health, Dar es salaam, Tanzania; ^2^Local Government Authority,Nzega Dc, Tabora, Tanzania; ^3^Regional Adminstrative Secretary,Health-Tabora, Tabora, Tanzania

##### **Correspondence:** Monica Richard (mrichard@mdh.or.tz)

*BMC Proceedings 2023,* **17(13):** A21

**Introduction**

Global Pediatric Treatment goal in HIV is to attain 95% Viral load suppression. In 2019, Viral load suppression among pediatrics (1-4years) in Tabora region was low at 71%, far from set target of 95%. Reasons identified were caregivers HIV status and emotional readiness, lack of disclosure to family members and lack of parental follow- up on medication adherence. Peer parent approach rolled out to address these gaps.

**Methodology**

Using CTC 2 database, data analyzed on adherence to appointment and ARV regimen during October- December 2019 among unsuppressed pediatrics. In January 2020,40 facilities selected aiming to establish pool of peer parents/caregiver for children with latest high viral load, previous high viral load but suppressed and ever suppressed. 120 peer parents/caregiver trained on parental care, Basic HIV Knowledge, disclosure and ARV adherence. Pairing among parents done for experience sharing, complimented by home visit to household of unsuppressed child.

**Results**

Observed remarkably improvement in viral load suppression among pediatrics (1-4years) from 77% in June 2020, 86% in June 2021 to 95% in June 2022. Managed to reach 557 caregivers and paired them with trained 120 peer parents across 8 district councils. Through this period also observed that optimal ARV regimen coverage and adherence to appointment improved from 55% and 61% in Oct 2019 to 98% and 95% in June 2022 respectively. Moreover, there is an increased level of knowledge to caregivers on basic HIV concepts and disclosure techniques to family members and improved parental follow up on medication adherence.

**Conclusion**

Engaging peer parents through pairing with caregivers of unsuppressed children is paramount in improving viral load suppression among pediatrics (1- 4years). Home visit to unsuppressed child complimented the peer parents’ approach. This approach could be replicated to similar settings with aim to improve quality of life to this age group.

## A22. The use of electronic logistic management information system (elmis) for improving multi month dispensing among people living with HIV lessons learned from Tabora region

### Abubakari Abeid Mariri^1^, Vincent Ngelageza^1^, Baraka Mgiriwa^1^, Adam Mrisho^1^, Tarcila Ewald^1^, Amani Shao^1^, Fredrick Mtunguja^1^

#### ^1^Management and Development for Health, Dar es salaam, Tanzania

##### **Correspondence:** Abubakari Abeid Mariri (amariri@mdh.or.tz)

*BMC Proceedings 2023,* **17(13):** A22

**Introduction**

World Health Organization (WHO) recommendation for “test and treat” initiative aimed for early initiation of antiretroviral therapy for people living with HIV (PLHIV) resulted in client’s congestion and high demand of ARVs that adversely impacted multi-months dispensing (MMD). We aimed to improve MMD uptake by training health care workers on how to effectively use electronic logistic management information system (eLMIS) in Tabora region.

**Methodology**

In February 2020, MDH in collaboration with regional and council health management teams (R/CHMT) identified 120 facilities with low uptake, defined as number of clients on MMD divided by eligible clients for MMD. To improve multi month dispensing of anti-retroviral drugs uptake the two teams resorted to training of health care workers on the input of the consumption data in to eLMIS and timely ordering of ARVs. We provided supportive supervision and mentorship during implementation of the intervention.

**Result**

By the end of September 2019, a total of 31,443 clients from all 152 facilities were eligible for MMD but only 18,194 (57.9%) were on MMD. In February 2020, before the intervention, 120 health care facilities had 31161 clients eligible for MMD but only 15256 (48%) were on MMD (low uptake of MMD). By the end of September 2021 after the intervention, 120 health care facilities had 56,858 clients eligible for MMD and 50743 (89.2%) were receiving MMD. The contribution of nearly double the increase of clients on MMD due to improved availability of ARVs throughout.

**Conclusion**

Training coverage in facilities with high number of eligible clients for MMD contributed to uptake of MMD after intervention. Trained of health care workers, supportive supervision and mentorship following the introduction of eLMIS improved skills to work with the system to improve MMD uptake through availability of ARVs throughout.

## A23. Patterns and trends of antibacterial resistance before and during the COVID 19 era (2019-2021) at Mbeya zonal referral hospital

### James Andrew Hellar^1^, Gideon Kwesigabo^1^, Azma Simba^2^, Rogath Kishimba^2^

#### ^1^Muhimbili university of Health and Allied Sciences, Dar es salaam, Tanzania; ^2^Ministry of Health, Dodoma, Tanzania

##### **Correspondence:** James Andrew Hellar (hellarjay@gmail.com)

*BMC Proceedings 2023,* **17(13):** A23

**Introduction**

Internationally, there is a growing concern over antimicrobial resistance (AMR) which is currently estimated to account for more than 700,000 deaths per year worldwide. If no appropriate measures are taken to halt its progress, AMR will cost approximately 10 million lives and about US$100 trillion per year by 2050.

**Methodology**

This was a retrospective cross-sectional study aimed to assess antibiotic susceptibility patterns, trends and associated factors of clinical bacterial isolates obtained from Mbeya zonal referral hospital from 2019 to 2021. For 1380 patients and 8368 samples, comparisons of resistance proportions between Pre and COVID-19 eras were made. Each sample and pathogen-antibiotic combination was treated independently regardless originating from the same patient. Antibacterial resistance trends from all selected prioritized specimens and Pathogen- antibiotic combinations in 2019 were used as baseline data.

**Results**

High resistance rates among E. coli and other Gram-negative bacilli with proportions of 24.33% and 22.09% among all resistant strains isolated were observed. The risk of resistance among isolated bacteria from 2019 to 2021 is significantly high among Acinetobacter species, Citrobacter species, Enterobacter species, E. coli, Neisseria gonorrhoeae and Methicillin Resistant Staphylococcus aureus (PR 1.29,1.29,1.24,1.89,1.38 and 1.23, as compared to proteus species (P<0.05). Antimicrobial resistance prevalence to selected pathogen-antibiotic combinations is significantly high during the COVID-19 pandemic with a rise of 14.5% in 2021, (P<0.05) as compared to 2019. High resistance rates from commonly used antibiotics were observed with Cephalosporin’s, Macrolides, Penicillin’s and sulfonamides showing resistance rates of 70.6%,62.2%,83.0% and 78.6% respectively. Age group category of 21-59 is at higher risk of developing antimicrobial resistance (36.74%), as compared to other age groups (P<0.05).

**Conclusion**

Antimicrobial resistance trends have risen significantly for the years 2020 and 2021 by 13% and 14.5% respectively and misuse of antibiotics may have been as a result of this observed increase. Resistance to commonly used antibiotics such as penicillin’s, cephalosporin’s, macrolides and sulfonamides against frequently isolated bacterial isolates alarms for misuse of commonly prescribed antibiotics.

## A24. Demonstrating how public-private health systems strengthening through digital health increases coverage of RMNCH services in Kibaha district Tanzania

### Fatma H. Mahmoud^1^, Neema Kafwimi^2^, Kenneth Ogendo^2^, Oscar Lugumamu^1^

#### ^1^Apotheker Consultancy, Dar es salaam, Tanzania; ^2^D-tree International, Dar es salaam, Tanzania

##### **Correspondence:** Fatma H. Mahmoud (fmhassan19@gmail.com)

*BMC Proceedings 2023,* **17(13):** A24

**Background**

Lack of coordination among private and public providers - Accredited Drug Dispensing Outlets (ADDOs), and Health Facilities and Community Health Workers (CHWs) respectively - means that often times, people fall through the cracks of the care system which leads to thousands of preventable deaths and unwanted pregnancies each year. The Afya-Tek program addresses these challenges by leveraging health systems strengthening and digital technologies to produce evidence for impact and scalability.

**Methodology**

The Afya-Tek digital solution was established in conjunction with the government to addresses the fragmentation of the primary healthcare system, by bridging together nearly 500 CHWs, ADDOs and health facility staff in Kibaha, Tanzania, using a people-centered approach to increase access to prompt, high quality, and individually-tailored health care. The program applied a human-centered design approach that involved the program beneficiaries, health providers, and government officials; developing a system that’s in line with government requirements and end-user priorities.

**Results**

Over 200k individuals in Kibaha have been enrolled in the program, of which 30% are pregnant and postpartum women, children and adolescents. The CHWs have conducted over 160k visits to provide health services which include screening pregnant and postpartum women, children, and adolescents for danger signs. This has resulted in 24k referrals to Health Facilities and 12k linkages to ADDOs, with high completion rates at 85%. ADDOs have conducted over 35k screenings, and issued over 3k referrals to Health Facilities. The results show improved coordination of care where individuals are able to access prompt, high quality, and individually-tailored health care.

**Conclusion**

The program has illuminated the need for health systems strengthening to escalate efforts towards achieving UHC. The results show that technology contribute towards saving lives and offering patient-centred RMNCH services. However, to achieve maximum impact, the system needs to be scaled to other districts, with close collaboration with the government.

## A25. “…. where will I put them for my husband not to see them?”: Refusing ART initiation in fear of disclosure

### Tusajigwe Erio^1,3^, Josien de Klerk^2^

#### ^1^PharmAccess International/AIGHD/University of Amsterdam/NIMR, Dar es salaam, Tanzania; ^2^Leiden University College/AIGHD, Leiden, Netherlands; ^3^PharmAcess International/Amsterdam Institute of Global Health and Development, Dar es salaam, Tanzania

##### **Correspondence:** Tusajigwe Erio (t.erio@aighd.org)

*BMC Proceedings 2023,* **17(13):** A25

**Background**

After decades of the HIV and AIDS pandemic, ART became a rescuer and transformed HIV/AIDS from a terminal illness to chronic disease. The recent WHO global goal 95 95 95, aims to end the pandemic by 2030, and ART is the main catalyst for it. ART has proven to help the infected live longer, improving their quality of life and of those affected by it, and now ART is used for prevention. ART symbolizes victory in the medical field, however, in the communities, it may symbolize defeat and induces fear. The aim of this study is to examine the acceptability of early treatment initiation among people who tested HIV positive in the test and treat project conducted in Shinyanga, Tanzania.

**Methodology**

We conducted an ethnographic study among HIV-infected people who participated in the test and treat project in Shinyanga, Tanzania from 2016 to 2018. We conducted observations during testing activities in health facilities and in the communities, we conducted informal talks and in-depth interviews with healthcare providers and HIV clients of the project. We conducted thematic analysis to learn the acceptance of early treatment initiation following the government policy of treat all.

**Results**

Our findings show that the acceptance of early treatment initiation varied across gender and the acuteness of the disease. Acceptance was higher among men than their female counterparts. Most men happened to test at their latest stage of the disease, hence accepting treatment immediately. As for women, most happened to get HIV testing at their earliest, especially during pregnancy. Most women refused or delayed treatment initiation. They perceived treatment initiation as a cause of disclosure, something which is feared to endanger their dignity and sexual partnerships. The socio-cultural complexities of gender norms around sexual practices, marriage, and disclosure complicated the early treatment initiation among women.

**Conclusion**

To increase acceptance of early treatment initiation, campaigns on treatment as prevention should highly be re-directed to the general population and address norms around discordancy. When people are highly aware and knowledgeable about the current evolvement of HIV treatment, it will increase acceptance of the partner’s status among couples. Hence, reduce the fear of disclosure and increase the uptake for early treatment initiation.

## A26. Novel AI and android based method for automatic malaria parasite detection

### Sydney Kibanga^1^, Firmatus Joseph^1^, Innocent Matumba^1^

#### ^1^St. joseph college of engineering and technology, Dar es salaam, Tanzania

##### **Correspondence:** Sydney Kibanga (sydneykb38@gmail.com)

*BMC Proceedings 2023,* **17(13):** A26

**Introduction**

Malaria remains a major public health problem in sub-Saharan Africa, with approximately 1 million deaths and more than 400 million cases a year. In Tanzania, over 95% of the 37.4 million people are at risk for malaria infection. The disease is responsible for more than one-third of deaths among children under the age of 5 years and for up to one-fifth of deaths among pregnant women.

**Methodology**

Light microscopy is used for malaria diagnosis. However, it is time-consuming, Expensive and quality of the results depends on the skill of microscopists. Automating malaria parasite detection is a promising solution where an android app is used to capture the images of blood sample. The images are then being processed by Deep learning model that has been trained using YOLO algorithm to detect plasmodia parasites. For this to work properly a magnifying lens may be needed such the model may detect correctly.

**Results**

An Android mobile application is used with the help of magnifying lens, which makes smartphones an affordable yet effective solution for automated malaria light microscopy. This approach is simple to use hence it does not require an expertise to identify the infected blood also reduces the cost of purchasing microscope, therefore it can be used in rural areas, homes or any other healthcare centers. Also, after a successful analysis data can be stored into database through IOT therefore the system can be applied to analyse sample in areas or regions with high infections without actually sending a laboratory technician.

**Conclusion**

The need for the trained personnel can be greatly reduced with the development of an automatic, accurate and efficient system.

## A27. Implementation of national covid19 systems in Tanzania

### Wilfred Senyoni^1^, Tuzo Engelbert^1^, Erick Chingalo^1^

#### ^1^HISP Tanzania, Mazinde Street, Dar es Salaam, Tanzania

##### **Correspondence:** Wilfred Senyoni (senyoni@hisptanzania.org)

*BMC Proceedings 2023,* **17(13):** A27

**Introduction**

In Tanzania, the first case of COVID-19 was reported in March 2020 where the government initiated several efforts and strategies to control the pandemic. Testing for COVID19 for travelers and vaccination of the population were one of the key measures to contain the virus spread by residents and foreigners. To achieve this process, the Ministry of Health (MoH) thought of coming up with an electronic for COVID19 testing and certification of results as the basis of verifying COVID19.

**Methodology**

HISP Tanzania in collaboration with MoH and other stakeholders gathered and documented requirements prior to systems development. HISP Tanzania developed a public web-based system for booking and certification, also secured DHIS2 for sample collection, laboratory information as well as vaccine provision at health facilities. This was followed up with system testing and verification, and later deployment along with system documentation.

**Results**

The MoH launched COVID19 applications (Pima COVID, AfyaMsafiri, and ChanjoCOVID) to users whereby Pima COVID facilitated travelers to book for COVID19 test and issued results after being tested Negative. AfyaMsafiri served as a surveillance tool to different Point of Entry where each returning resident or foreigner was required to be screened for COVID19 signs and symptoms, and those from high-risk countries were tested using Rapid test and given a certificate of COVID19.

**Implication**

With the initiatives the Government of Tanzania has taken to control the pandemic, the MoH, HISP Tanzania and other stakeholders are working to make sure the COVID19 systems and data are being integrated with other community systems that Tanzania is a member e.g., East Africa Community, SADC, etc. Currently, Tanzania has managed to integrate PIMA Covid and ChanjoCovid into the East Africa System to make sure that tested and Vaccinated Clients are regionally.

## A28. Adolescent-nutrition-and-health: Formative assessment of the school-health environment and programs in Ethiopia, Sudan, and Tanzania

### Amani Tinkasimile^1^, Mary Mwanyika Sando^1^,Wafaie Fawzi^3^, Dominick Mosha^1^, Isaac Lyatuu^1^, Frank Mapendo^1^, Mwita Waibe^4^, Mashavu Yussuf, Japhet Killewo^2^

#### ^1^Africa Academy for Public Health, Dar es salaam, Tanzania; ^2^Muhimbili University of Health and Allied Sciences, Dar es salaam, Tanzania; ^3^Harvard T.H.Chan School of Public Health, Massachusetts, United States of America; ^4^Presidents Office- Regional Administration and Local Government, Dodoma, Tanzania

##### **Correspondence:** Amani Tinkasimile (atinkasi@aaph.ot.tz)

*BMC Proceedings 2023,* **17(13):** A28

**Introduction**

90% of adolescents live in low-and-middle-income countries. Adolescence (10-14 years) is a critical phase for research and intervention. Intervening through schools provides an effective strategy for improving health. There is limited evidence linking policies and school-environments to related nutrition and health outcomes. This formative assessment sought to understand policy surrounding school-health-environments, individual adolescent-health and population-level risk factors relevant to design, delivery and scale-up of nutrition and health interventions through schools.

**Methodology**

Multi-stage cluster random sampling was used to select participating schools and students. Mixed-methods were used to assess adolescent nutrition, health and school-food-environments among 3558 adolescent boys and girls and 52 teachers. National and subnational policies on adolescent-health in relation to school-health programs desk reviews and key informant interviews were conducted.

**Results**

Although national guidelines are in place, only 54%, 14% and 9% of the local authorities in Ethiopia, Tanzania and Sudan had adolescent-specific health-related policy, respectively. None of the schools provided deworming services in Ethiopia as compared to 43% in Tanzania. School feeding was provided in 90%, 24% and 18%; and drinking water in 70%, 71% and 36% of schools in Ethiopia, Tanzania and Sudan respectively. Although adolescents demonstrated understanding of healthy behaviour and nutrition, their practice was limited to their socio-economic status and decision-making involvement.

**Conclusion**

Improving school age adolescent-health and nutrition surpasses school environment and policy. It requires interventions through curricula and community engagement, with results frameworks to gage progress.

## A29. Combined effort of clinicians and data officers on improving uptake of tuberculosis preventive therapy in Tabora Region

### Fredrick M Mtunguja^1^, Abubakar Amriri^1^, Vincent Gelageza^1^

#### ^1^Management and Development for Health, Dar es salaam, Tanzania

##### **Correspondence:** Fredrick M Mtunguja (fmtunguja@mdh.or.tz)

*BMC Proceedings 2023,* **17(13):** A29

**Introduction**

It is much Important on Combined Effort between Clinicians and Data Officers since to understand the really performance a data reflection it is key and so important also this will assist on acting with WHO guidelines and Tanzania Ministry of Health which recommends documentation for Data Quality.

**Methodology**

Started by identifying 142 Facilities with low uptake in Tuberculosis Preventive Therapy in Tabora Region and organizing mentorship on how they coordinate and combine efforts to improve Uptake through documentation which can be done by Clinicians, Extraction of eligible list from CTC 2 Electronic Database and triangulate them with what is in the Tuberculosis Preventive Therapy Register which was to be done by Data Officer in order to avoid skipping between source registers, files and Electronic Database.

**Results**

Through identifying facilities with low uptake on providing Tuberculosis Preventive Therapy it has simplified on mentorship arrangement to reach a large number of Clinicians and Data Officers within a short time where we coordinate number of 139 Data Officers and 139 Clinicians and doing mentorship on how they can coordinating two of them by combining effort which will facilitate much on data Triangulation and quality by reviewing Files, Register and Electronic Database after this combined effort the outcome.

**Conclusion**

In accordance with WHO guidelines of 2020 for tuberculosis prevention the Tanzanian Ministry of Health recommends 100% uptake of eligible for tuberculosis preventive therapy for clients who are in Antiretroviral that can be improved through the use of good documentation.

In Tabora Region from October 2019 Clients who are in Antiretroviral and eligible for Tuberculosis Preventive Therapy were 80.45% which was contributed by ImproperDocumentations by Clinicians and Data Officers.

## A30. AfyaToon: Digitalizing health content

### Jofrey S. Amos ^1^, Edwin Ngula Luguku^2^, Alfateresia Mwasagama^3^, Mwnafuraha Adinan^4^

#### ^1^Medical Student at MUHAS and Innovation Lead at Afyatoon, Dar es salaam, Tanzania; ^2^Medical Doctor, Co-founder and CEo at AfyaToon, Dar es salaam, Tanzania; ^3^Medical student at MUHAS and assistant Project Coordinator at Afyatoon, Dar es salaam, Tanzania; ^4^Medical student at MUHAS and Treasurer at Afyatoon, Dar es salaam, Tanzania

##### **Correspondence:** Jofrey S. Amos (segezajofrey@gmail.com)

*BMC Proceedings 2023,* **17(13):** A30

**Background**

AFYATOON company is a group of digital visual animations developers aimed at digitalizing health content and delivering it to the Target population; the adolescents, in the most convenient ways, the basic being visual animation. It was established in Dec 2017. Around 91.6% of Tanzanians obtain health information from health facilities. AfyaToon utilizes hospital waiting areas as one of the channels to disseminate it’s content.

**Methodology**

AfyaToon uses visual art technology including 2D and 3D animations, motion graphic and posters to digitalize health content, presenting it in a story based way to educate and still entertain the recipient through social media platforms and hospital waiting areas. In its recent project; Afyacomic (it's current project) originating from the words afya and comics, it uses comics as a mainstay tool for presenting health information in a story based and entertaining way.

**Results**

Through online projects; one being Hold my hand (Oct 2019), it reached up to 1200 youth raising awareness on early detection of depression. AfyaComics (it’s recent project); has created up to 15 online comics and animations in key areas of cardiovascular, respiratory and diabetes and dental diseases. Also being involved in creating awareness of sickle cell disease which was showcased through AfyaToon online platforms in association with other Sickle cell based organizations, health promotion sector under ministry of health and media including Mwananchi newspaper.

**Conclusion**

Simplicity of health information using visual arts has proved to be a key component contributing to an increased awareness on health issues in the community. Integrating visual arts into hospitals TV screens in hospital waiting areas would help to improve quality of healthcare through health promotion and raising awareness.

## A31. Tracking of key supply chain management performance indicators for optimizing conventional laboratory testing services in Tanzania

### Christopher Henjewele^1^, Yefta Emmanuel^1^, Redempta Mbatia^1^, Benedicta Masanja^1^, Rayton Kiwelu^1^, Moses Madeje^1^, Lilian Shija^2^, Ester Shija^3^, Reginald Julius^3^

#### ^1^Tanzania Health Promotion Support, Dar es salaam, Tanzania; ^2^Centre for Disease Control, Dar es salaam, Tanzania; ^3^Ministry of Health, Dodoma, Tanzania

##### **Correspondence:** Christopher Henjewele (chenjewele@thps.or.tz)

BMC Proceedings 2023, **17(13):** A31

**Background**

Tanzania has made significant strides in strengthening health information systems for timely informing the different program areas. Despite such improvements, data availability and use for supply chain management (SCM) still lag behind. THPS through CDC support implements HIS-MIFUMO project that emphasizes on laboratory SCM data use.

In collaboration with MoH, NACP, MSD, and SCM stakeholders, THPS leverages SCM data and ensures its availability for improved services delivery at testing laboratories.

**Methodology**

Introductory meeting with the MoH for approval was conducted. Review of daily, weekly, and monthly SCM reporting templates from 23 HIV VL/EID conventional laboratories implemented to include SCM data. The key SCM performance indicators reports were monitored and shared with stakeholders including lab commodities managers, national HVL/EID coordinators, IPs, and CDC. Development of SCM analytics tool for real-time data analysis conducted and monitoring the labs reports to ensure completeness, accuracy, and timely submission. Sharing the reports with SCM key stakeholders.

**Results**

Tools integrating SCM indicators for daily, weekly and monthly reporting were approved and data collection commenced on 21st Feb 2022. The SCM logistics analytics tool for real-time analysis of data from the testing laboratories and MSD . The intervention rescued 460 conventional laboratory reagent kits capable of testing 42,780 samples and 7,351 GeneXpert kits from expiry; ensured timely redistribution of 385 reagent kits to testing laboratories. Enhanced uninterrupted testing at the conventional laboratories due to timely availability of reagents.

**Conclusion**

Timely monitoring of SCM KPIs and early warning indicators and data use among supply chain stakeholders ensures constant supply of commodities by improving redistribution of stock imbalances (overstock and understocks) which ultimately improves service delivery at the conventional laboratories.

## A32. Focused intensified facility case finding approach for improving TB case notification Afya Jumuishi Geita

### Florian Mutasingwa^1^, Nicholaus Tarimo^1^, John Msuya^1^, Daniel Mpembeni^1^, Vincent Lazaro^1^

#### ^1^Management and Development for Health, Dar es salaam, Tanzania

##### **Correspondence:** Florian Mutasingwa (fmutasingwa@mdh.or.tz)

*BMC Proceedings 2023,* **17(13):** A32

**Introduction**

The National Tuberculosis (TB) surveys have shown that in Africa, including Tanzania, TB detection and reporting are suboptimal. In 2018 Tanzania National TB and Leprosy program reported that 67,308 (47%) TB cases were missed. Overwhelmed health care providers were noted as a reason for low TB detection and reporting. Towards the end TB strategy by 2030, the focused intensive facility TB case finding for early diagnosis and treatment is inevitable.

**Methodology**

MDH collaborated with Council Health Management Team to introduce an active intensified facility TB case-finding approach. The intervention involved the orientation of health care providers on systematic TB screening using approved tools, and the expected outcomes of the intervention at selected facilities (Chato hospital, Bwanga, Uyovu and Bukombe Hospital). The health care providers were asked and tasked to work beyond their routines to support systematic TB screening for all people who visited health facilities seeking care regardless of presenting symptoms.

**Results**

A total of 1674 patients attended the health care facilities during the period of five days. We screened 1065 (63.6%) people who attended the health facilities for TB regardless of the complaints except for those with physical injuries. We managed to identify 342 (32.1%) with TB-related symptoms. However, from these TB-presumptive patients a total of 64 (18.7%) TB cases were notified and started treatments for TB. We managed to capacitate 32 health care providers with TB screening and management skills. Lastly, the intervention stimulated the facilities to plan on how to organize systematic TB screening during overcrowded days.

**Implication**

The organized intensified active facility TB case finding through systematic screening at all entry points aids in early case detection and treatment. However, the scarcity of health care providers, and multi-tasking of those available challenges the quality of TB screening at the health facilities hence leading to missed TB cases.

## A33. Strengthening uptake of dry blood spot (DBS) through facility led community outreach initiatives in improving early infants diagnosis (EID) in Tabora Region

### Allan D. Sayi^1^, Marcelina Mpandachalo^2^, Adam Mrisho^1^, Goodluck Lyatuu^1^, Amani Shao^1^, Talumba Samatta^1^, Brenda Simba^1^.

#### ^1^Management and Development for Health, Dar es salaam, Tanzania; ^2^RHMT-Tabora, Tabora, Tanzania

##### **Correspondence:** Talumba Samatta (asayi@mdh.or.tz)

*BMC Proceedings 2023,* **17(13):** A33

**Introduction**

Globally, 51% of 1.2 million HIV exposed infants (HEIs) receive HIV testing within 8 weeks. In Tanzania 83% of HEIs enrolled in 2021,70% were tested within 8 weeks. Tabora had ≤ 2 months 89% testing coverage in June 2021. Despite multiple strategies to improve EID, still EID is below national target. We aimed to strengthen through integration of community outreach services to improve EID < 2 months coverage in Tabora.

**Methodology**

Conducted mapping to identify outreach centers and DBS collection experts. Conducted 2-3 zoom sessions and site meeting to 113 providers mentoring on the initiative strategy, DBS sample collection and package. 186 providers from 52 facilities supported transport during DBS collection through initiatives. 183 Expected HEIs living distant from facility settings and missed opportunity identified and shared to health workers. Eligible exposed infants reached at identified centers and visiting their homes.

**Results**

By June 2021 the gap of EID ≤ 2 months coverage 6% below the national target. The initiative started in July 2021 and managed collect 3,203 DBS sample collected ≤ 2 months by June 2022. A total of 513 (16%) out 3,203 collected below 2 months. DBS taken > 2 reduced from 183 in July 2021 to 6 in June 2022. 177 (96.7%) of 183 missed opportunities collected ≤ 2 months. Improved EID ≤ 2 months coverage from 89% in April –June 2021 to 97.8% in April –June 2022 in Tabora.

**Conclusion**

Supporting provider’s transport during outreach-DBS collection helped to reach exposed infants at outreach centers and home settings. The strategy improved EID ≤ 2 months coverage among supported facilities. Strategy may be scaled up to non-supported facilities in Tabora.

## A34. Chini ya buku (below Tsh. 1000/); an initiative to support HIV viral suppression among children and adolescents living with HIV in Kagera region

### Alice Kenneth Simwinga^1^, Loise Odero^1^, Brenda Simba^1^, Fadhili Nyagawa^1^, Rebecca Gwambasa^1^, Talumba Samatta^1^, Phoebe Magatti^1^, Goodluck Lyatuu^1^, Isaack Laizer^1^

#### ^1^Management and Development for health, Dar es salaam, Tanzania

##### **Correspondence:** Alice Kenneth Simwinga (asimwinga@mdh.or.tz)

*BMC Proceedings 2023,* **17(13):** A34

**Introduction**

By 2020, up to 8% of the children and adolescents living with HIV in Kagera had high viral load (>1000 copies/ml), contributed by low awareness and social support on ART adherence. An initiative called “Chini ya Buku, was introduced to raise awareness and motivate adherence to ART. The initiative entailed a simplified communication guide, “Chini ya Buku ndiyo mpango mzima” a Swahili phrase meaning below 1000 is the way to go.

**Methodology**

The Pediatric technical team, worked with peer adolescents and health workers in three ART care health facilities. The initiative aimed to ensure children knew their viral load results trends. Through creating motivation songs as part of entertainment and disclosure steps, establishing a peer to peer WhatsApp group for ART experience sharing and dose reminder platform, conducting peer-to-peer and parents debates and competitions on viral suppression and unsuppressed and linking children and adolescents with psychosocial challenges to social workers for evaluation.

**Results**

Two WhatsApp groups were established with an average of 25-30 members. Two motivations songs were developed, and monthly debates were conducted attended by at least 15 health care workers and two social workers. Over 150 children and adolescents according to their age attended. Cases with challenges were linked to psychological or social services. The overall viral suppression improved from average 92% to 98.5% in 3 sites from quarter Oct-Dec 2021 to Apr-Jun 2022. Across age groups, viral suppression also improved from 94% -100% among 1-4-year-old, from 95%-99% among 5-9-year-old and from 97%-100% among 10-14-year-old and 96% to 98% among 15-19-year-old.

**Implication**

Engagement of parents, children, peer adolescents and health care providers and use of simple communicating language in viral load suppression monitoring is a key to success in attaining viral suppression. The ‘Chini ya Buku’ initiative contributed to increase HIV viral suppression among children and adolescents living with HIV on ART.

## A35. HIV testers engagement in screening for TB signs and symptoms among newly diagnosed people living with HIV: Afya jumuishi project Tabora region

### Jacquiline C. Ngainayo^1^, Beatrice Praise^1^,Ramadhani Shemtandulo^1^, AboubakariMariri^1^

#### ^1^Management and Development for Health, Dar es salaam, Tanzania

##### **Correspondence:** Jacquiline C. Ngainayo (jngainayo@mdh-or.tz)

*BMC Proceedings 2023,* **17(13):** A35

**Introduction**

Tuberculosis is the leading cause of HIV/AIDS related mortality among people living with HIV (PLHIV). World Health Organization recommends quality TB screening in newly PLHIV with 10-15% yield of signs/symptoms among screened. In Tabora region between April-June 2021, TB screening among new PLHIV yielded 6.8% of clients with TB signs/symptoms. We aimed to improve the yield of clients with TB signs and symptoms HIV testers were engaged for TB screening.

**Methodology**

MDH in collaboration with Council Health Management Team carried out on job practical orientation how to conduct TB screening among newly diagnosed PLHIV according to WHO and National TB and Leprosy program approved tools. We engaged 257 facility and community HIV testers. Detailed information on how to collect quality sputum samples, quality sample characteristics, proper labelling of the samples and request forms, sample referral system and linkage to Chest X-Ray for clients with negative sputum results.

**Results**

We observe progressive improvement in proportion of newly diagnosed HIV clients with signs and symptoms of TB from 6.8% in April -June 21 to 14.4% in April-June 22, equivalent to 38% increment of TB cases in this group from 79 to 109 as a result of improved practice by the engaged HIV testers. In addition, the utilization of standardized approved tools for TB screening was observed. All TB cases identified following testing of the clients with TB signs and symptoms were linked in TB treatment.

**Implications**

The increased yield of clients with TB signs and symptoms as a result of engaging HIV testers for TB screening among newly diagnosed PLHIV suggests that implementation of such practice by other implementing partners may improve identification of additional TB cases missed during routine visits at care and treatment centers.

## A36. Effect of smartphone Mhealth intervention on retention to hiv pre-exposure prophylaxis among female sex workers in Dar Es Salaam, Tanzania

### Christopher Mbotwa^1^, Method Kazaura^1^, Elia J Mmbaga^2^

#### ^1^Department of Epidemiology and Biostatistics, Muhimbili University of Health and Allied Sciences,Dares Salaam, Tanzania; ^2^Department of Community Medicine and Global Health, Institute of Health and Society, Faculty of Medicine, University of Oslo, Oslo, Norway

##### **Correspondence:** Christopher Mbotwa (cmbotwa@gmail.com)

*BMC Proceedings 2023,* **17(13):** A36

**Introduction**

Female sex workers are among groups at increased risk of HIV infection. Pre-exposure prophylaxis (PrEP) has been proven to be effective in preventing HIV transmission, but low retention to services poses a challenge to its effectiveness. Innovative interventions to tackle the problem and help achieve universal health coverage goals are called for. The aim of this study was to evaluate the effect of smartphone mHealth application on retention to PrEP services among female sex workers in Dar es Salaam.

**Methodology**

Using respondent driven sampling, female sex workers eligible for PrEP and who owned a smartphone were recruited. All participants were provided with a smartphone-based mHealth app (Jichunge app) designed to promote PrEP use and retention among HIV at risk population. A participant who visited the PrEP clinic within 28 days following their appointment date after initiation of treatment were considered as retained at month 1 PrEP clinic follow-up. The effect of optimal use of Jichunge intervention on retention to month 1 PrEP services was modeled using log-binomial regression.

**Results**

A total of 470 female sex works with a median age of 26 (IQR: 22-30) years were recruited. Overall, 27.7% of female sex workers were retained at month 1 PrEP clinic follow-up. Retention was significantly higher among optimal users of Jichunge app (37.7% vs 16.6%, p<0.001). The results from log-binomial model show that retention at month 1 PrEP clinic follow-up were 1.5 times higher among Jichunge app optimal users when compared to sub-optimal users (aRR=1.5, 95% CI: 1.26-1.84, p<0.001).

**Conclusion**

The use of JichungemHealth application significantly increased retention to PrEP services among female sex workers in Dar es Salaam. The results indicate that use of mHealth interventions hold potential to promote universal health coverage in a cascade of PrEP and other related interventions. mHealth interventions should be incorporated in implementation of the PrEP roll-out to promote utilization of PrEP services among the targeted populations.

## A37. A journey to digitalize integrated management of childhood illnesses tools - a case study from Tanzania

### Deusdedit Mjungu^1^, Method Bulogeleje^1^, Olgah Odek^1^, Melchior Ntakimazi^1^, Deusdedit Mjungu^1^, Martha Masanja^1^, Elena Pantjushenko^2^, Mira Emmanuel-Fabula^2^, Mike Ruffo^2^

#### ^1^PATH Tanzania, Dar es salaam, Tanzania; ^2^PATH Seattle US, Washington, United States of America

##### **Correspondence:** Deusdedit Mjungu (dmjungu@path.org)

*BMC Proceedings 2023,* **17(13):** A37

**Background**

Integrated Management of Childhood Illnesses (IMCI) strategy was developed in 1992 by the WHO and UNICEF to reduce morbidity and mortality among under-five children. Poor adherence to paper based IMCI guidelines has been one of its shortfalls. To address this challenge, Unitaid funded PATH to implement The Tools for Integrated Management of Childhood Illness (TIMCI) project in partnership with the Swiss Tropical and Public Health, Ifakara Health Institute, and the government of Tanzania. This case study therefore outlines steps followed in development of digital IMCI in Tanzania.

**Methodology**

Tanzania digital IMCI was adapted from digital algorithm called ePOCT+. Ministry of Health (MOH) took leadership in identifying a team of experts from Muhimbili University of Health and Allied Sciences (MUHAS), Hubert Kairuki Memorial University (HKMU) and IMCI national trainers to review ePOCT+. The algorithm was reviewed in terms of clinical validity, feasibility in primary care, scope of illnesses, and consistency with national policy and guidelines. The feasibility tests of adapted ePOCT+ were conducted in 20 health facilities in Pwani region in June 2021. The improved algorithm was then piloted in Sengerema, Kaliua and Tanga city councils’ health facilities in October 2021. Health care workers were trained and each of 24 facilities received 2 tablets and 2 pulse oximeters. Routine supportive supervision and mentorship are carried out by PATH and MOH.

**Results**

During October- December 2021, the full digitalized IMCI guideline was completed and in use. Over 2000 sick children were seen using the tool in 24 health facilities. The digital IMCI is in line with the national children management guidelines.

**Conclusion**

Digitalizing treatment guidelines is possible under government leadership, all key stakeholders should be involved. Health Care Workers should be part of the development. Further studies on the advantages of digitalizing IMCI and other guidelines will shed more light on the importance of digital space in health care.

## A38. Prevalence and presentation of urinary bladder cancer at Muhimbili national hospital

### Rashid M. Mdaki^1^, Atupele J Kaponda^1^, Amos Mwakigonja^1^

#### ^1^Muhimbili University of Health and Allied Sciences, Dar es salaam, Tanzania

##### **Correspondence:** Rashid M. Mdaki (mdakizo96@gmail.com)

*BMC Proceedings 2023,* **17(13):** A38

**Introduction**

Urinary bladder disorders are one of the most commonly encountered problems in clinical practice with a high degree of morbidity. These disorders present with a variety of symptoms, signs and their laboratory histopathological findings vary from one disorder to another. A variety of disorders including inflammatory and neoplastic lesions can affect the urinary bladder. Abdominal Ultrasound, endoscopic ultrasound or CT scan are the most useful diagnostic tests and a definitive diagnosis of urinary bladder disorders rests on the histopathological findings and it provides the basis for planning proper management.

**Methodology**

A retrospective study was conducted on patients with urinary bladder biopsies submitted at Central Pathology Laboratory (MNH) for histopathology diagnosis from 2019 to 2020. The demographic and clinical-pathological data was extracted from patients’ request forms and histopathology reports thereafter were filled in a well-designed form. The collected data was entered in computer statistical software (SPSS) version 26 well designed template and be analyzed per each specific objective.

**Results**

A total of 193 study participants were recruited in this study, majority about 53.3% were female whilst males were about 47.7% of the study population. The mean age was 58.55 ± 14.75 years with the mostly predominant age group >50 years which was about 65.8% of participants. The most symptoms presented by majority of the patients was hematuria and abdominal swelling accounting for 99.5% of each. Almost more than half of the patients (63%) had squamous cell carcinoma followed by 29% having transitional cell carcinoma.

**Conclusion**

In this study hematuria and abdominal pain were dominant presentations and also regarding histopathological findings, the most common type was squamous cell carcinoma followed by transitional cell carcinoma. Besides that, from the findings of this study showed that urinary bladder cancer was slightly more preponderance to female than male.

## A39. Improving retention among pregnant and breastfeeding women enrolled in prevention of mother to child transmission using peer mothers in Tabora

### Rahel Bwoki^1^, AbubakarMariri^1^,Brenda Simba^1^,Amani Shao^1^, Benson Mturi^1^

#### ^1^Management and Development for Health, Dar es salaam, Tanzania

##### **Correspondence:** Rahel Bwoki (rbwoki@mdh.or.tz)

*BMC Proceedings 2023,* **17(13):** A39

**Introduction**

African countries highlighted low retention two to three years after enrolment in prevention of mother to child transmission (PMTCT) care ranging from 41% to 74%. In Tanzania retention in PMTCT is at 67%, which is low compared to the national target of 95%. In Tabora 24% HIV+ pregnant and breastfeeding women not are retained in PMTCT, heightening HIV transmission to their children. The reasons for not being retained in to PMTCT programs could be stigma related. We aimed to improve retention among pregnant and breastfeeding women using peer mother approach in Tabora region.

**Methodology**

Women with exposure to PMTCT services from high volume sites with low retention were selected and trained on PMTCTC care services and became peer mothers. Their role was to prepare clinic, identify list of appointment, made home reminders, education on importance of adherence to PMTC services and follow up of missing appointment clients. Follow up of pre-implementation and post implementation data to compare improvement before and after service implementation started.

**Results**

Before intervention October 2018 to September 2019, Tabora had a total of 1467 PMTCT clients. Of these, less than 80% were retained in PMTCT services when there were no peer mothers. For the period of October 2020 to September 2021 after the intervention, 2485 women enrolled in PMTCT services 92% of all 2485 PMTCT clients in the region were retained in care, an increase of 12% from the baseline.

**Implication**

Peer mother mentor initiative contributed in improving retention among women receiving PMTCT services. We recommend similar initiatives to other IP providing PMTCT services.

## A40. Prevalence of hepatitis among opioid drug users receiving methadone assisted therapy in southern highlands zone (SHL), Tanzania

### Adela Peter^1^, David Maganga^1^, Eniko Akom^2^, Jackson Mbogela^1^, Alick Kayange^1^, Hijja Wazee^1^, Cassian Nyandindi^3^, Damali Lucas^1^, Sally Chalamila^1^

#### ^1^Henry M. Jackson Foundation for Medical research International, Walter Reed Program- Tanzania, Mbeya, Tanzania; ^2^International HIV Prevention and Treatment (IHPT) In support of the U.S. Military HIV Research Program (MHRPWalter Reed Army Institute of Research-Tanzania, Walter Reed Army Institute of Research-Tanzania, Dar es salaam, Tanzania; ^3^Drug control and enforcement authority- Tanzania, Dar es salaam, Tanzania

##### **Correspondence:** Adela Peter (ALuswetula@wrp.or.tz)

*BMC Proceedings 2023,* **17(13):** A40

**Introduction**

People with opioid use disorders are more at risk of contracting blood-borne infectious diseases such as HIV, hepatitis B and C. Increasing hepatitis screening, particularly among vulnerable populations, is key to achieve World Health Organization’s goal of eliminating viral hepatitis by 2030. This study aims to estimate prevalence of hepatitis among people with opioid use disorders receiving (MAT) in the Southern Highlands region of Tanzania.

**Methodology**

Data analysis was conducted among opioid drug user attending methadone clinics at Mbeya zonal referral Hospital and Tunduma MAT clinics in SHL from November 2021 to January 2022. All 213 active clients received hepatitis B testing with rapid HBsAg, hepatitis C testing with anti-hepatitis C antibody and HIV testing following the national HIV testing algorithm. we collected demographic detail on all clients and compared positivity rates among demographic groups using chi squire.

**Results**

Overall positivity for viral hepatitis (either hepatitis B surface antigen or anti-hepatitis C antibodies) among PWUD/PWID was 9.3% while of that of hepatitis C infections was 6.7 % and hepatitis B was 6.7%. Prevalence of HCV among PWIDs was found to be around 15.1%. Prevalence of HIV among participants was 13%. Four clients (2%) had HIV and HCV co-infection. The prevalence of viral hepatitis was higher in older age >30 years (p<0.05) while the prevalence of HCV was higher in people who inject drug (p<0.05).

**Conclusion**

Viral hepatitis C prevalence in the study was higher than in the general population but lower than in PWID methadone users. Additional RT-PCR tests are required to determine active infections. More work is needed to remove barriers to hepatitis diagnosis and treatment among opioid drug users to eliminate Hepatitis.

## A41. Occupational and descriptive characteristics of esophageal cancer patients attending Ocean Road Cancer Institute from 2019 to 2021

### Luco Mwelange^1^, Bente E. Moen^2^, Magne Bråtveit^2^, Julius Mwaiselage^3^, Simon H. Mamuya^4^

#### ^1^PhD Candidate - Department of Environmental and Occupational Health, Muhimbili University of Health and Allied Sciences, Dar es salaam, Tanzania; ^2^Centre for International Health, University of Bergen, Norway; ^3^Ocean Road Cancer Institute, Dar es salaam, Tanzania; ^4^Department of Environmental and Occupational Health, Muhimbili University of Health and Allied Sciences, Dar es salaam, Tanzania

##### **Correspondence:** Luco Mwelange (mwelange@gmail.com)

*BMC Proceedings 2023,* **17(13):** A41

**Introduction**

Cancer is a public health problem in Africa, in 2030, Africa will account ¾ of cancer cases and Tanzania has 50,000 cancer cases each year. Esophageal cancer is 6th leading in mortality (500,000 cases), and 604,100 cases per year. Despite known risks: family history, smoking and alcohol use. Recently, occupational and environmental exposures are emerging risk factors. This study aimed to describe occupational and descriptive characteristics of esophageal cancer patients.

**Methodology**

This descriptive cross-sectional study was done in Ocean Road Cancer Institute (ORCI) employing newly diagnosed patients from 2019 to 2021. This study used secondary data, extracted from ORCI electronic system. The following patient’s information were extracted: Age, sex, occupation, region and district of residence, year of diagnosis, smoking and alcohol use, family cancer history, type of cancer and the family relationship. Occupational information were coded using ISCO O8 system. Descriptive analysis and Chi square test were used for data analysis.

**Results**

The included 975 cancer patients, from which 621 (64%) were male. The mean age was 58(14) and 58(15) for male and female respectively. Almost 70% of the patients were coming from Eastern and Northern zones. Over 50% of male and 12% of female patients used alcohol, while 42% and 12% of male and female respectively were smoking. Over 98% of the patients, occupational status was recorded, but out of this,11% were not properly recorded and 15% of smoking and alcohol use were not recorded. The findings, shows that >50% were engaging in agriculture, and 8% had family history of cancer.

**Conclusion**

A high proportion of cancer patients engaging in agriculture activities provide an opportunity to understand other risk factors. Our finding suggests need for improvement in the recording of hospital-based data, this may serve as important source of data for understanding cancer risk factors in the absence of a population-based registry.

## A42. Effect of healthcare utilization on HIV testing and counselling among adults in Magu district Mwanza, Tanzania

### Michael Ngowi^1^, Jim Todd^2^, Ahmed Yusuph Nyaki^1^, Gilbert Waria^1^, Mark Urassa^3^, Henry Lucas Mlay^1^

#### ^1^Kilimanjaro Christian Medical University Collage, Moshi, Tanzania; ^2^London School of Hygiene and Tropical Disease, London, England; ^3^National Institute of Medical Research, Dar es salaam, Tanzania

##### **Correspondence:** Michael Ngowi (michaelngowi123@gmail.com)

*BMC Proceedings 2023,* **17(13):** A42

**Introduction**

HIV Counselling and testing are the recommended best ways against HIV prevention and treatment services. People who are found to be HIV-negative can then access HIV prevention services, and those who are found to be HIV-positive can access antiretroviral therapy (ART). HCT services need to be available and accessible to the entire public to enable their easy utilization. However, many HIV-positive patients are not diagnosed until they go to the hospital for other.

**Methodology**

This was an analytical cross-sectional study, to determine the effect of healthcare utilization on HIV testing and Counselling among adults in Magu district Mwanza, Tanzania it involved the analysis of the 2016-2017 sero survey conducted within the Kisesa Observational Cohort. Descriptive statistics were done to describe the characteristics of the study participants against HIV testing in the past 12 months and the results were presented in tables by using frequency and proportions. A binary logistic regression was used to establish the magnitude and direction of the association by fitting multiple regression models to the HIV testing (ever tested for HIV in the past 12 months). These statistical models/analyses were implemented using STATA version 15.0 (StataCorp. 2017. Stata Statistical Software: Release 15. College Station, TX: StataCorp LLC).

**Results**

The uptake of HIV testing in the past 12 months was 19.1 %. In the adjusted analysis, the result revealed that visited healthcare provider in last 12 months, number of healthcare visits, Hospital outpatient visit, ANC visit, sex, age, marital status, education level, knowledge about HIV transmission, number of sexual partners in the past 12 months, Anticipated Stigma, recommend a friend for HIV testing and counselling and Confidentiality were significant factors for HIV testing in the past 12 months.

**Conclusion**

Regarding the global target, the proportion of HTC service uptake is still low. Many patients, both inpatients and outpatients, have never taken an HIV test. There are a number of characteristics that influence HTC uptake that can direct steps to raise HTC uptake. Confidentiality, anticipated stigma, and recommendations of HTC services were all strongly and positively related with HTC services. To minimize expected stigma, support HTC service recommendations, and maintain confidentiality in the community, among friends, family, and healthcare facilities, actions should be necessary with HTC services.

## A43. Hidden resort: the role of last desk volunteers in scaling up viral load sample collection among people living with HIV in Dar Es Salaam

### Ruzibukya Victor^1^, Happiness Koda^1^, Patrick Mwidunda^1^

#### ^1^Management and Development for Health (MDH), Dar es salaam, Tanzania

##### **Correspondence:** Ruzibukya Victor (vicruziby@gmail.com)

*BMC Proceedings 2023,* **17(13):** A43

**Introduction**

According to the UNAIDS Global Targets of achieving three 95’s by 2030 and as per Tanzania Health Sector HIV/AIDS Strategic Plan IV (2017-2022) by 2022, Dar es Salaam faced obstacles on achieving 3rd 95 specifically on viral load (VL) sample collection. Missed opportunities in Tier 1&2 and Tier 3&4 contributed about 4% and 5% respectively of eligible clients for viral load sample collection with VL coverage of 93% by January.

**Methodology**

By February 2022, 300 last desk volunteers were recruited and oriented to SOP to Tier 1(>1000 clients) and some of Tier 2(>500 to 1000 clients) facilities. Last desk volunteers reviewed clients’ files after services to identify missed services, including viral load sample collection, and then clients were referred back to care providers if they missed opportunities except for treatment supporter drug pickups and on transit visits. Call back was alternatively used to other facilities to.

**Results**

The last desk volunteers reduced the missed opportunities in Tier 1 and 2 from 5019 (4%) to 2% and VL coverage raised from 93% to 95% by the end of March 2022. In June 2022, a total of 174,246 clients’ files were reviewed, among them treatment supporter drug pickups were 1799 (1%), on transit visit were 2446 (1.4%), and missed opportunities in Tier 1&2 reduced to 1464 (0.96%), and Tier 3&4 to 559 (3%) with a viral load coverage of 96%.

**Implication**

There is a need for effective adherence to last desk SOP to decrease missed opportunities for VL and recruiting volunteers with experience in CTC services for effective client review files. The ‘Last desk volunteer’ strategy may be adopted by other CTC to strengthen healthcare services provision on viral load indicators.

## A44. Assessment of proportion risk factors associated with caesarean section surgical site infection at Muhimbili national hospital, Dar es salaam, Tanzania

### Nicodem Komba^1^, Andrew Mgaya^2^

#### ^1^Muhimbili National Hospital – Mloganzila, Dar es salaam, Tanzania; ^2^Muhimbili National Hospital – Upanga, Dar es salaam, Tanzania

##### **Correspondence:** Nicodem Komba (nicodemclemence@gmail.com)

*BMC Proceedings 2023,* **17(13):** A44

**Introduction**

To C/S mothers, surgical site infection (SSI) is a single most factor for postpartum maternal infection, second leading cause of maternal mortality in Africa to 10%. In Tanzania, SSI prevalence is between 10%-48%, contributing to 24% of maternal mortality (Veneranda B et al., 2019). Knowing the determinants and prevalence of SSI is critical towards setting realistic preventive measures as less effort has made towards this in our settings.

**Methodology**

This was a cross sectional study design, using mixed approach, whereby; Post C/S mothers followed up for 28 days from 3rd to 28th day. Health care workers and cleaners WASH practices were observed at Obstetric theatre (OT), Postnatal and labor ward. But also swabbing and culture for high touch surface area were done in OT and postnatal ward. In-depth interview and focused group discussion with HCWs and Cleaners were also done at Muhimbili National Hospital – Upanga.

**Results**

A total of 209 mothers followed up for 30 days, 15 were lost to follow up. The incidence rate of SSI was 11.3%. Superficial SSI 91.2% appeared most, while the rest had deep SSI. Most of the infection developed after discharge 60.2%. All high touch surface area swabbed had significant microbiological contamination of bacteria like Bacillus species, Coagulase negative staphylococcus, Klebsiella Pneumoniae, Pseudomonas aeruginosa, Staphylococcus aureus, Citrobacter freundii, Acitrobacter species, Providencia species, Morganellamorganil, Serratia marcescens and Enterobacter species. But also 81% of Cleaners and HCWs did not adhere to proper hand hygiene practices.

**Conclusion**

The prevalence of SSI at MNH found to be high to Post C/S mothers to about 11.3%, this is far beyond the recommended prevalence of 1% by WHO. HCWs poor adherence to hygienic practices and preventives measures are the prime factors. Ministry of health should urgently start surveillance of SSI.

## A45. The role of peer mother-ward tie and DBS tracking register in early infant diagnosis in Geita region

### Rogega E. Mong'ateko^1^, Rashid Mbeba^1^, Joseph Kaizelege^1^, Nicholaus Tarimo^1^, Nchemwa Daudi^1^, Florian Mutasingwa^1^

#### ^1^Management and development for Health, Dar es salaam, Tanzania

##### **Correspondence:** Rogega E. Mong'ateko (rezekiel@mdh.or.tz)

*BMC Proceedings 2023,* **17(13):** A45

**Introduction**

Of all HIV infections occurring in children, 90% are due to Mother-child transmission. Currently, WHO estimated that 15% of HIV-exposed infants needing testing are tested in the first two months of life. In October 2021, Geita had Early infant diagnosis coverage of 85% this is low compared to standard coverage of 95%. Missing infants for testing necessitated tracking of these exposed infants to ensure that all are reached and tested.

**Methodology**

DBS tracking register was introduced to help capture all HIV exposed-infant and prevent testing missing opportunity. The intervention involved registering HIV exposed infants in DBS tracking register. A date of Birth is documented and expected date of DBS taking is calculated. Ward-tie between peer-mother and infant-mother is done. Thorough follow-up and reminding the mother of exposed infant three times using phone and Physical visitation by Peer-mother is done. Mothers of exposed infants is tracked at fourth, sixth and eighth week.

**Results**

A total of 67 peer mothers in six different councils were tied to different wards around region depending on their area of residence. A total of 2,618 exposed-infants were tracked between October-2021 to April-2022. Implementation of tracking of exposed infants using DBS tracking register has enabled the reduction of DBS taken beyond two months from 70 (19.9%) in October-2021 to 26 (8.9%) in April-2022. EID coverage has improved from 85% in October-2021 to 97% in April-2022. The bond created between peer-mother and mother of exposed infant has eased continuity and retention to care of both the mother and exposed infant.

**Implication**

We have observed an improved Early Infant Diagnosis coverage contributed much by the use of DBS-tracking register and the use of peer mother-ward tie. Also, a significant improvement on retention of Mothers in PMTCT clinics. We recommend the use of DBS-tracking register as an integral component on Improving EID.

## A46. Factors associated with covid-19 vaccine acceptance among pregnant and breastfeeding women in Dodoma city

### Anodi R. Kaihula^1^, Mwajuma Mdoe^2^

#### ^1^Student, University of Dodoma, Dodoma, Tanzania; ^2^Lecturer at the University of Dodoma, Dodoma, Tanzania

##### **Correspondence:** Anodi R. Kaihula (akaihula@gmail.com)

*BMC Proceedings 2023,* **17(13):** A46

**Background**

There remain myths and doubts among the public regarding the safety and effectiveness of the COVID-19 vaccine resulting in hesitancy and poor uptake of the vaccine among the population.

This study aimed to explore the factors associated with COVID-19 vaccine acceptance among pregnant and breastfeeding women.

**Methodology**

This was an analytical cross-sectional study that was conducted at selected RCH clinics at Makole health centre in Dodoma. A semi-structured interviewer-administered questionnaire was used on 174 women who were willing to participate in the study.

Then descriptive and Chi-square test was done to analyse the results of the study.

**Results**

Among 174 study participants, only 69.5% were aged 21-26, whereas 86.2% of the participants were not vaccinated thus the acceptance rate was 13.8% of the study population.

There was a positive correlation between the level of education, previous history of COVID-19 disease and the vaccine uptake at a confidence interval of 95%.

50.6% of the participants believed that they were healthy and didn't need the vaccine, 12.1% were concerned about the adverse effects of the vaccine on their pregnancy and 19.5% thought the vaccine wasn't effective and safe for them.

**Conclusion**

Still, there are myths about the COVID-19 vaccines which tend to increase vaccine hesitancy, especially among pregnant and breastfeeding women.

COVID-19 vaccine health education should be included as a part of antenatal and postnatal clinic health education to provide full and detailed information about COVID-19 vaccine.

## A47. Increased data use practices in health service management in Tanzania: a case study from district health profiles platforms

### Weja Joel^1^, Tumainiel Macha^1^, Henry Mwanyika^3^, Joseph Makaranga^3^, Seif Rashid^2^, Stephano Mugeta^3^, Auson Kisanga^3^

#### ^1^Ministry of Health in Tanzania, Dodoma, Tanzania; ^2^University of Dodoma Tanzania, Dodoma, Tanzania; ^3^PATHTanzania, Dar es salaam, Tanzania

##### **Correspondence:** Weja Joel (wejajoel@gmail.com)

*BMC Proceedings 2023,* **17(13):** A47

**Introduction**

Sound and reliable information is the foundation for making evidence-based decisions. District Health Profile (DHP) is a snapshot representation of key health indicators and provide essential information that informs plans. Studies show little use of data in healthcare to low- and middle-income countries (LMICs). This paper wants to describe the use of DHPs among health managers at district level in Tanzania.

**Methodology**

Mixed-methods approach was conducted from 2nd to 30th August 2021 to health decision makers who have been oriented in DHP. The study used purposive and convenience sampling methods to recruit participants. Key informant interviews were conducted with 35 participants from national and sub-national levels. An online questionnaire was disseminated to 184 HMIS focal people from 184 councils. Content analysis and descriptive statistical m were used to analyze qualitative and quantitative data respectively.

**Result**

This study indicates that majority of participants in the survey acknowledged the existing demand for DHPs in decision-making meetings. The main users of DHPs were identified to be members of CHMTs, development partners, councilors, and research institutions. The main challenges noted on using information from DHPs were limited awareness among health managers, low DHP priority and inadequate resources for development and dissemination of DHPs.

**Conclusion**

The study showed that DHP were useful in fostering data use at the council level. However, extra efforts are needed to encourage development and use of DHP. This study recommends further study to be conducted to find out appropriate ways of influencing use of DHPs to higher level officials.

## A48. Exploring innovative solutions to enhance supportive supervision practices in Tanzania. The need for digitalization

### Stephano Mugeta^1^, Daines Mgidange^1^, Henry Mwanyika^1^, Seif Rashid^1^, Joseph Makaranga^1^, Ntuli Kapologwe ^2,3^, Kengia,James Tumaini ^2,3^, Chrisogone German^4^

#### ^1^PATH Tanzania, Dar es salaam, Tanzania; ^2^University of Dodoma, Dodoma, United Republic of Tanzania; ^3^President Office - Regional Administration and Local Government, Dodoma, Tanzania; ^4^Ministry of Health, Dodoma, United Republic of Tanzania

##### **Correspondence:** Stephano Mugeta (smugeta@path.org)

*BMC Proceedings 2023,* **17(13):** A48

**Introduction**

Supportive supervision is intended to improve performance and motivation of healthcare workers. Supervision activity in many low- and middle-income countries (LMICs) is uncomprehensive with limited coverage, regular, supported, and motivating. Studies show that there is limited published evidence on effectiveness of supervision activities in LMICs. PATH in collaboration with the Ministry of Health and University of Dodoma conducted study to highlight strength and areas for improvements, and to offer further insight into innovations that can improve supportive supervision mechanisms in Tanzania.

**Methodology**

The study adopted a cross sectional quantitative approach and was conducted from June 14 to July 30, 2021, among supervisors and supervisees. We used simple random sampling methods to recruit participants and online questionnaires to collect 506 responses from Regional Health Management Teams (RHMTs), Council Health Management Teams (CHMTs) and Health Facility (HF) in charges. We used descriptive statistical methods to analyze data.

**Results**

The findings indicated that written plans and budgets were in place and for supervision activities according to MoH guidelines. The study findings showed that the entire CHMTs and RHMTs were involved in planning supervision visits in which data from DHIS2 and previous supervision reports were used to plan supervision visits. Furthermore, the findings showed that checklists were consistently used. However, long checklists and inadequate skills by some users limited the effective use of checklists. The study also found that notifications in the form of phone calls, WhatsApp groups, and letters were sent to the responsible persons before supervision visits.

**Conclusion**

This study highlights the need for policymakers to adopt innovative supervision approaches. Our findings suggest adoption of digital technologies that focus on supportive approaches, quality assurance and problem solving. We recommend further research to be conducted to better understand how these digital tools can be implemented in LMICs.

## A49. Promoting healthy population through an equitable access to health information in Tanzania

### Jonia Bwakea^1^

#### ^1^Pharmaccess International, Dar es salaam, Tanzania

##### **Correspondence:** Jonia Bwakea (j.bwakea@pharmaccess.or.tz)

*BMC Proceedings 2023,* **17(13):** A49

**Introduction**

By January 2021 there were 50.1 million mobile connections which is a coverage of 82% of the total population. Meanwhile the community of internet users is fast growing and it was 15.1 million. Increasing access to quality healthcare starts with people having right information to make them aware on when to seek medical help. Despite increase in smart phone penetration in Tanzania, hardly people have an access to relevant platforms to rightful health information. There is an unmet need for accurate and reliable health information amongst Tanzanian consumers. Lack of information leads to anxieties, uncertainties in making decisions concerning their health and wellbeing at large.

**Methodology**

PharmAccess Tanzania has developed and launched a Tanzanian online health information platform – www.jijali.tz, specifically designed, developed, and maintained to best meet local needs for health, healthcare, and health financing information. www.jijali.tz is to become the go-to place for health-related information for Tanzanian consumers with a wide range of health topics. Moreover, the platform intends to grow exponentially in terms of technology by connecting patients, other sets of consumers to carter not only health information needs but also access online medical services by chatting or calling with medical doctors, counsellors and mapping of healthcare facility locations, their services and mode of payment across the country.

**Results**

Up to July 2022, there were over 17,000 organic impressions translating to number of times viewers searched for contents in www.jijali.tz . These are derived from a total of 482 clicks that mainly based on maternal and child health such as symptoms of delivery, general knowledge around breasts, first aid and skin care. At this new early stage since the website launch, it is technically positioned as among of 10 top searched website (content wise) in the country with 3% CTR.

**Implication**

Our journey towards UHC cannot be achieved without ensuring people have right health information to make informed decision on accessing healthcare. With this beautiful beginning, the site gears to reach and open more free access to local Tanzanian’s to a privilege of precise and concise health information for free in both Swahili and English to improve their knowledge, awareness, complement their adherence, treatment journeys and access of right health facilities locations and services.

## A50. Digitalization as an enabler to making quality improvement measurable, standardized, efficient and interactive

### Asha Mkamba^1^, Piensia Nguruwe^2^, Mwanaali Haji Ali^1^, Peter Risha^2^, Suleiman Simai Jaffar^1^

#### ^1^Ministry of Health, Zanzibar, Tanzania; ^2^PharmAccess Foundation, Dar es salaam, Tanzania

##### **Correspondence:** Asha Mkamba (ash.mk66@gmail.com)

*BMC Proceedings 2023,* **17(13):** A50

**Introduction**

The Ministry of Health (MOH) has been engaging stakeholders to undertake initiatives to improve the quality of healthcare. The majority of these initiatives are vertical, use different tools and approaches, making MOH coordination difficult. To strengthen coordination and promote the use of data, the MOH has developed a digital quality improvement model with the objective of having one approach that is used by all stakeholders and make data available for planning. The model is also connected to an interactive quality platform that motivates facility staff to address quality gaps.

**Methodology**

The MOH engaged with stakeholders to develop standards guidelines for performance rating of the quality of healthcare delivery which are aligned to MOH’s approved best practices and to internationally accredited standards of care. Subsequently, a digital mobile phone based rating tool was developed and used by trained assessors for performance rating. The report and quality improvement plan (QIP) are generated and shared with both the facility and MOH. Furthermore, the QIP is linked to an interactive digital quality platform which has an online library and a “chat box” to virtually support facility staff during implementation of the QIP.

**Results**

About 180 facilities have already been rated using the tool. The rating process has been made more cost-effective as one assessor can rate one facility per day compared to the earlier practice where two assessors were required. The report and QIP are also shared with the MOH HQA and interested stakeholders for coordination and planning. MOH has leveraged resources from stakeholders to address on IPC and RMNCH gaps identified during this exercise. 65% of connected facilities are actively using the quality platform for QIP implementation resulting into faster improvements.

**Conclusion**

The model enables MOH and stakeholders to access and use reports to identify common gaps and prioritize resources for improvement.

## A51. Use of Digital quality platform to support quality services for private health facilities in Zanzibar

### Dua Suleiman^1^, Peter Risha^2^, Mohammed Sheik^1^, Piensia Nguruwe^2^

#### ^1^Private Hospital Advisory Board, MOH Zanzibar, Tanzania; ^2^Pharmaccess Foundation, SafeCare program, Dar es salaam, Tanzania

##### **Correspondence:** Dua Suleiman (suleimandua@gmail.com)

*BMC Proceedings 2023,* **17(13):** A51

**Introduction**

The Private Hospital Advisory Board (PHAB) is a legal Institution, established under the Zanzibar law number 4 of 1994. The main responsibility of the Board is to oversee the operation of private health facilities and has undertaken several intiatives to ensure licensed facilities deliver patient centered care. Recently the Board have enrolled facilities in performance rating exercise and support owners and managers to address the identified quality gaps.

**Methodology**

About 150 owners and in charges from 75 facilities have been sensitized on the patient centered care and then evaluated facilities performance using the MOH digital performance rating tool. Selected staff from each facility were trained on the use of digital quality platform to guide implementation of respective quality improvement plan (QIP). PHAB used management quality platform to monitor and benchmark performance. The PHAB then used the data to allocate resources, plan for supportive supervision and motivate facilities to accelerate completion of QIP.

**Results**

Up to June 2022 Board covered a total of 116 health facilities. In order to ensure services are provided with the best quality. The findings revealed 2 (2.6%) facilities had achieved Level 3 rating, 18 facilities (24%) have got Level 2 and 52 facilities (69%) level 1, and more than 90% of facilities are actively engaged in QIP. The QIPs were immediately shared with owners in order to improve their services.

**Implication**

The results suggest that applying digital platform can motivate changes in health care delivery and improve overall quality. Therefore, sustainability of platform will improve services, thus increase health quality services of private health in Islands. The system is very promising for PHAB to manage and monitor health facilities and service delivery in Zanzibar.

## A52. Developing digital health solution to enhance supportive supervision practices: Experience from Tanzania

### Chrisogone Justine German^1^, Ilvanus Ilomo^1^, Henry Mwanyika^2^, John Gamaliel^2^, Eliudi Eliakimu^1^, Seif Rashid^2^, Eric Kitali^3^, Geofrey Gundah^2^, Caroline Amour^4^

#### ^1^Ministry of Health, Dodoma, Tanzania; ^2^PATH Tanzania, Dar es salaam, Tanzania; ^3^President Office-Regional Administration and Local Government, Dodoma, Tanzania; ^4^Kilimanjaro Christian Medical College, Moshi, Tanzania

##### **Correspondence:** Chrisogone Justine German (drxgone@gmail.com)

*BMC Proceedings 2023,* **17(13):** A52

**Background**

Application of digital technology in the healthcare industry is happening all over the world. Tanzania is making the transition to digital models of operation by developing a digital solution for supportive supervision known as Afya Supportive Supervision System (AfyaSS). AfyaSS is a locally developed system for planning and conducting supervision activities. The system addresses the challenges of supervisions such as inadequate coordination, multiple tools, and inadequate follow up action plans. We present an approach used in developing AfyaSS, a digital system that enhance data use practices.

**Methodology**

AfyaSS was designed using a human-centered design approach. Beginning in 2018, the government of Tanzania and PATH embarked on a participatory design process that included a desk review of existing guidelines and tools, field visits, stakeholder workshops, and user advisory groups to identify common challenges and system requirements of the future AfyaSS.

**Results**

AfyaSS is rolled out to 26 regions where supervision visits are now easily coordinated. A total of 86 National and regional ToTs were trained for technical support to regions and councils. As part of implementation of AfyaSS, different materials were developed including comprehensive user manual, facilitator’s guide, training slides, and video tutorials. The system has consolidated various checklists for different health areas, dashboard features to monitor progress toward health system goals, and action plans linked to data from previous visits.

**Conclusion**

This digitalization process has the potential to transform the current supervision processes by empowering and motivating health workers and managers to apply comprehensive and coherent supportive supervision tools that encourage clear and evidence-based actions for improved quality of care.

## A53. The role of CHF-IMIS in health insurance scheme: a digital solution for enhancing penetration of health insurance coverage in Tanzania

### Nicholas S Kanisa^1^, Elizeus Rwezaura^1^, Ishengoma Josephat^1^, Peter Ngallya^2^

#### ^1^Health Promotion and Systems strengthening (HPSS) project, Dodoma, Tanzania; ^2^Presidents Office-Regional Administration and Local Government (PORALG), Dodoma, Tanzania

##### **Correspondence:** Nicholas S Kanisa (nicholas.kanisa@hpss.or.tz)

*BMC Proceedings 2023,* **17(13):** A53

**Introduction**

Community Health Fund (CHF) is the pre-payment insurance scheme which is operated by the Government of Tanzania as one of the health financing arrangements. To ensure smooth operationalization of CHF scheme, a web-based software (Insurance Management Information system-IMIS) was developed to enable members enrolment, client verification at a point of services, claims management and processing of payment to health facilities. The IMIS was developed 2013 by local Tanzania experts under the coordination of PORALG, MOH and NHIF with support from HPSS.

**Methodology**

The detailed analysis on IMIS functionalities, capacity and resources required to manage and maintain the system for smooth running health insurance. The analysis was based on system capacity to facilitate enrolment, renewal and health services utilization data. The capacity of IMIS as a web-based central database to handle real time data from villages, streets and health facilities overall the country.

**Results**

The IMIS has been able to meet the need and expectations of CHF as an insurance scheme. Currently, IMIS is in operational in public health facilities, some faith-based health facilities, 14,000+ villages, 2000+ mitaa in all 26 regions of Tanzania mainland. The system provides a very conducive environment for integrating with other health and financial systems. IMIS already integrated with GePG (E-payment), eGA (SMS), GoTHOMIS, AfyaCare and MUSE.

**Conclusion**

Through its integrated features including offline functionality, CHF-IMIS has made a huge revolution in Tanzania by enabling real-time members registration in all villages/mitaa regardless connectivity challenges in some remote-rural areas. At health facility, IMIS has simplifies the identification/client validation process and submission of services utilization data/claims. The system is imbedded with comprehensive formula for calculation of re-imbursement to health services providers; it has enhanced transparency and accountability of the fund. The implementation of CHF-IMIS has greatly enhanced the penetration/coverage of CHF Iliyoboreshwa in Tanzania mainland.

## A54. Leveraging digital health and ambulatory and taxi services to offer quality maternal and neonatal health services in the community

### Melania Nkaka^1^, Kenneth Ogendo^1^, Neema Kafwimi^1^

#### ^1^D-tree International, Dar es salaam, Tanzania

##### **Correspondence:** Melania Nkaka (mnkaka@d-tree.org)

*BMC Proceedings 2023,* **17(13):** A54

**Background**

The rates of maternal and infant mortality and morbidity are high in Tanzania at 578/100,000 and 32/1,000 live births respectively. Long distances from the households to health facilities coupled together with the lack of effective emergency transport systems contribute to high mortality rates. To address this, in 2015, D-tree, co-designed and implemented an Emergency Transport System using digital technology to link pregnant and postpartum mothers and newborns to health facilities.

**Methodology**

Dispatchers at the call centre in Shinyanga and Mwanza regions receive calls from community members or health facilities about cases that need immediate transportation. Supported by a mobile decision-support app, dispatchers triage calls to determine emergencies and referral locations; organise transport using government ambulances or community drivers; and follow up on referrals to obtain outcome data. Upon successful transportation, the system triggers payment to the driver through M-Pesa.

**Results**

Through the digital system, over 10k neonate and maternal emergencies have received transport services to appropriate levels of care. Early analysis showed the great impact this innovative solution has on reducing maternal and neonatal mortality by addressing the delays in reaching and receiving adequate care, as demonstrated by the outcomes whereby there was a 27% decrease in maternal mortality at a cost of $52 per life-year saved. The project was first launched in Sengerema and Buchosa District Councils in Mwanza region and was eventually scaled to the entire Shinyanga region in 2018 through collaboration with the DHMTs.

**Conclusion**

The results show that the use of the digital system can strengthen the primary healthcare system, and improve the quality of services for RMNCAH clients. Currently the Ministry of Health has adopted this model and is scaling it across the regions in Tanzania.

## A55. Community-based family planning approaches for first-time mothers: Results and lessons from small-scale testing

### Lilian Kapinga^3^, Melanie Yahner^2^, Emma Cook^1^, Meroji Sebany^2^, Jennifer Seager^1^, Brenda Mshiu^3^

#### ^1^George Washington University, Washington, United States of America; ^2^Save the Children US, Washington, United States of America; ^3^Save the Children International, Dar es salaam, Tanzania

##### **Correspondence:** Lilian Kapinga (Lilian.Kapinga@savethechildren.org)

*BMC Proceedings 2023,* **17(13):** A55

**Introduction**

Unintended pregnancy and short birth intervals among adolescents and young women are associated with increased risk of adverse perinatal outcomes. In many contexts, young mothers are less likely than older women to access health services, including postpartum family planning (PPFP). Reaching young mothers, particularly first-time mothers (FTMs) aged 15-24, during the transition to parenthood can improve service utilization, and support healthy timing and spacing of pregnancies.

**Methodology**

Connect project tested an integrated community-based approach to improve FTMs’ PPFP use in one district of Dodoma Region. The project supported community health workers (CHWs) to recruit FTMs into community support groups (CSGs) for pregnant and breastfeeding mothers, conduct home visits to FTMs, delivering integrated PPFP/nutrition counseling, referrals, and short-acting PPFP methods. A small-scale testing, involved two-round quantitative survey of 351 FTMs, discussions with 62 FTMs, 25 older female relatives, and 20 CHWs to assess effectiveness and inform scale-up.

**Results**

Findings showed that most FTMs attended 2-4 CSG sessions, and the majority (75.2%) received CHW home visit. PPFP adoption significantly Findings showed that most FTMs attended 2-4 CSG sessions, and the majority (75.2%) received CHW home visit. PPFP adoption significantly increased over time by 48%. Implementation learning showed that regular attendance of CSGs was a challenge due to limited incentives and competing priorities. Yet, many FTMs appreciated CSGs and home-visits by CHWs, which led to their PPFP adoption. Family members had positive attitudes towards CHWs, and supported FTMs in seeking PPFP services. However, reach of FTMs by our interventions is lower than expected during the small-scale testing, raising concerns about scalability and sustainability.

**Implication**

It was concluded that integrated community-based approaches targeting young FTMs have a positive impact on PPFP uptake. There is a clear need for advocacy to improve resourcing for CHWs for conducting home-visits, given the acceptability and promise of effectiveness for FTM PPFP uptake.

## A56. Increasing speed while maintaining quality: Lessons learned in adapting adolescent nutrition social and behavior change communication materials from Ethiopia to Tanzania

### Victoria Marijani^1^, Alex Rutto^1^, Joyceline Kaganda^1^, Carolyn O’Donnell^1^, Lydia Clemmons^1^

#### ^1^Save the Children International, Dar es salaam, Tanzania

##### **Correspondence:** Victoria Marijani (Victoria.Marijani@savethechildren.org)

*BMC Proceedings 2023,* **17(13):** A56

**Background**

In Tanzania, where 14-18% of adolescents experience malnutrition (TDHS 2010, 2015), the evidence base on adolescent nutrition (AN) has been limited and few nutrition social and behavior change communication (SBCC) materials for adolescents exist. In 2019, Lishe Endelevu, a five-year activity funded by the US Agency for International Development (USAID), faced the challenge of ensuring the rapid implementation of nutrition SBCC programming to improve nutrition outcomes among adolescents in four regions of Tanzania.

**Case Report**

The Activity made the strategic decision to adapt evidence-based AN SBCC materials from Ethiopia. Key adaptation steps included: 1. Review the evidence base on adolescent nutrition in Tanzania and Ethiopia. 2. Procure copies of AN SBCC materials from Ethiopia and adapt them to the Tanzanian context 3. Pretest the adapted materials with adolescent girls and their mothers, fathers and teachers. 4. Use the pretest findings to make further revisions to adjust to local Tanzanian context. 5. Submit the materials and pretest report to government authorities for technical review. 6. Incorporate technical review feedback to finalize the materials. 7. Reproduce and disseminate the materials. 8. Train teachers and community volunteers to use the materials. 9. Monitor implementation and document lessons learned. In less than 2 years from adapting the set of 8 adolescent nutrition SBCC materials, Lishe Endelevu reached 625 teachers and 3952 community health workers who in turn reached 100,370 adolescents through 493 in-school nutrition clubs and an additional 72,410 adolescents in out of school activities.

**Conclusion**

Programs can speed up implementation by obtaining existing evidence-based SBCC materials. Pretesting materials with the intended audiences, soliciting reviews and feedback from technical experts, and ensuring careful revisions are critical steps in process to adapt materials from other programs.

## A57. Relationship of maternal age and adherence to continuum utilization of maternal health services among pregnant women enrolled in the MomCare project

### Anunsiatha Peter Mrema^1^, Liberatha Shija^1^

#### ^1^PharmAccess International, Dar es salaam, Tanzania

##### **Correspondence:** Anunsiatha Peter Mrema (a.mrema@pharmaccess.or.tz)

*BMC Proceedings 2023,* **17(13):** A57

**Introduction**

Pregnant women in sub-Saharan Africa have low adherence to antenatal, facility delivery, and postnatal care regimens, contributing to high maternal, neonate, infant, and child mortality rates. Utilizing maternal health care services can reduce a substantial proportion of maternal mortality. The purpose of this study was to determine the relationship between maternal age and adherence to continuum utilization of maternal services among MomCare project clients.

**Methodology**

Data were derived from a MomCare Dashboard for the period between March 2019 and June 2022. Young pregnant mothers aged 19yrs and below and those aged 20 yrs. and above were included. Analysis of antenatal care, facility delivery, and postnatal care adherence was done, for each age group and presented as the frequency of visits attended.

**Results**

A total of 24,699 mothers were surveyed, among these 3489 (14%) were aged 19 yrs. and below and 21,210 (86%) were aged 20yrs and above. Of the 19yrs and below. 335(10%) start ANC at < 12 weeks of pregnancy, 1,634(47%) attained 4 + ANC visits, 1011(29%) delivered at a health facility, and had an average of 2.4 PNC visits. For mothers aged 20yrs and above 2585(12%) start ANC at < 12 weeks, 12,641(60%) attained 4+ ANC visits, 7,874(37%) delivered at health facilities, and had an average of 2.7 PNC visits. For mothers with 19yrs and below.

**Implication**

Results show that pregnant women aged 19yrs and below had lower adherence to maternal health services than pregnant women aged 20yrs and above. There is a need to assess factors that hinder adherence to care and to find ways that would help to improve adherence to continuum utilization of maternal health services.

## A58. Effectiveness of onsite mentorship on the uptake of family planning services: a lesson from Tanga city council, Tanzania.

### Mary Joseph^1^, Ester R Kimweri^2^, Msira Mageni^1^, Charles Mushi^1^,

#### ^1^JHPIEGO (Implementing Partner), Dar es salaam, Tanzania; ^2^Tanga City Council (Implementer), Tanga, Tanzania

##### **Correspondence:** Mary Joseph (Mary.Joseph@jhpiego.org)

*BMC Proceedings 2023,* **17(13):** A58

**Introduction**

Access to quality family planning services remains of paramount importance to Women in Reproductive Age (WRA) 15- 49 years by making an informed reproductive health choice. Globally, the technical skill and knowledge of providers on Family planning pose a challenge in the provision of a wide range of FP services (Farmer et al, 2015). It has been identified that the poor technical capacity of a health provider is a barrier to the provision of quality family planning services (Muttreja & Singh, 2018). Moreover, the landscaping assessment conducted in Tanga City before the implementation of the Challenge Initiative (TCI) project, revealed that, out of 129 providers, only 19% (24) were competent in the provision of Long-Acting Reversible contraception (LARC) services. This posed a missed opportunity on the uptake of family planning services. Weaver et al (2014) evidence shows onsite mentorship is effective in improving and maintaining performance among health care providers compared to classroom-based learning.

**Methodology**

Mentors were assessed on skills and technical capacity on family planning mentorship using a mentorship logbook then selection of mentees followed up before the process of mentorship. The role of the FP Coordinator was to select the facilities where mentorship would be conducted. From July 2019 to December 2021, a total of five (5) mentors were engaged by the program to provide mentorship in 20 TCI supported facilities in Tanga city. This programmatic practice includes both process and outcome approaches.

**Results**

A total of 47 health care providers from 20 health facilities of Tanga city council were mentored on counseling and provision of modern contraceptive methods as part of program implementation. To date, Tanga City has recorded an increased number of health care providers competent in the provision of modern contraceptive services from 19 to 66 (247%) in all 20 TCI-supported facilities in Tanga City. 22,941 Family Planning clients were recorded as Long-Acting Reversible Contraceptives users by July 2019, and 30,425 by December 2021. This reveals that, LARC uptake has increased by 32.6% from July 2019 to December 2021.

**Implication**

Mentorship enables the learner to practice their clinical skills and become more competent. The use of innovative strategies like WhatsApp groups and “Sisi Kwa Sisi” (Swahili term loosely translated “from us by us”) in mentorship enabled imparting knowledge and skills in the workplace. There was an increase in annual client volume and LARC uptake among women of reproductive age.

## A59. Labour market uptake of pharmaceutical dispensers in Tanzania- expectation and reality

### Romuald Mbwasi^2^, Karin Anne Wiedenmayer^1^, Kelvin Msovela^2^, Denis Mbepera^3^, Fiona Chilunda^4^, Sia Tesha^4^

#### ^1^Swiss Tropical and Public Health Institute,University of Basel, Switzerland; ^2^St. John’s University of Tanzania, Dodoma, Tanzania; ^3^Ministry of Health, Dodoma, Tanzania; ^4^Health Promotion and System Strengthening project, Dodoma, Tanzania

##### **Correspondence:** Karin Anne Wiedenmayer (karin.wiedenmayer@swisstph.ch)

*BMC Proceedings 2023,* **17(13):** A59

**Background**

Quality of health services is linked to medicines and related pharmaceutical services, while availability of trained pharmaceutical cadres is a prerogative. Considering the renewed focus on primary health care (PHC) and the human resource for health crisis affecting rural facilities, it is crucial to facilitate employment choices and opportunities for lower cadre pharmaceutical staff. Pharmaceutical Dispensers (PharmDisps) graduate after a one-year course. The aim of the study was to assess their labour market opportunities and uptake.

**Methodology**

The exploratory cross-sectional study was conducted in five regions (Dar Es Salaam, Morogoro, Dodoma, Shinyanga and Mwanza), using direct or face-to-face interviews. Questionnaires with open and close-ended questions were used to interview 207 and 31 employed and unemployed PharmDisps respectively, 28 employers, 6 tutors and 1 Pharmacy Council official. Data was electronically collected using the ODK application on tablets.

**Results**

There is high demand for PharmDisps, mostly at pharmacies and ADDOs. The reasons being staff shortage, the need for quality services and low salaries for this cadre. Salaries varied from 100’000 to 400’000 Tshs. 86% of the employed dispensers would like to go for further training. Perceived benefits for PharmDisps graduates are improved labour opportunity and social economic status. The majority of PharmDisps work in Dar es Salaam (28.5%) and Dodoma (38%). Activities include dispensing and store keeping. Tasks sometimes involve services beyond their competence without direct supervision, a problem for quality and safety. Securing a position was through friends (76.2%), by application and walk- in asking for a job. Half of graduates fail to get formal employment due to a change in regulation, refusing certificates when exited after one year. Both employers and dispensers acknowledged some female gender preference.

**Conclusion**

Expectations after graduation are high, hoping to secure jobs, to advance and be self-employed. The one-year dispenser course motivates to proceed to further training. There is an important market for PharmDisps, however regulatory recognition is an obstacle and hiring is not transparent; the workforce is poorly guided and monitored. Relevant authorities need to analyse this situation in view of the shortage of pharmaceutical staff for PHC. PharmDisps are needed and with supervision by higher cadres will improve the quality of pharmaceutical services in the country.

## A60. Dangerous traditional practice myth among urban-rural communities in the northwestern region of Tanzania

### Marko Hingi^1^, Jamal Magoti^2^, Julienne de Vastey^2^, Augustine Mhanga^3^

#### ^1^Tanzania Rural Health Movement, Mwanza, Tanzania; ^2^ Washington and Lee University ’23, United States, Washington, United States of America; ^3^Bisou Bailey Medical Dispensary, Mwanza, Tanzania

##### **Correspondence:** Marko Hingi (markohingi@gmail.com)

*BMC Proceedings 2023,* **17(13):** A60

**Background**

Traditional Uvulectomy is among of the dangerous myth existing in the communities across Tanzania despite the Acts governing traditional medicine practices restrict dangerous practices such as traditional uvulectomies. The communities seek for this myth due to various reasons including failure of babies to thrive, chronic cough among babies and sometimes they submit babies to this act on belief that it will prevent babies for childhood illness.

**Methodology**

The students from Washington and Lee University (USA) in collaboration with Tanzania Rural Health Movement developed a project entitled Save Life: Stop Traditional Uvulectomy and endorsed by District Medical Officer-Ilemela aiming to raise community awareness, and fortifying the existing emergency first responder system, with the ultimate goal of discouraging parents from subjecting their children to traditional uvulectomies and allowing faster and more efficient access to health care for the children who may undergo a traditional uvulectomy in Mwanza region.

**Results**

The project reached 240 participants (208 postnatal mothers, 13 community first responders, 19 health workers). Through simple survey the following data were extracted 18% (42/240) of participants their babies underwent traditional uvulectomy and chronic cough was leading cause by 30%, still the communities believe in traditional uvulectomy as best practice by 6% among the study population while 14% who their babies underwent traditional uvulectomy would not recommend such practice to other people.

**Conclusion**

There is urgent need to continue with advocacy on traditional uvulectomies and to involve men in the communities who have high power and influence on traditional uvulectomies, these efforts will accelerate towards achievement of Sustainable Development Goals target number 3.2 which states that by 2030.

## A61. Assessment of levels of quality antenatal care received by women with hypertensive disorders at Muhimbili National Hospital

### Isaya Erasto Mhando^1^, Furaha August^2^, Charles Kilewo^2^

#### ^1^St Joseph College of Health and Allied Sciences, Dar es salaam, Tanzania; ^2^Muhimbili University of Health and Allied Sciences, Dar es salaam, Tanzania

##### **Correspondence:** Isaya Erasto Mhando (isayaerasto@gmail.com)

*BMC Proceedings 2023,* **17(13):** A61

**Background**

Hypertensive disorders in pregnancy contribute to the direct causes of maternal mortality. The prevalence is 5-10% worldwide; and varies geographically. The burden of hypertensive disorders is higher in most clinical settings worldwide and sub-Saharan countries. Despite of good services provided at Muhimbili National hospital, there’s still large number of patients admitted already developed complications. This study aimed at assessing the levels of quality antenatal care received at antenatal level.

**Methodology**

Descriptive hospital based cross section study. The study was organized in two forms; quantitative and qualitative studies which was conducted from November 1st 2017 to 31st January 2018, 351 women were recruited, 20 women were selected for the in depth interview.

Information regarding counseling of danger sign, checking urine for protein, number of visits, blood pressure measurements on every visit and exploring client’s views on the care received, was collected from the Antenatal cards.

**Results**

The levels of quality antenatal care were 37%, 26% and 37 % for good, adequate and poor quality respectively. The proportion of women counseled for danger signs was (264) 75.2%, women who had urine for protein and blood pressure checked on every visit were89 (25.4%) and 307 (87.5%) respectively. women achieved ≥4visits were 184(52.4%).

Qualitative interview explored 3 themes;”lack of feedback on blood pressure and urine test”, “poor prioritization of visits in high risk mothers” and, “good counseling practice”, however among patients who had eclampsia,68% fitted at home compared to 10.3% who fitted at Muhimbili National hospital.

**Conclusion**

Most women admitted at MNH due to hypertensive related complications of pregnancy are already in severe forms due to poor quality of care received at antenatal level. Regular supervision ,supply of Valid bp-equipments ,and Antenatal staff- inservice training of the complications of hypertension in pregnancy are recommended.

## A62. Point-of-care testing for sexually transmitted infections with molecular diagnosis as way forward in Mbeya-Tanzania

### Anifrid Mahenge^1^, Ruby Mcharo^1^, Regino Mgaya^1^,Christof Geldmacher^2^, Jonathan Mnkai^1^, Beatrice Komba^1^, Wolfram Mwalongo^1^, Ruby Mcharo^1^

#### ^1^NIMR Mbeya Medical Research Center, Mbeya, Tanzania; ^2^Division of Infectious Diseases and Tropical Medicine, University Hospital of Munich partner site, LMU, Munich, Germany

##### **Correspondence:** Anifrid Mahenge (amahenge@nimr-mmrc.or.tz)

*BMC Proceedings 2023,* **17(13):** A62

**Introduction**

The global prevalence and incidence of Sexually Transmitted Infections (STIs dramatically rises. Most of STIs intervened syndromically with rely on conventional assay as routine among African countries Point-of-care molecular diagnostics is beneficial for STIs detection in resource-limited settings. Herein we report the performance of POC-STI using a self-collected cytobrush and urine from young adult females in Mbeya, Tanzania to update the prevention strategy.

**Methodology**

Prior STI study initiation on April 2020 we tested twenty two cytobrush and urine samples with GeneXpert as standard against multiplex CFX96 system. All specimens were randomly selected from data base whereby fresh urine were tested with GeneXpert using STI cartilage; Again urine and cytobrush were used for DNA extraction and detection with 7-essential STIs assay using multiplex CFX96 system. Sensitivity and specificity was used to estimate test accuracy at 95% confidence intervals (CIs).

**Results**

Our report has found that, the sensitivity of GeneXpert against CFX96 for diagnosis of C.trachomatis, N.gonorrhoea and T.vaginalis using urine was 82.33% confidence interval (CI) 35.88% to 99.58% 35.88% to 99.58%. Along with, specificity of it in the same sample type was 100.00% CI 78.20% to 100.00%.Moreover, we compared the same participant using urine and cytobrush, of note the sensitivity was 83.33% CI 33.1-99.6% and specificity 92.87% CI 66.13-99.8%.Therefore, both sample types has more than 90% accuracy in all instruments.

**Conclusion**

Briefly, this report verified high performance of POC-STIs from self-collected cytobrush followed by urine samples. Molecular diagnosis of STIs offers quality and reliable results so as to assist more screening and diagnosis of missed STIs with conventional test to reduce the burden of STIs in general population.

## A63. Retrograde intrarenal surgery with flexible ureteroscopy and laser lithotripsy for kidney stones. The Aga Khan hospital Dar Es Salaam experience

### Ali A. Zehri^1^, Hussam Uddin^1^

#### ^1^Aga Khan Hospital in Dar es salaam, Dar es salaam, Tanzania

##### **Correspondence:** Ali A. Zehri

*BMC Proceedings 2023,* **17(13):** A63

**Background**

Retrograde intrarenal surgery (RIRS) with laser lithotripsy to a large extent gained popularity worldwide due to significant improvements in technology and techniques, currently considered as one of the best options in management of Urolithiasis.

**Objectives**

The aim of this study was to evaluate the efficacy and safety of retrograde intrarenal surgery with flexible ureteroscopy and laser lithotripsy (RIRS) for the treatment of renal stones and to analyze the predictive factors for stone-free rate.

**Materials and Methods**

We retrospectively reviewed the records of patients who underwent retrograde intrarenal surgery with flexible ureteroscopy and laser lithotripsy for renal stones from February 2019 to July 2022. We identified 76 patients’ data from flexible ureteroscopy with laser lithotripsy registry. Stone-free and success were respectively defined as no visible stones, clinically insignificant residual stones less than 3 mm on postoperative imaging; predictive factors for stone-free were evaluated.

**Results**

A total of 76 procedures performed in 71 patients including 5 bilateral renal units. There were 52 male and 24 female patients; Computed Tomography was the man radiological imaging in 95.74 % of patients. The anatomical distribution of stones according to renal calyceal system, 11 patient stones (14.47%) were located in the upper pole, 14 stones (18.42%) in midpole, 9 stones (11.84%) in renal pelvis and 42 stones (55.26%) in the lower pole respectively. The mean cumulative stone burden was 14.9 ± 4.23mm. The postoperative stone-free rate was 92.5 % at 1 month after surgery. The overall complications 17%, majority were (Clavien I/II, 16% and Clavien III, 1%).

**Conclusions**

Retrograde intrarenal surgery with Flexible ureteroscopy and Laser lithotripsy is a safe and effective minimally invasive option for renal stones of 2 cm or less and gaining popularity in bigger stone with staged procedures.

## A64. Applying a stepwise and coordinated approach towards national covid-19 vaccination goals. data as a key enabler for Tanzania

### Harrison Mariki^1^, Mwendwa Mwenesi (MD)^1^, Florian Tinuga (MD)^1^, Kokuhabwa Mukurasi (MD)^1^

#### ^1^Afya Intelligence, Dar es salaam, Tanzania

##### **Correspondence:** Harrison Mariki (hmariki@afyaintelligence.co.tz)

*BMC Proceedings 2023,* **17(13):** A64

**Introduction**

COVID-19 vaccines have proven to be the safest intervention in protecting the population against SAR-CoV2 infections, hospitalization, severe diseases, and death. Although the country had access to the COVID-19 vaccine in July 2021 through COVAX Facility and donations, only 10.6% of the eligible population (Adults aged 18years and above) was fully vaccinated as of 31st March 2022. This proportion was far less than the WHO global targets on COVID-19 vaccination. To ensure increased uptake of the COVID-19 vaccines and achieve the global targets, Tanzania introduced extended campaigns conducted in stepwise approach, utilizing community-based and house to house campaigns. Phase one the campaign targeted to fully vaccinate 30% of the eligible population by June 2022. Phase two and three of the extended campaign targeted to fully vaccinate 50% and 70% of the eligible population by September 2022 and December 2022 respectively.

**Methodology**

Tanzania is leveraging data science for a predictive utilization-focused monitoring, evaluation and adaptive learning (MEAL) approach to address the unique challenges in providing community-based COVID-19 vaccination services such as shortage of vaccines, resource constraints, sociocultural factors, dynamic populations, and geographic barriers. Our methodology involves collecting daily performance data for a minimum set of data elements (vaccination by gender, type,dose, and location) jointly agreed by stakeholders as of high importance to the community based vaccination efforts. The Immunization and Vaccines Development (IVD) program, in partnership with Afya Intelligence then conducts rigorous data analysis mapping any inequities to generate foresights on how the program and regions must adapt and adjust to fit unique contexts. Data is then disseminated through a facilitated process in weekly vaccine pillar meetings, where partners discuss the findings to inform the modifications of the vaccination strategies.

**Results**

This unique MEAL approach has helped inform motivation, supervision and support for low performing regions and contributed to adaptable community-based vaccination campaigns. As of 31st July 2022, during the dissemination of week 12 findings 50% of the regions had reached the goal of phase 1 (30% of the eligible population fully vaccinated). As of 19th September 2022, 65% of the regions have already achieved phase 2 goals and the IVD program has adopted a modified goal of 100% full vaccination of the eligible population by December 2022.

**Conclusion**

Leveraging data for predictive and focused MEAL is particularly relevant and effective to adapt COVID-19 vaccination efforts and helps to build responsive and resilient vaccination programs. Stakeholders have benefited from weekly adaptations suggested to ensure the set stepwise goals are reached.

## A65. Adoption of a public-private partnership in Tanzania - the case of Jazia prime vendor system

### Denis Mbepera^2^, Romuald Mbwasi^1^, William Mfuko^3^, Fiona Chilunda^4^, Manfred Stoermer^5,6^ and Karin Wiedenmayer^5,6^

#### ^1^St. John’s University of Tanzania, Dodoma, Tanzania; ^2^Ministry of Health, Dodoma, Tanzania; ^3^Senior pharmaceutical consultant, Dar es Salaam, Tanzania; ^4^Health Promotion and System Strengthening (HPSS) Project, Dodoma, Tanzania; ^5^Swiss Tropical and Public Health Institute, Basel, Switzerland; ^6^University of Basel, Basel, Switzerland

##### **Correspondence:** Karin Wiedenmayer (Karin.wiedenmayer@swisstph.ch)

*BMC Proceedings 2023,* **17(13):** A65

**Background and objectives**

Jazia Prime Vendor System (Jazia PVS) is a Public Private Partnership (PPP) for procuring health commodities when not available at Medical Stores Department (MSD). The system is operating in Tanzania since 2018. The Government recognizes the role of the private sector in bringing about socio-economic development. PPPs can effectively address constraints of financing, management and maintenance of public goods and services. This study showcases the successful adoption of a PPP to bridge the supply gap in health facilities, contributing to improve health care services.

**Methodology**

Regional procurement volumes from contracted private suppliers were compiled from 2015 starting when covering 3 regions to 2021 when covering 26 regions. Data collection used standard monitoring checklists and secondary information from 26 (100%) regions and their respective contracted regional vendors. Analysis was conducted using Microsoft office excel.

**Results**

Since implementation of PVS, the volume of health supplies procured from private vendors increased from Tsh 1,633,071,570 in 2015 to Tsh 10’206’723’555 in 2021. Procurement was highest in 2019 and decreased in 2020 (pandemic), while increasing again in 2021 from 53% to 92%. Increase of complementary procurement from the private sector due to shortages at MSD over the years points to determinants such as external influences of population growth, epidemiological transition and disrupted supply chains during the pandemic as well as MSD internal challenges of fiscal capitalization and management.

**Conclusion**

The impressive adoption of the Jazia PVS demonstrates the feasibility and effectiveness of a PPP in health supply chain management. The adoption growth of the PVS procurement volume also validates the need for complementary supply chain models in dynamic health systems. A solid policy basis, transparency and efficiency of procurement procedures and contractual adherence from both public and private partners are prerequisites for a successful PPP in the health sector, promoting confidence in the Tanzanian health care system.

## A66. Expanding access of supply chain data through common digital communication platfoms - a successful application of artificial intelligence for healthcare in Tanzania

### Franc Mussa Israel^1^, Elizabeth Herman^2^Pachal Thomas Kija^4^Bernadette Masawe^3^ Sadick Masomhe^5^Chinaa Swai^2^ Felists Makene^5^

#### ^1^Afya Intelligence.co.tz, Muhimbili university of health and allied science, Dar es salaam, Tanzania; ^2^Afya Intelligence Company L.t.d, Dar es salaam, Tanzania; ^3^Muhimbili university of health and allied science^4^University of Dar es Salaam^5^Sahara Ventures, Dar es Salaam, Tanzania

##### **Correspondence:** Franc Mussa Israel (franc@afyaintelligence.co.tz)

*BMC Proceedings 2023,* **17(13):** A66

**Background**

Afya Intelligence leveraged existing platforms, networks and workflows by testing the integration of common social messaging platforms (WhatsApp and Telegram) with the national Electronic Logistics Management Information System (eLMIS). Afya Intelligence aimed to send out-of-stock notifications to healthcare providers' mobile phones directly and enable supplemental fulfilment from the private sector whenever necessary. This was done through the UNFPA funded SRH Data hack AMUA program. With this integration, text message notifications for stock out are made possible.

**Methodology**

The AMUA program was able to confirm that once the MSD out of stock notification is made available by eLMIS, the health facility contact person was notified immediately through WhatsApp. The health facility is able to download the document with all the information needed to proceed to source commodities from prime vendor. The findings indicate that applying Afya Intelligence can reduce the time for notification from 30 days to 1 day yielding several benefits in the healthcare supply chain including: stock out reduction, supporting complex decision-making process and simplifying procurement process.

**Results**

Achieving successful outcomes for people and the community is less likely when health supply networks are underperforming. The Tanzanian government is using the Prime Vendor model, which enlists private sector pharmaceutical suppliers to provide supplemental medications needed by public health facilities, in order to increase product availability when the Medical Stores Department (MSD) is out of stock. For this to be successful, stakeholders from all levels and industries must have access to current information

**Conclusion**

For healthcare programs to succeed, diverse health care stakeholders, including frontline health workers, managers, policymakers, suppliers and everyone in between, need the means to get timely feedback on where further improvements are needed. Integrating eLMIS with social messaging technology simplifies feedback between digital systems and diverse supply chain stakeholders.

## A67. The power of agyw groups in controlling new HIV infections

### Kawogo Malisela^1^, Sasya Hashim^1^, Maro Amani^1^, Ngowa Mary^1^, Makyao Neema^1^, Nyirenda Amos^1^, Ngaleson Frida^1^

#### ^1^Amref Health Africa, Dar es salaam, Tanzania

##### **Correspondence:** Kawogo Malisela (malisela.kawogo@amref.org)

*BMC Proceedings 2023,* **17(13):** A67

**Introduction**

Tanzania HIV Impact Survey 2016/2017 indicates that Adolescents and Youth account for 40% of all new HIV infections. Adolescent girls and young women (AGYW) accounts for 80% of HIV new infection among youth. The AGYW program branded as ‘Timiza Malengo Program’ is part of the GF program implemented in five regions of Morogoro, Dodoma, Tanga, Geita and Singida that aims at reducing new HIV infections among AGYW.

**Methodology**

Timiza Malengo Program targets in and out of school AGYW aged 10 – 24 years from TASAF and Non TASAF vulnerable households. It delivers age-appropriate & client-centered comprehensive combination prevention interventions to reduce new HIV infections. It employs group model to reach AGYW with defined package of services (DPS) and track the layering with enhanced referrals and linkages across services delivery points. AGYW form small groups of 5 – 10 members for peer led sessions as part of DPS and linkages to other behavioral, biomedical and structural interventions.

**Results**

A total of 104,870 (107%) AGYW were reached with defined package of services (DPS) through the use of formed groups. A total of 97,389 (97%) were tested for HIV and received test results. The formed 2,248 AGYW groups facilitated the program to reduce new HIV infections among enrolled AGYWs through peer led sessions, condom provision, STIs screening and referrals, and Income Generating Activities. We also ensured tracking completeness of services and enhancing referrals and linkages across service delivery points. The group formation included registration of groups to access council loans opportunities, bank services and other relevant opportunities.

**Implication**

Timiza Malengo uses group formation to deliver comprehensive package of services to AGYW. The use of groups, enhanced referrals and linkages across different service delivery points have proven to be best approach in reaching AGYWs for HIV combination prevention services including prevention of new HIV infections.

## A68. Mortality rate and predictors of mortality among covid-19 patients at KCMC hospital in northern Tanzania

### Gilbert Godfrey Waria^1^, Florida J. Muro^1,2^, Kajiru G. Kilonzo^1,2^, Laura Shirima^1^, Michael Ngowi^1^, Norman Jonas^1,2^, Henry L. Mlay^1^, Innocent B. Mboya^1^

#### ^1^Kilimanjaro Christian Medical University College (KCMUCo), Moshi, Tanzania; ^2^Kilimanjaro Christian Medical Centre (KCMC), Moshi, Tanzania

##### **Correspondence:** Gilbert Godfrey Waria (waria751@gmail.com)

*BMC Proceedings 2023,* **17(13):** A68

**Introduction**

A total of 547 confirmed COVID-19 patient records were included in the study. Their median age was 63 (IQR; 53-83), about 60% were aged 60 years and above, and 56.7% were males. The most common clinical features were; fever 60.8%, severe form of disease 44.4%, difficulty in breathing 73.3%, chest pain 46.1%, generalized body weakness 390 (71.3%). Of all participants, over one-third (34.6%) died (95%CI; 0.31-0.39). The median survival time was 7 days (IQR;3-12). The overall mortality rate was 32.33 per 1000 person-day. The independent predictors associated with higher hazard of mortality were age ≥60 years (AHR=2.01; 95%CI 1.41-2.87; P<0.001),disease severity (AHR=4.44; 95%CI 2.56-7.73; P<0.001) , and Males (AHR=1.28; 95%CI; 0.93-1.73; P=0.128).

**Methodology**

This was the hospital-based retrospective cohort study, conducted at KCMC Hospital in Northern Tanzania among all admitted patients with confirmed COVID-19, from 10th March 2020 to 26th January 2022. The main study outcome (event) was death (Yes/No). The predictors of mortality were determined by using Weibull survival regression model. The significance level was set at p-value of <0.05.

**Results**

A total of 547 confirmed COVID-19 patient records were included in the study. Their median age was 63 (IQR; 53-83), about 60% were aged 60 years and above, and 56.7% were males. The most common clinical features were; fever 60.8%, severe form of disease 44.4%, difficulty in breathing 73.3%, chest pain 46.1%, generalized body weakness 390 (71.3%). Of all participants, over one-third (34.6%) died (95%CI; 0.31-0.39). The median survival time was 7 days (IQR;3-12). The overall mortality rate was 32.33 per 1000 person-day. The independent predictors associated with higher hazard of mortality were age ≥60 years (AHR=2.01; 95%CI 1.41-2.87; P<0.001),disease severity (AHR=4.44; 95%CI 2.56-7.73; P<0.001) , and Males (AHR=1.28; 95%CI; 0.93-1.73; P=0.128).

**Conclusion**

Mortality was higher in elderly male patients, with severe form of disease and those with any comorbidities. Therefore, more attention should be provided among older male patients including uptake of current vaccine and ensuring standard and supportive care at primary health facilities are available.

## A69. Spectrum of cardiac arrythmias and its influence on 30-day stroke mortality in Dar es Salaam

### Julius N. Msukuya^1,^ Ahmed Jusabani^2^, Robert Mvungi^3^, Philip B. Adebayo ^4^

#### ^1^Jakaya Kikwete Cardiac Institute, Muhimbili National Hospital, Dar es Salaam, Tanzania; ^2^Department of Radiology, Aga Khan Hospital, Dar es Salaam, Tanzania; ^3^Cardiology Section, Department of Internal Medicine, Aga Khan University and Aga Khan Hospital, Dar es Salaam, Tanzania; ^4^Neurology Section, Department of Internal Medicine, Aga Khan University and Aga Khan Hospital, Dar es Salaam, Tanzania

##### **Correspondence:** Julius N. Msukuya (jmsukuya@gmail.com)

*BMC Proceedings 2023,* **17(13):** A69

**Background:** Cardiac arrythmias are common after stroke due to the disruption of the brain-heart autonomic regulation. Cardiac arrythmias in stroke has not been fully evaluated in our settings.

**Aims and Objectives:** To profile the types of cardiac arrythmias in acute stroke patients admitted to the Muhimbili National Hospital (MNH) and the Aga Khan Hospital (AKH), Dar es Salaam. We determined the clinical correlates of cardiac arrythmia and the effect of cardiac arrythmias on 30 days stroke mortality among the cohort.

**Methods:** This was a prospective longitudinal observational study of consecutive stroke patients, 18-years and above admitted to the above hospitals between October 2019 and March,2020. Radiologically confirmed stroke patients were screened for cardiac arrhythmia using 12-lead ECG within 72 hours post stroke. 30 days case fatality was determined.

**Results:** Among 222 acute stroke patients admitted, significant cardiac arrhythmia occurred in 30 patients (13.96%) in the first 72 hours of acute stroke. Atrial fibrillation 10(5.4%), ventricular tachycardia 7(3.2%), sinus arrhythmia 7(3.2%), sinus bradyarrhythmia 2(0.9%), ventricular fibrillation 2(0.9%), premature ventricular complex 2(0.9%) and atrial flutter 1(0.5%) were identified in acute stroke. Cardiac arrhythmias were independently associated with high stroke severity score, haemorrhagic stroke and left cerebral hemisphere stroke. Cardiac arrhythmia was an independent predictor of poor outcome in acute stroke. 30-days case fatality was 27% among the cohort with cardiac arrythmias.

**Conclusion:** Cardiac arrhythmia are common in this cohort of acute stroke patients. Atrial fibrillation and ventricular tachycardia were the leading arrythmias. Presence of cardiac arrythmias contributes to poor stroke outcome.

## A70. Pattern for provision of post violence services among victimized women and children in Tanzania

### Queen-Ruth Msina ^1^

#### ^1^MSc Candidate International Public Health, Department of Global Health and Bioethics, Euclid University, Bangui, Central African Republic

##### **Correspondence:** Queen-Ruth Msina (qruthmsina@gmail.com)

*BMC Proceedings 2023,* **17(13):** A70

**Introduction**

Tanzania established the National Plan Against Violence Against Women and Children (NPA-VAWC) to provide response services for services for survivors and prevent new incidents. The rolled-out gender desks provide services such as psychosocial counselling, forensic investigation, Ultra pregnancy test, emergency contraceptives, Post Exposure Prophylaxis, antibiotics, police service and legal aid (MOHCDGHE, 2016).

**Objective**: This study identifies the trend of post-trauma services provision among victimized women and children in Kinondoni District.

**Methodology**

This is a retrospective study targeting women and children who have undergone violence in Kinondoni District between November 2020 and April 2021. With consent, secondary data was collected from the Data Management System of the Municipal Council. Primary data was collected via open-ended interviews with the Social Welfare Officer, and Data Management Officer. Data was entered, analyzed, and represented in pie charts and tables.

**Results**

Out of the 1,460 participants, 68% were women above 25 years with the least affected age group being children of age zero to four years which attribute 2%. The forms of violence in decreasing frequencies were: emotional, physical and sexual. Less than 40% of victimized women and children received comprehensive post violence services in a timely manner due to reasons such as unreliable availability of medications and medical supplies, underreporting of cases, weak referrals and cost involvement for legal aid. However, health services were given a priority in comparison to justice-related services for survivors.

**Conclusion**

Majority of the victims do not get timely and comprehensive services per national guidelines due to poor reporting system, lack of availability of medications at the facility, poor cooperation from the victims and supporting relatives/friends and legal service expenses.

Moreover, health services were given a priority in comparison to legal services for survivors due to cost involvement in legal services.

**Recommendations**

Improvement of referral linkages in governmental and non-governmental organizations, with more efforts placed in the legal system. More integrated programs for violence fighting against children and women so as to combat problem as a unit. Continuous training to students in medical and sociology schools; health care providers and social welfare officers on how to handle and refer the victims. Technology leverage to whistle blow incidences and improve post violence referrals.

## A71. The role of digital health for improving access to physiotherapy care to address musculoskeletal pain and disability

### Pendo Shayo^1^, Janet Bettger^3^, Colleen Burke^2^, Kelvin Haukila^2^, Katherine Norman, Mathew Shayo^1^

#### ^1^Kilimanjaro Christian Medical Centre, Moshi, Tanzania; ^2^DUKE University, Durham, North Carolina; ^3^Temple University, Pennsylvania, United States of America

##### **Correspondence:** Pendo Shayo (pendoshayo95@gmail.com)

*BMC Proceedings 2023,* **17(13):** A71

**Introduction**

Globally, low back pain (LBP) continues to be the leading cause of disability. Tanzania had a 36% increase in disabling LBP from 2007 to 2017, making it the second leading cause of disability. Although exercise therapy can decrease musculoskeletal (MSK)-related pain and activity limitations, access to physiotherapy is challenging.

**Methodology**

This study aimed to assess the feasibility of adapting evidence-based MSK interventions that integrate digital health to support adherence for home-based exercise therapy in Northern Tanzania.

We examined related literature and compared evidence-based care models that included digital health strategies to reach patients at home with current practice for Northern Tanzania. We engaged stakeholders to assess feasibility, acceptability, and appropriateness of adding text reminders and weekly telephone counseling for patients after a rehabilitation clinic visit. An interdisciplinary team designed a culturally and contextually appropriate approach to remote care delivery.

**Results**

Currently in Tanzania, digital health technology is not applied in expanding access to rehabilitation for MSK. Public data identified 75% of adults own a mobile phone and mobile access penetration (access without ownership) is much higher. Preliminary work found <5% of text messages were not delivered due to network or power issues. Clinicians felt that digital health was a good fit for the patient population and region. The 2021 Tanzanian National Rehabilitation Strategic Plan identified digital health as a key strategy for improving access. Cultural adaptation is needed to optimize text and telephonic assistance to support patient adherence at home.

**Conclusion**

A digital health strategy could feasibly improve access and adherence to exercise to reduce the burden of MSK pain and functional limitation among Tanzanians who are socioeconomically and geographically disadvantaged.

## A72. Knowledge and attitude toward trachoma among final year clinical medicine students in selected colleges of health and allied sciences in Dar Es Salaam

### Laurent E. Marishamu^1^, Milka Mafwiri^1^

#### ^1^Muhimbili University of Health and Allied sciences, Dar es salaam, Tanzania

##### **Correspondence:** Laurent E. Marishamu (mrimbotonica@gmail.com)

*BMC Proceedings 2023,* **17(13):** A72

**Background**

Trachoma is the commonest infectious cause of blindness worldwide, caused by Chlamydia trachomatis and remains a significant public health concern. Africa is the continent where the largest prevalence of trachoma is found. Tanzania is one of the countries of higher prevalence of trachoma and limited knowledge and attitude towards trachoma.

**Objective**: The aim was to assess knowledge and attitude toward trachoma among final year clinical medicine students.

**Methodology**

A descriptive cross-sectional study using quantitative techniques was conducted in three colleges. Simple random sampling techniques were used to select participants. Self-administered questionnaires were used as an instrument to assess the knowledge and attitude on trachoma. Data was entered by EPI DATA and analyzed by using SPSS version 20.0 software. Ethical clearance was obtained from IRB in MUHAS.

**Results**

This study was conducted in three colleges which are found in Dar es Salaam and a total of 384 participants met the inclusion criteria. Majority (73.9%) of them was aged between 18–24-year-old and (87.8%) were single. Majority of them, 69.9% of respondents were certificate in clinical medicine holders. Most of the participant (74.1%) were able to define trachoma and knew that trachoma is caused by bacteria. In this study, 98.7% believed that trachoma is a treatable disease. Also, 98.4% and 96.0% of the respondents believed that trachoma is a preventable disease/problem and face cleaning is good for preventing trachoma respectively. Furthermore, the ordinary level and certificate in clinical medicine holder were knowledgeable on prevention by 68% and 67.7% respectively. Moreover, 97.2% of participants who were aged 18-24 years had positive attitude trachoma followed by 96.6% of those aged 20-25

**Conclusion:**

In this study, despite the high favorable attitude toward trachoma infection, the proportion of students who had adequate knowledge about trachoma was low.

## A73. Men adjustment to the emotional and behavioral changes of their partners during pregnancy

### Zaituni Chegamila^1^, Golden Masika^1^

#### ^1^ University of Dodoma, Dodoma, Tanzania

##### **Correspondence:** Zaituni Chegamila (zaitunic77@gmail.com)

*BMC Proceedings 2023,* **17(13):** A73

**Background**

Pregnancy is a period accompanied by various emotional and behavioral changes that occurs as a result of a woman’s body adjusting to the physiological changes as fetus grows in her womb. Their male partners are impacted differently by these changes and impacts their adjustment pattern as coping mechanisms may have impact on the women livelihood and health during pregnancy period and childbearing. However, there is no study that that provide a clear theory on how to predict the male partners adjustment pattern to offer direction of interventions that can help couples and/ families to remain stable and supportive to each other during critical and precious period of pregnancy and childbirth.

**Methodology**

A grounded theory approach was used, utilizing data from 29 in-depth interviews involving men living with pregnant partners or those who lived with a pregnant partner.

**Results**

With a theoretical sample of 29 men, the findings of this study came up with three core categories which are presented into four phases showing how they are related to each other. The first phase is the perception of pregnancy, the second phase is how women pregnancy changes shaped men’s direction of adjustment, and the third phase is men’s initial adjustments to reconstruct the relationship and final phase is the adjustment phase.

**Conclusion**

Findings of this study will provide information that will pave the way for developing interventions that will bring male partners on board in supporting their wives/partners towards healthy and safe pregnancy and childbearing.

## A74. Improving pre-exposure prophylaxis uptake among key and vulnerable population in Kagera region

### Patrick Mwanahapa^1^, Aveline Minja^1^

#### ^1^Management and Development for health, Dar es salaam, Tanzania

##### **Correspondence:** Dr Patrick Mwanahapa (pmwanahapa@mdh.or.tz)

*BMC Proceedings 2023,* **17(13):** A74

**Introduction**

WHO recommends PrEP use among populations with a substantial risk of HIV/AIDS. Proven when used correctly and consistently reduces the risk of HIV infection during sex by 90%. Literature shows high HIV prevalence among KVPs (FSW: 15.3%, MSM: 8.3%, PWID: 8.7%) compared to the general population (4.7%.) across Tanzania. Ministry of Health recommends a facility-led community PrEP delivery model. However, from October to December 2021, KVPs who tested HIV negative were linked to Health facilities for PrEP services, 901 KVPs according to DHIS2 were initiated PrEP which is low compared to the estimated PrEP eligible clients (514,540 KVPs) within the Tanzania (IBBS 2014).

**Methodology**

PrEP Champions: are clients with experience and currently using PrEP, PrEP Champions were involved when offering PrEP Health Education sessions to improve awareness and uptake among eligible KVPs clients. Training on Quality and Stigma-Free Services; Helped to work effectively with KVPs for PrEP Services by having self-awareness about KVP-friendly services and practice services that are non-judgmental. Training on facility-led community outreach monitoring, increased community accountability, feedback and quality towards KVPs services.

**Results**

The number of KVP clients initiated on PrEP services increased from 901 to 2,359 (January to March 2022) based on DHIS2 database. Noticed further progressive increment 596 (January), 786 (February) and 977 (March) after engaging more PrEP Champions per month to create awareness and support enrollment of clients.

**Implication**

Some clients refused PrEP services due to pills burden and storage pack (tin) resembling those for HIV care posing stigma and discrimination to non-PrEP users. Ministry of Health to establish an alternative of oral PrEP drug like Long acting injectable cabotegravir or Dapivirine vaginal ring in order to reduce pills burden according to WHO recommendation. Implementing partners to ensure the use of facility-led community outreach service delivery modal to reduce stigma and improve the uptake of PrEP service.

## A75. Pre-exposure prophylaxis (prep) interruptions among key and priority populations, their associated reasons and recommendations to improve prep continuity in Tabora, Tanzania

### Adam Mrisho^1^, Honoratha F Rutatinisibwa^2^, David Sando^1^, Ramadhani Shemtandulo^1^, Steven Ambonisye^1^, Michael Mahande^1^, Benson Mturi^1^, Goodluck Lyatuu^1^, George Msalale^2^

#### ^1^Management and Development for Health (MDH), Dar es salaam, Tanzania; ^2^Regional Administrative Secretary, Health-Tabora, Tabora, Tanzania

##### **Correspondence:** Adam Mrisho (amrisho@mdh.or.tz)

*BMC Proceedings 2023,* **17(13):** A75

**Introduction**

In Tanzania, PrEP is a new prevention intervention, the progress in scaling up PrEP services is low among Key and Priority Populations (KPPs). In Tabora, PrEP continuity after 6 months among newly KPP started PrEP drug is low at 53%. However, information on barriers to PrEP interruptions in KPP in Tabora and Tanzania is limited. We report study findings from nine (9) health facilities (HFs) in the Tabora region.

**Methodology**

This was a cross-sectional survey that employed both quantitative and qualitative methods. In total, 425 current or previous PrEP beneficiaries were administered with a structured questionnaire, whereby 157 participants were purposively selected for 18 focused group discussions (FGDs). The quantitative data analysis was done using STATA 13.1, while qualitative data were translated, transcribed, coded and analysed.

**Results**

From a total of 425 KPPs, aged 15-70 years with average 30 years, the majority 288(58%) were FSWs, 110 (25.9%) were knowledgeable of PrEP. The proportion of interruption was 25 (20.5%). Reported reasons for PrEP interruptions, more than half (57%) were due to drug side effects and the least was migration (3%). Their recommendations themed emerged were strengthening PrEP awareness, PrEP drug accessibility, change of drug dosage and package, reducing drug side effects or reactions, and incentives. There was an association between interruptions and clients’ age (P<0.05), area of service offered (P=0.010.) and being knowledgeable about PrEP: (P<0.05).

**Conclusion**

Being knowledgeable about PrEP services plays a vital role to influence PrEP interruptions. Side effects dominate among reasons for interruptions. Recommended to conduct further study on the suggestions provided by PrEP clients to improve PrEP continuity.

## A76. Use of mobile van clinics in reaching tb missing cases in 8 regions in Tanzania

### Lilian T. Ishengoma^1^, Amos Nyirenda^2^, Hassani Mataka^1^, Emmanuel Matechi^1,3^, Frank Ntogwisangu^2^, Amani Maro^2^

#### ^1^ Management Development for Health, Dar es salaam, Tanzania; ^2^ Amref Health Africa, Dar es salaam, Tanzania; ^3^ National TB and Leprosy Programme, Dar es salaam, Tanzania

##### **Correspondence:** Lilian T. Ishengoma (Lilian.Ishengoma@Amref.org)

*BMC Proceedings 2023,* **17(13):** A76

**Introduction**

Tanzania is among the 30 high burden countries with TB with 222/100,000 incidence and 64% treatment coverage (WHO,2020). In September 2021, the county Launched Mobile-Van clinics for TB services to find TB missing people as one of early TB case detection strategies.

**Methodology**

Mobile-Van Clinics were placed for at least two days in hard to reach areas to find TB missing cases in 64 councils of 8 regions (Tanga, Simiyu, Mara, Dodoma, Shinyanga, Mbeya, Ruvuma and Kagera). Community leaders and public announcements for community sensitization were done prior the event. Community Health workers and Health care workers from nearby Health facilities together with Mobile-Van staff were providing onsite TB education, TB screening, TB diagnosis and treatment in case of TB detection.

**Results**

In a period of four months, 13,524 people were reached, among them 10,718 were screed for TB and 6,268 presumptive cases were identified of which 5,207 clients were examined through X-ray and 1,612 were TB suggestive. Despite the fact that GeneXpert is the first-line TB diagnostic service, only 2,162 clients (34.5%) were examined with GeneXpert and 55 TB cases (3%) were detected making a total of 1,667 TB cases; and all of them were initiated TB treatment. 4-Modules GeneXpert machines in Mobile-Vans contributed to low bacteriological-confirmed TB cases. The situation compromises early DR-TB case detection as well.

**Implication**

The results call for more investments in finding TB missing cases. At least 16-Module-Machines are ideal to reach “hard-to-reach” populations. Meanwhile, sputum-sample transportation from the event area to nearby TB diagnostic sites should be applied to increase bacteriological-confirmed cases and for early DR-TB detection.

## A77. Use of technology to provide continual education to sub-national level event-based surveillance coordinators in six high-risk regions in Tanzania mainland In 2021/2022

### Atuganile Musyani^1^, Lusungu Ngailo^1^, Rita Mutayoba^1^, Frida Ngalesoni^1^

#### ^1^Amref Health Africa in Tanzania, Dar es salaam, Tanzania

##### **Correspondence:** Atuganile Musyani (Atuganile.Musyani@Amref.org)

*BMC Proceedings 2023,* **17(13):** A77

**Background**

Delay in early detection, and reporting of Public Health Events of International Concerns (PHEICs) for early warning and rapid response (EWAR) and poor quality of data are among the challenges facing Integrated Disease Surveillance System (IDSR) in Tanzania. As a monitoring tool, different programs use physical mode of supportive supervision which is costly and time-consuming. Amref Health Africa through GHSA project piloted a model of alert reporting and supportive supervision using WhatsApp groups and virtual forums to ensure actors and decision-makers are reached within a short time while delivering information to many people required for rapid response.

**Methodology**

Between January and June 2022 project period, Amref facilitated creation of WhatsApp groups in 6 high-risk regions of Arusha, Dar es Salaam, Kagera, Kigoma, Mwanza, and Tanga comprising EBS implementers at national, regional, councils, health care workers (HCWs), and community health workers (CHWs). Upon detection, alerts are reported in WhatsApp group where the Health facility IDSR Focal Person conducts triaging and verification. On monthly basis, national, regional, and council EBS implementers conducted virtual supportive supervision to share alert data and field experience.

**Results**

A total of 31 councils created WhatsApp groups and 9 supportive supervision meetings were conducted. A total of 150 EBS coordinators and 1550 HCWs and CHWs were supervised. A total of 1856 alerts were reported whereby 1509 (81.3 %) were verified and 1338 (88.7 %) were confirmed to be PHEICs. Alerts reported included animal bites, skin lumps (suspected human/animal anthrax), respiratory illness, fever, and rashes.

**Conclusion**

Virtual alert reporting and supportive supervision have enhanced close monitoring resulting in better management of alerts at the community level. The mode allows smooth transition to real-time alert reporting through the electronic EBS application which is currently under final steps for piloting.

## A78. Demonstrating non-inferiority of lower dose calcium supplementation during pregnancy for reducing pre-eclampsia and neonatal outcomes in Tanzania

### Alfa Muhihi^1^, Mary Mwanyika Sando^1^, Ndeniria Swai^2^, Honorati Masanja^3^, Wafaie Fawzi^4^, Christopher Sudfeld^4^, Andrea Pembe^5^, Shabani Kinyogoli^1^

#### ^1^Africa Academy for Public Health, Dar es salaam, Tanzania; ^2^Regional Research Coordinator-Dar es Salaam, Dar es salaam, Tanzania; ^3^Ifakara Health Institute, Morogoro, Tanzania; ^4^Havard T.H.Chan School of Public Health, Massachusetts, United States of America; ^5^Muhimbili University of Health and Allied Sciences, Dar es salaam, Tanzania

##### **Correspondence:** Alfa Muhihi (amuhihi@aaph.or.tz)

*BMC Proceedings 2023,* **17(13):** A78

**Introduction**

Evidence shows that calcium supplementation during pregnancy decreases the risk of pre-eclampsia by more than 50% and reduces the risk of preterm birth. WHO recommends high-dose calcium supplementation (1500–2000 mg/day of elemental calcium) during pregnancy in populations with low dietary calcium intake. However, calcium supplementation is yet to become standard of care in most LMICs due to high cost of calcium supplements and complexity of the recommended dosing schedule.

**Methodology**

An individually randomized, double-blind trial of 11,000 eligible pregnant women will receive either standard high-dose (1500 mg/day) or lower-dose (500 mg/day) calcium supplementation. A parallel 250 participants will receive open-label calcium (1500 mg/day) at a separate health facility, while an observational cohort of 1000 participants will be assessed to observe the dose-response effect of calcium supplementation. Monthly follow-up visits will be conducted where haemoglobin, proteinuria, BMI, MUAC and supplementation adherence will be assessed. Study endpoints include pre-eclampsia and preterm delivery.

**Results**

This study will provide causal evidence on non-inferiority of lower-dose versus standard high-dose calcium supplementation during pregnancy. A single, lower-dose calcium supplementation has the potential to improve individual-level adherence, reduce costs hence enable adoption of this important intervention into routine antenatal care services in Tanzania.

**Conclusion**

Enrollment of 11,000 participants is complete, 732 observational cohort participants and recruitment of 250 participants for open label is eminent. Follow-up of enrolled participants is ongoing and expected to be completed in December, 2022.

## A79. Factors influencing enrolment into iCHF scheme in Tanzania

### Elizeus Rwezaura^1^, Manfred Stoermer^1^, Ally-Kebby Abdallah^2^, Sia Tesha^2^, Angel Dillip^3^

#### ^1^Swiss Tropical and Public Health Institute, Basel, Switzerland**;**^2^Health Promotion and System Strengthening project, Dodoma, Tanzania; ^3^Apotheker Consultancy (T) Ltd, Dar es Salaam, Tanzania

##### **Correspondence:** Elizeus Rwezaura **(**elizeus.rwezaura@hpss.or.tz)

*BMC Proceedings 2023,* **17(13):** A79

**Background/Rationale**

The Community Health Funds (CHF) is a voluntary community-based prepayment scheme aiming at contributing to a sustainable financing mechanism for health care **i**n Tanzania. The improved CHF (iCHF) was rolled out in 2018 the focus being on improving CHF structures, procedures, and benefit package and to increase enrolment in scheme. Despite these efforts, iCHF active coverage does not exceed 10% countrywide. This study assessed motivation behind enrolment into iCHF among active members, non-members and non-renewals necessary to inform strategies to increase enrolment

**Materials and Methods**

This was a mixed method cross sectional study design combining qualitative and quantitative methods. A multistage sampling technique was applied to identify 1503 respondents in five regions of Tanzania. Focus group discussions complemented quantitative data collection.

**Results**

Findings indicate gaps in knowledge on the difference between old and improved CHF. 57% of all study groups (including active members) were not aware of the difference between old and improved CHF. Cost saving (50%) and use of Insurance Management Information System-IMIS (25%) were the key reasons for joining iCHF among active members. Only 37% of non-members reported inability to pay the premium. Apparently iCHF was viewed as insurance for the poor by community members and most were willing to top up extra amount to have access to extended services. Only 17% of active members reported medicine stock-outs during their last visit at health facility. Knowledge on different iCHF packages from dispensary to referral levels was found to be low even among active members. Most of the study groups including active members thought availability of health facility in the village (89%) and IMIS (80%) increases iCHF attractiveness. Nevertheless, only 50% of active members were enrolled through Enrolment Officers (EOs), whom were also found inactive in study regions and mostly in Dar es Salaam. Majority of the study groups preferred treatment access at public and faith based health facilities.

**Conclusions**

Acting on the necessity of the current iCHF scheme to increase enrolment, iCHF need to promote iCHF packages, strengthen EOs accountability structure, increase iCHF premium and incorporate faith based health facilities into iCHF. To ensure increased premium and improved quality services, iCHF should make the scheme compulsory to everyone in the country.

## A80. Demand creation through LGA sensitization, local drama (PAS) lead to improvement of VMMC static sites perfomance in Geita region

### Kilemo Mzobora^1^, James J.Gesimba^1^, Florian Mutasingwa^1^, Dennis Christian^1^, Song'ora Biki^1^

#### ^1^Management and Development for Health, Dar es salaam, Tanzania

##### **Correspondence:** Kilemo Mzobora (kmzobora@mdh.or.tz)

*BMC Proceedings 2023,* **17(13):** A80

**Introduction**

For sustainability and utilization of health services in the community depends on the engagement of LGA. The demand creation messages and approaches were specifically for different ages.

Community sensitization with LGA and PAS drama group are essential to the success of VMMC program. This guides the design of service delivery modalities

**Methodology**

The demand creation through LGA sensitization was conducted in March-June 2022. We had eighteen community sensitization and advocacy meeting with local leaders and teachers. We had 12 PAS dramas group and local radios advertisement to sensitize males aged 15+ years to uptake VMMC at static facilities.

We conducted twelve community sensitizations by PAS drama group at all statics. We had local radio advertisement five times a day. MDH supported logistics for LGA leaders, PAS drama group and local radio advertisement.

**Results**

The intervention resulted in increased awareness of community surrounding 28 static clinics about the availability of VMMC services. The utilization of VMMC services at static facilities improved from 469(6%) at the end of December 2021 to 1759(54%) by the end of June 2022.

**Conclusion**

Researches recommended by WHO suggests that male circumcision reduces the chances of heterosexual transmission of HIV by 60%.VMMC programs focuses males aged 15-29 years the group at higher risk of acquiring HIV.MDH in collaboration with MoH work to achieve 100% coverage of VMMC in Geita. In October to December 2021 static sites contributed 6% of VMMC services. To improve this, MDH with CHMT had demand creation through community sensitization.

## A81. Contributing to achieving HSSP V- health supply chain and medicines management

### Allykebby Abdallah^1^, Fiona Chilunda^1^, Manfred Stoermer^2,3^ and Karin Wiedenmayer^2,3^

#### ^1^Health Promotion and System Strengthening (HPSS) Project, Dodoma; ^2^Swiss Tropical and Public Health Institute, Basel, Switzerland; ^3^University of Basel, Basel, Switzerland

##### **Correspondence:** Karin Wiedenmayer **(**Karin.wiedenmayer@swisstph.ch)

*BMC Proceedings 2023,* **17(13):** A81

**Background and objectives**

The objective of HSSP IV (2015-2020) was to reach all households with quality health care. HSSP V (2021-2025) aims to achieve the targets of the Sustainable Development Goals (SDG), leaving no one behind. The Swiss funded Health Promotion and System Strengthening project (HPSS) project supports the government since 2011, guided by national policy and strategic plans. This summary highlights the contribution of HPSS in the area of medicines management.

**Methodology**

Objectives and strategic objectives of HSSP IV and V were compared with interventions of HPSS project in medicines management.

**Results**

Strategic Objective 1 of HSSP IV:

The health and social services sector will achieve objectively measurable quality improvement of primary health care services, delivering a package of essential services in communities and health facilities. Areas proposed included 1) adequate staffing of primary health facilities, 2) coaching, mentoring and supervision by RHMT and CHMTs, 3) accountability, deterring pilferage and corruption, 4) adequate supply of medicines and health products.

HPSS project contributions: 1) increase in pharmaceutical staff and dispenser course for PHC, 2) peer cascade coaching and improved supervision tools, 3) effective inventory and financial auditing, 4) Jazia Prime Vendor System with increased medicines availability.

Strategic Outcomes in Health Systems of HSSP V: Achievement of SDG 3, health and wellbeing and coherence of building blocks for health. Outcomes proposed include: 1) quality training and performance of health staff, 2) medicines and supply chain, 3) Information technology, 4) health research, 5) public private partnerships. HPSS project contributions: 1) output and uptake of pharmaceutical dispensers, clinical training for therapeutic guidelines 2) national complementary Prime Vendor System, 3) IT solution for Jazia PVS, 4) research publications and practice to policy translation, 5) successful adoption of health PPP

**Conclusion**

Activities implemented by the project align with strategic objectives if HSSP IV and V. Successful innovations such as the Jazia PVS were rolled-out nationally, while other interventions strengthened health systems regionally and influenced national guidelines and policies, such as training and courses for health staff, supervision, coaching, auditing, therapeutic guidelines, promotion of responsible use of medicines and antimicrobial resistance mitigation. Pharmaceutical activities of HPSS project consistently adhered to objectives of the national strategic plans and significantly contributed to strategy achievement.

## A82. The difference in healthcare utilization between population insured and not insured through iCHF in Tanzania

### August Kuwawenaruwa ^1^

#### ^1^Swiss Tropical and Public Health Institute, Basel, Switzerland

##### **Correspondence:** August Kuwawenaruwa

*BMC Proceedings 2023,* **17(13):** A82

**Background**

Implementation of Community Based Health Insurance in developing countries seeks to improve the access and utilization of healthcare services by removing financial barriers among the poor households.

**Objective**

To examine the difference in utilization between population insured and not insured through improved Community Health Fund (iCHF) in Tanzania.

**Methods**

Quantitative data extraction from the health facility records (5 Regional Referral Hospitals, 5 District Hospitals, 17 public health centres and 20 public dispensaries) and in-depth interviews were conducted with the healthcare managers, and with providers in April 2022.

**Results**

Results showed that in urban settings, more clients were those who were paying out-of-pocket while in rural settings more were within the exempted category. Most of iCHF beneficiaries accessed primary healthcare facilities (dispensary, health centre, district hospital), with limited access to regional referral hospitals. At the primary facility, iCHF beneficiaries are entitled to all the services available while at a higher level of care services are limited and they have to make co-payments. The difference in access and utilization of healthcare services between iCHF beneficiaries and those paying out-of-pocket was attributed to the number of people enrolled within the facility catchment population.

**Conclusion**

There is a difference in access and utilization of healthcare services between iCHF beneficiaries and those paying out-of-pocket in the surveyed facilities. This indicates the need for continuous education of the community around the facility catchment areas to enrol into the iCHF scheme. Furthermore, improvement in service provision would attract more iCHF beneficiaries, however, there is a need to closely monitor the amounts of co-payments charged to the iCHF beneficiaries to assess their detrimental effect to the scheme.

## A83. Addressing undernutrition in children using positive deviance hearth strategy in Njombe region

### Generose Mulokozi^1^, Teddy Mamboleo^2^, Lali Chania^2^, Emmanuel Sanga^2^, Magreth Paul^3^

#### ^1^IMA World health, Dar es Salaam, Tanzania; ^2^IMA World, Njombe, Tanzania; ^3^PANITA, Dar es Salaam, Tanzania

##### **Correspondence:** Generose Mulokozi (Gmulokozi@imaworldhealth.org)

*BMC Proceedings 2023,* **17(13):** A83

**Introduction**

Malnutrition is a public Health problem in Tanzania affecting mainly children and women of reproductive age. According to the NNS of 2018, Njombe region has higher rates of undernutrition among children whereby 53.6% are stunted. Positive Deviance Hearth (PDH) is a community-based model for rehabilitating underweight children and enable the families to sustain the rehabilitation at home leading to weight gain.

**Methodology**

IMA World Health and partners in collaboration with the Local Government Authorities with support from UNICEF, implemented a one-year project in Njombe for improving Maternal and Child nutrition. Among the interventions implemented is PDH. PDH uses the ‘Positive Deviance’ approach to identify the positive behaviours practiced by the caregivers of well-nourished children from poor families and transfers such behaviours to other caregivers in the same community whose children are undernourished. Its implementation involves nine steps;1) Determining feasibility 2) Community mobilization 3) Situational analysis, 4) Positive Deviance Inquiry 5) Design hearth sessions 6) Hearth sessions 7) Follow up visits 8) Monitor progress and 9) Decide expansion or exit of PDH. It was piloted in two Councils of Njombe namely Ludewa and Wanging’ombe, involving six villages per Council.

**Results**

A total of 313malnourished children from 12 villages were enrolled in PD hearth with completion rate was 88%. 67% and 80% of the children gained required weight within 12 days and 90 days of the training and rehabilitation respectively. Participation in PDH improved care practices among care givers.

**Conclusion**

Implementation of PDH model was successful as it involved community structures using the locally available foods which were contributed by care givers themselves. Involvement of community leaders at all stages and follow up by multisectoral teams at council level made the intervention successful. Councils could include PDH in annual workplans as one of the feasible activities to address undernutrition.

## A84. From zero to thirty-one men having sex with men: peer -led recruitment initiative in Geita region

### Dina Gerald^1^, Elizabeth Herman^1^, David Kikuli^1^, Nicholaus Tarimo^1^, Rosemary Khamsini^1^

#### ^1^Management and Development for health, Dar es salaam, Tanzania

##### **Correspondence:** Dina Gerald (dgerald@mdh.or.tz)

*BMC Proceedings 2023,* **17(13):** A84

**Introduction**

In achieving treat all goal reaching out to Men who have sex with Men group is still suboptimal due to stigma and social context. This hinders effort on reaching epidemic control. Thus, up to February 2022, no MSM had been identified and documented in Geita region due to its context and community stigma of the group. Hence, we conducted a peer-led recruitment initiative in identifying and reaching MSM.

**Methodology**

Peer-led recruitment initiative was done through recruiting a peer, living about 124 km from Geita region, based on his vast experience in MSM networks. The peer reached and convinced MSM in Geita region to disclose his status and work with MDH. In return the two of them mapped MSM’s hotspots, created awareness and did demand creation towards HIV testing and preventive services.

**Results**

Out of this initiative, 5 hotpots were identified by the help of the peers together with Village and Ward officers,5 meetings for awareness and demand creation were done, 8 visits to hotspot were done to identify MSM. By June 2022, 31 MSM were identified, of which 16 were tested for HIV, 1 MSM were newly diagnosed with HIV/AIDS and linked to care and treatment clinics and 15 were linked to Pre-Exposure Prophylaxis (PrEP) services and provided with condoms.

**Conclusion**

Peer led recruitment initiative worked effectively and successfully in reaching and linking the MSM to HIV preventive and treatment services through peers who broke some barriers to access to care identifying MSM. We recommend scaling up the use of peers for timely linking of MSM to appropriate HIV care services.

## A85. Role of community antiretroviral therapy champions in scaling up community refill service among PLWHIV in Geita region; afya jumuishi project

### Aloys Ndamugoba^1^, Dina Gerald^1^, Florian Mutasingwa^1^, David Kikuli^1^

#### ^1^Management and Development for Health, Dar es salaam, Tanzania

##### **Correspondence:** Aloys Ndamugoba (andamugoba@mdh.or.tz)

*BMC Proceedings 2023,* **17(13):** A85

**Background**

Community ART refill is important in reaching underserved population and reducing treatment burden among stable PLHIV. CDC recommends 20% of PLHIV to be served at community. Until January 2022, only 4.5% of PLHIV on treatment refilled at community resulting to increased treatment interruptions, affecting efforts towards achieving 95-95-95 by 2030. Routine sensitization through peers were conducted. However, results were unconvincing. Community ART champions model was introduced to scale up uptake.

**Methodology**

A total of 30 community ART champions were identified among willing, and disclosed PLHIV with good adherence on ART, and mentored for community ART Uptake. They conducted 3 one to one session with clients to understand needs and identify barriers for community ART services. They play a link up role to health care providers and facility which has increased demand for community ART services. Formed ART groups basing on client’s specific needs. MDH supported transport and communication incentives.

**Results**

After introducing champions model, community ART uptake among PLHIV in Geita region increased from 4.5% as of January to 23% by June 2022. Through 100 community ART champions, 20,800 stable PLHIV were sensitized for community ART refill of which 16,731 clients accepted and enrolled on the service, contributing to 23% of the total PLHIV currently on treatment. This has in turn improved appointment adherence to 99% by June 2022, enhanced retention on care among PLHIV, decongested health facilities, enabled HCWs to concentrate on clients who need close follow up and care and thus contributed to good health and wellbeing.

**Conclusion**

Community ART champions model worked effectively in improving the uptake of community ART among PLHIV in Geita region and we recommend to be adopted by partners and stakeholders implementing care and treatment services. However, there is cost implication as champions need to be supported with transport and communication incentives.

## A86. Peer client bond in accelerating the return of unassisted HIV self-test kits among female sex workers. Afya jumuishi Geita region

### David A. Kikuli^1^, Aloys Ndamugoba^1^, Ally Mawanja^1^, Isaya Kuchusa^1^, Nicholaus Tarimo^1^, Elizabeth Herman^1^, Dina Gerald^1^, Vasilisa Joseph^1^

#### ^1^Management and Development for health, Dar es salaam, Tanzania

##### **Correspondence:** David A. Kikuli (dkikuli@mdh.or.tz)

*BMC Proceedings 2023,* **17(13):** A86

**Introduction**

HIV-self test (HIVST) is a gateway approach to accessing current HIV testing services. Assisted,unassisted modalities recommended by WHO on reaching key and vulnerable populations. By January 2022, the return rate of unassisted kits among FSW was 66% in region far from 100% expected, caused by anticipated stigma,timewasted,distress and livelihood loss on reactive results negatively impacting achievement three 95s.MDH with R/CHMT initiated peer-client bond to accelerate return of unassisted HIV self-test kits.

**Methodology**

Peer-client bond was introduced by recruiting peers among targeted FSW and were paired according to their age and locality, trained on KVP friendly service and HIV self-test framework. One peer was linked to 3 to 4 beneficiaries and were fully engaged for demand creation, pre-distribution informative counselling 3 to 4 days with clients, ascertaining possible perceptions on outcomes of the tests and engaged in tracking unreturned kits by phone contacts and home visits. MDH supported transport and communication incentives.

**Results**

Return rate of unassisted HIV self-test kits distributed among FSW accelerated from 66% in January to 99% by June 2022.Through 50 recruited peers, a total 3899 FSW were served with unassisted kits from February 2022 to June 2022,3860 FSW equivalent to 99% were able to return their kits timely with 638 reactive and confirmed kits contributing to 16% of newly identified and linked FSW to care in the region, a significant contribution towards achievement of third United Nations Sustainable Development Goal by 2030.

**Implication**

Peer-client bonding is a key in accelerating the return of unassisted HIVST kits as it prompts auto barriers and experience sharing, eliminating probable fear of stigma and livelihood loss when HIV status is known. It is important on optimizing HIV testing services and prevention services among female sex workers.

## A87. Impactful community engagement in early TB case detection in Tanzania - Experience from Amref Health Africa

### Lilian Theodore Ishengoma^1^, Amos Nyirenda (MD)^1^, Amani Maro^1^, HassaniMataka (MD)^2^, Frank Ntogwisangu^2^, Anna Kiravu^1,^ John Msaki^3^

#### ^1^Amref Health Africa, Dar es salaam, Tanzania; ^2^Management Development for Health, Dar es salaam, Tanzania; ^3^National TB and Leprosy Programme, Dodoma, Tanzania

##### **Correspondence:** Lilian Theodore Ishengoma (Lilian.Ishengoma@Amref.org)

*BMC Proceedings 2023,* **17(13):** A87

**Introduction**

Tanzania is among the 30 high TB burden countries with the incidence of 222/100,000 and 85,603 (64%) treatment coverage, which indicates (36%) of estimated cases remained undiagnosed in communities (WHO,2020). AMREF and its partner MDH are implementing the HIV/TB Global-Fund grant (2021- 2023) in 8 regions (Tanga, Simiyu, Mara, Dodoma, Shinyanga, Mbeya, Ruvuma and Kagera) focusing on community-engagement to find TB-missing cases.

**Methodology**

Key community TB interventions applied include Community Active TB case Finding (Door to door, Community Campaigns; Contact Investigation) and Sputum sample transportation from non-TB diagnostic sites/ Community to GeneXpert sites. Occasionally, TB services through Mobile Van TB clinics have been provided in hard to reach areas. 960 Accredited Drug Dispensing Outlets and 640 Traditional healers have been engaged and linked to a total of 1920 CHWs who were trained on the key interventions including TB screening and referrals.

**Results**

TB notified cases were increased from 5255 (Jan- March 2021) to 9402 (April – June 2022) in implementation area. Community contribution in TB case notification increased to 61% (April – June 2022) compared with the baseline of 23% in the first quarter, 2021. This is above the national target of 30% community contribution in TB case notification. Transportation of sputum samples maintained bacteriological confirmed TB cases at 54.2%, despite of increased number of TB cases.

**Implication**

Community engagement is a key for finding missed TB cases. The country should invest more on community engagement to achieve END TB targets. Transportation of specimen from non-TB diagnostic sites/ community to diagnostic sites increases access to molecular test which is the first line TB diagnosis technology.

## A88. Finding missing people with tb at local brew pubs: Experience from Amref USAID Afya shirikishi project from May 2021 to March 2022 - Mwanza region, Tanzania

### Edward Chilolo^1^, Rose Olotu^1^, Godwin Munuo^1^, Michael Machaku^1^, Thomas Nkwabi^2^, William Nangi^3^, John Msaki^4^, Pascal Wilbroad^5^

#### ^1^USAID AfyaShirikishi project, Amref Tanzania, Dar es salaam, Tanzania; ^2^USAID AfyaShirikishi project, SHDEPHA+ Kahama Tanzania; ^3^RTLC, Mwanza, Tanzania; ^4^National TB/Leprosy program, Dodoma, Tanzania; ^5^USAID Tanzania, Dar es salaam, Tanzania

##### **Correspondence:** Edward Chilolo (Edward.Chilolo@Amref.org)

*BMC Proceedings 2023,* **17(13):** A88

**Background**

Amref in Tanzania, through USAID AfyaShirikishi project in Mwanza region in collaboration with Ministry of Health through National Tuberculosis and Leprosy Program (NTLP) and Regional/Council Health Management Team is implementing community based TB services aiming at finding missing people with TB among vulnerable and underserved risk population with focus to individuals accessing services at local brew pubs. According to WHO TB Global Report (2020), Tanzania misses 48,2099 (36%) TB cases annually from the WHO estimated 133,000 cases with treatment coverage standing at 64%. TB screening activity at local brew pubs was done to find missing people with TB among individuals attending there. Little has been done in finding missing TB cases in this population group.

**Methodology**

Mwanza is among the region with a large number of local brew pubs in Tanzania which is not well documented. From May 2021-March 2022 Mwanza region TB team conducted purposely TB screening in 74 Local brew pubs through ward/village executive officers. The project engaged 44 community health workers to provide community TB health education and screening at local brew pubs in four districts. Presumptive TB individuals were identified, sputum specimen collected on the spot and transported for laboratory TB investigation. Those with symptoms but unable to produce sputum were given referral to the nearby diagnostic facility for TB investigation.

**Results**

The project implementation from May 2021 to March 2022 shows a total of 1930 clients were reached and 85% (1650) were screened for TB. Among them 25% (406) were TB presumed, 11% 41(F3:M38) were found with active TB were initiated TB treatment. This shows there is a good number of TB cases among local brew alcoholic.

**Conclusion**

In order to address the gap in finding missing TB cases at community level, all vulnerable groups should be reached with TB services. NTLP should consider to include local brew pubs as one of the TB hotspots.

## A89. Comparative assessment on community led and facility led approach on increasing number of covid 19 vaccines uptake in Simiyu region

### Yasinta Bahati^1^, Aisa Muya^1^, Annagrace Katembo^1^, Yasinta Bahati^1^, Rita Mutayoba^1^, Florence Temu^1^, Paschal Nyagonde^1^

#### ^1^Amref Health Africa, Dar es salaam, Tanzania

##### **Correspondence:** Yasinta Bahati (Yasinta.Bahati@amref.org)

*BMC Proceedings 2023,* **17(13):** A89

**Introduction**

Background: Tanzania recorded the first case of COVID 19 on March 16th 2020 in Arusha Region. Early months of the pandemic, Tanzanian adopted some WHO recommended COVID 19 preventive measures but decided not to implement a lockdown measure. As vaccination become available, the government decided to join other countries in vaccination amid it being late. Simiyu Region was among regions with low vaccination Performance of 3.3% by December 2021. With support from Africa CDC, Amref Tanzania implemented both community led and facility led approaches in the efforts towards increasing vaccination uptake in the region.

**Methodology**

Methodology: Comparative approach on assessing number of COVID 19 vaccines uptake in two different approaches was deployed in Simiyu region. Community led approach, involving campaigns with connection of community leaders, Community Health workers and Health Care workers as vaccinators was deployed in December 2021 to February, 2022. On the other hand, facility led approach involved health facilities to own vaccination activities to their catchment areas with usual Regional and Council health management team supervision activities that bring ownership to R/CHMT during implementation of COVID 19 vaccination activities. Approach deployed in March to May 2022.

**Results**

Results: Over three months of implementation for each approach, number of doses administered during facility lead approach was significantly higher (280,226) as compared to Community led approach (117,797) with increased number of fully vaccinated individual of 122,091, compared to community led 52,946 Individuals moving the region coverage from 3.3% by December 2021 to 32% by July 2022.

**Implication**

Conclusion: Facility led approach have shown to be most successfully approach on COVID 19 vaccination across the region than community led approach. More research is required in assessing explanatory factors to these delivery models.

## A90. Seroprevalence and factors associated with Syphilis infection among pregnant women living with HIV/AIDS in Mtwara

### Vulstan J. Shedura^1^, Doreen Kamori^2^, Geofrey Mchau^3^

#### ^1^ Tanzania Field Epidemiology and Laboratory Training Program, Dar es Salaam, Tanzania; ^2^ Department of Microbiology and Immunology, Muhimbili University of Health and Allied Sciences, Dar es Salaam, Tanzania; ^3^Tanzania Food and Nutrition Centre, Dar es Salaam, Tanzania

##### **Correspondence:** Vulstan J. Shedura (vulstanshedura@gmail.com)

*BMC Proceedings 2023,* **17(13):** A90

**Background**

Syphilis is an infection caused by spirochete Treponema pallidum, which can be transmitted perinatally causing congenital syphilis. Several studies in developing countries have shown that syphilis in pregnancy is a public health problem leading to perinatal morbidity and mortality. However, data on syphilis is limited in Tanzania and especially among pregnant women living with HIV/AIDS (PWLHIV). The study aimed to determine seroprevalence and factors associated with Syphilis infection among PWLHIV.

**Methodology**

A health facility-based cross-sectional study was conducted among pregnant women living with HIV/AIDS (LWHIV) in selected health facilities in Mtwara region. The study was conducted for 3 months. A structured questionnaire was used to collect information clinical information from the study participants. A 4 mls of blood was collected for Syphilis screening and confirmatory tests using rapid tests and automated ELISA tests respectively. A logistic regression model was used to determine factors associated with Syphilis infection in pregnant women LWHIV.

**Results**

Two hundred and twenty (n=220) pregnant women living with HIV/AIDS were enrolled in this study, with a median age of 32.7 years (IQR: 27.6-37.6). The majority (45.5%) of the participants were married, 71.4% had primary education, 47.7% were unemployed, 77.7% were multigravida and 40.5% were in second trimester. The seroprevalence of Syphilis infection and Syphilis-hepatitis B co-infection was 10.9% and 5.9 respectively. In this study, being in the second trimester of gestation period [aOR=5.69: 95% CI 1.44-22.46, p=0.013] and being infected with hepatitis B [aOR=31.39: 95% CI 9.45-104.23, p<0.001] were independently associated with HBV infection among PWLWHIV in Mtwara region.

**Conclusion**

This study revealed that seroprevalence of Syphilis infection and Syphilis-hepatitis B co-infection is 10.9% and 5.9 respectively. Additionally, being in the second trimester and being infected with HBV are factors associated with Syphilis infection among PWLHIV in Mtwara region. Therefore, scaling up Syphilis screening is crucial to prevent vertical transmission.

## A91. Rapid scale-up of 6 multi-months dispensing among art clients in the southern highlands, Tanzania

### Emilian Ng'wand^1^, Sallyy Chalamila^1^, Patricia Agaba^3^, Amos Nsheha^1^, Janet Mwambona^2^, Raymond Majengo^2^, Diana Nicholaus^2^, Sarah Feinman^2^, David Maganga^1^

#### ^1^ HJF Medical Research International (HJFMRI), Mbeya, Tanzania; ^2^Walter Reed Army Institute of Research, US Department of Defense, Tanzania; ^3^Department of International HIV Prevention and Treatment (IHPT), Bethesda, MD, USA

##### **Correspondence:** Emilian Ng'wand (engwandu@wro.or.tz)

*BMC Proceedings 2023,* **17(13):** A91

**Introduction**

Tanzania ranks among top five countries for HIV burden in Africa with an estimated 1.7 million people living with HIV. The Henry M. Jackson Foundation for Medical Research International (HJFMRI) supports four regions in the Southern Highlands which account for 11.3% of the country’s clients for anti-retroviral treatment (ART). The scale-up for 6MMD started in September 2021 with the target to reach 60% of ART clients by March 2022.

**Methodology**

HJMFRI had 6,924 clients (3.6% of clients on ART) receiving 6MMD and viral load coverage of 83.8%. After 6 months of rollout in March 2022, managed to place 118,525 (60%) clients on 6MMD while maintaining 93% retention, 95% viral suppression, and 88% viral load coverage. Among the encountered barriers include weight changes for pediatrics aged 5 – 9.9 years who required dose adjustment at least once, and some of the clients missed the opportunity for 6MMD initiation due to inadequate knowledge of HCWs on eligibility criteria.

**Results**

HJMFRI had 6,924 clients (3.6% of clients on ART) receiving 6MMD and viral load coverage of 83.8%. After 6 months of rollout in March 2022, managed to place 118,525 (60%) clients on 6MMD while maintaining 93% retention, 95% viral suppression, and 88% viral load coverage. Among the encountered barriers include weight changes for pediatrics aged 5 – 9.9 years who required dose adjustment at least once, and some of the clients missed the opportunity for 6MMD initiation due to inadequate knowledge of health care workers on eligibility criteria.

**Implication**

HJFMRI was able to meet its 6MMD goals using various tools including creating and sharing a roadmap using CME, gaining full stakeholder engagement, providing mentorship, and weekly monitoring.

## A92. Successful return of client’s results after an HIV self-test: the role of peer educators in southern highlands, Tanzania

### Abel Ngwalle^1^, Amos Nsheha^1^, Sally Chalamila^1^, Henry Musila^1^, Merensiana Mvungi^1^, Hijja Wazee^1^, Nassoro Yahaya^1^, David Maganga^1^

#### ^1^HJF Medical Research International (HJFMRI), Mbeya, United Republic of Tanzania

##### **Correspondence:** Abel Ngwalle (angwalle@wrp.or.tz)

*BMC Proceedings 2023,* **17(13):** A92

**Introduction**

Henry Jackson Foundation for Medical Research implemented a peer led HIVST targeting hotspots for kit distribution. Trained peers screened clients and those eligible offered HIVST kits for their own use and extra kits for their contacts. Clients’ contact information was recorded in the HIVST registers. Within 2 days, peers called clients to inquire about kits, unreachable clients traced physically. Clients with reactive results were referred to a nearby clinic for confirmatory testing. Newly positives were escorted for antiretroviral therapy initiation.

**Methodology**

Tanzania adopted HIV self-testing (HIVST) as an integrated approach to national HIV testing services (HTS) in 2019. HIVST is being scaled up as a safe and effective strategy to reach undiagnosed populations. While HIVST has the potential to increase uptake of HTS and help detect early HIV infection, monitoring the utilization of HIVST kits and the outcome of end-users remains challenging.

**Results**

Within six (6) months, peer educators distributed 19,918 HIVST kits, with 10,486 (53%) clients preferring directly assisted peers adding information on the use of HIVST (8,672 females and 1,814 males) while 9,432 clients (5,922 females and 3,510 males) opted for unassisted self-testing. Overall, 15,987 (80%) clients returned their kits, and among them, 359 (2%) clients had reactive results. Follow-up testing using the national testing algorithm confirmed 351 (98%) as HIV-positive and nearly all newly positives (99.7%) were linked to ART. During the reporting period.

**Conclusion**

The model reported a high accuracy and acceptable HIV performance, with a high return rate of distributed kits. Integrating HIVST to facility-based HTS may further increase the frequency of testing and improve case findings, particularly among those who would be missed from conventional HIV testing approaches.

## A93. “I had no choice, I had to be very strong”: Health care provider perspectives on treatment of COVID-19 patients in Tanzania

### Muzdalifat Abeid^1,2^**,** Bernard Kivuma^1^, Gregory Ntiyakunze^1^, Ali Khatau^1^, Christian Mwalo^1^, Mohamedraza Ebrahim^1^, Sherin Kassamali^1^, Eunice Pallangyo^2^

#### ^1^Aga Khan Hospital, Dar es Salaam, Tanzania; ^2^Aga Khan University, Dar es Salaam, Tanzania

##### **Correspondence:** Muzdalifat Abeid

*BMC Proceedings 2023,* **17(13):** A93

**Background:** The World Health Organisation has characterised coronavirus disease (COVID-19) as a global pandemic. Several measures have been taken to manage patients, including the use of off- label medications as well as herbal medications. Despite different treatment regimens, there were other aspects related to fatality of the disease, namely, fear and challenges faced by healthcare providers in settings with limited resources. Hence, the perspective of the frontline healthcare providers may enlighten us on any clinical benefits of treatment regimens and further inform policy makers to consider strategies that will regulate off-label practises.

**Methods:** A qualitative study using focus group discussions (FGD) was employed at an urban hospital with 24 healthcare providers who were caring for patients with COVID-19. An FGD guide was utilised to extract in-depth data. Topics covered were challenges and fears associated with treatment of COVID-19, views on off-label drug use and herbal treatment, and providers’ experiences of patient management. FGDs were transcribed verbatim and inductive thematic analysis was used to develop meaningful themes.

**Findings:** Provider perspectives were summarised into 3 themes. First, negative emotional responses, especially in the first wave, consisted of fear and anxiety caused by working in the COVID units, perceived risk of getting infected and concern for patients and their relatives. Second, COVID-19 healthcare delivery was multidisciplinary and consisted of rapidly evolving treatment protocols, symptomatic and supportive care. Third, we found several patient-related challenges which included stress, and community responses towards COVID-19.

**Conclusion:** Healthcare providers perceived fear and anxiety as initial responses. Flexibility and multidisciplinary team involvement played a great role in ensuring patient care as well as provider wellness. Therefore, important policy measures should be kept in place to ensure pandemic preparedness to help providers carry out their duties.

## A94. Multiple approaches in WASH for quality of health services delivery – Geita case study

### Neema Kimaro^1^, Christina Mhando^1^

#### ^1^WaterAid Tanzania, Dar es salaam, Tanzania

##### **Correspondence:** Neema Kimaro (neemakimaro@wateraid.org)

*BMC Proceedings 2023,* **17(13):** A94

**Background**

Access to WASH in HCF increases trust in the health services to give birth in facilities in comfort and safety as opposed to at home.

The inclusion of different stakeholders from the project design to inception is very important in enhancing participation, ownership, awareness, and sustainability of the project.

**Methodology**

Project delivery included the following activities:

Constructing WASH infrastructure at 12 HCF, increasing running water within facilities, toilets and handwashing stations to the maternity wards, operating rooms, and other areas within the facility.

Training 1,906 community health workers and skilled birth attendants on the provision of gender-sensitive services and best practices in WASH.

Awareness-raising campaigns on available WASH services using local influencers and artists.

Orienting local leaders on WASH issues for improved budgeting and planning for WASH in HCF.

**Results**

Through a baseline study, we determined limited access to water in HCFs in Geita and Nyang’whale districts. Hygiene practices were difficult to uphold and IPC impossible to sustain. Availability and use of WASH facilities in HCF was an area to be addressed to minimize disease transmission. We implemented a “Canada-Africa Initiative to Address Maternal, Newborn and Child Mortality (CAIA-MNCM)’ project in Geita and Nyang’whale districts, where we built WASH infrastructures in HCF and focused on generating change through engaging with the community and government advocates.

**Conclusion**

These measures have contributed to improved access to sanitation and hygiene services and a decrease in sepsis cases. Increased the coverage (59% to 78%) of delivery services among women in HCFs with a live birth in the two years preceding the survey in Geita and Nyang’whale districts, at baseline (2016) and endline22 (2020). The end-line evaluation for the project indicated that HCF has included the project activities in their plans – such as repair and maintenance of infrastructure built by the project.

## A95. Expanding access and utilization of long-acting reversible contraceptives and permanent methods through family planning outreach services; a case study of Songwe and Rukwa regions, Tanzania

### Julius Njunwensi^1^, Otmar Massawa^1^, Christian Ndomba ^1^, Kasanga Mkambu^1^, Annette Almeida^1^, Selina Mathias^2^, Boniphace Kasululu ^3^Eva Mwamkyusa ^3^Asha Resa Izina^4^

#### ^1^Tanzania Health Promotion Support (THPS), Dar es salaam, Tanzania; ^2^USAID – Tanzania, Dar es salaam, Tanzania; ^3^ Regional Health Management Teams (RHMT), Songwe region, Tanzania; ^4^Regional Health Management Teams (RHMT), Rukwa region, Tanzania

##### **Correspondence:** Julius Njunwensi (jnjunwensi@thps.or.tz)

*BMC Proceedings 2023,* **17(13):** A95

**Background**

Songwe and Rukwa are among the four regions that are beneficiaries of USAID Uhuru TB and FP Facility Solutions Activity LON implemented by THPS supported by USAID. Both regions are faced with a critical shortage of skilled providers for the provision of family planning (FP) services, especially long-acting reversible contraceptives (LARC) and permanent methods (PM).

**Methodology**

USAID Uhuru Activity collaborated with R/CHMTs coordinated provision of facility-led FP outreach services in health facilities with scarce FP skilled personnel. A retrospective cross-sectional study involved analysis of FP routine and outreach service delivery data (for Songwe and Rukwa Regions) from DHIS2 and computation of Couple Years of Protection (CYP) for the period of 1st October 2020 and 30th September 2021. Microsoft Excel and The Chi-square (x2 shortcut method) were used in analysis.

**Results**

During the implementation, cumulatively, newer LARC/PM acceptors served through outreach services were higher as compared to those served through routine services (102,640 vs. 41,757; x2 =258.45 on df=1, p<0.01). Likewise, CYP also was three times higher 318,84 vs. 121,205 respectively (x2=1222.31 on df=1, p<0.01). Based on these findings, hypotheses were rejected and concluded that there were statistically significant associations between FP outreach services and LARC/PM uptake and CYP.

**Conclusion**

FP outreach services contributes significantly to improving access and utilization of LARC/PM uptake, especially in hard-to-reach rural communities with scarce skilled personnel. Government and IPs are encouraged to invest in FP outreach services.

## A96. Tackling tuberculosis among people who inject drugs (pwid) -experience from juza plastic recycling company from october 2021 to june 2022 in ilala: Dar es Salaam, Tanzania

### Esther Mukasa^1^, Linda Mutasa^2^, Florence Temu^1^, Michael Machaku^1^, Seif Mbarouk^2^, Rose Olotu^1^, Aisa Muya^1^, Frida Ngalesoni^1^, Godwin Munuo^1^

#### ^1^Amref Health Africa in Tanzania, Dar es salaam, Tanzania; ^2^Ilala MC/Dar city Council, Dar es salaam, Tanzania

##### **Correspondence:** Esther Mukasa (esther.mukasa@amref.org)

*BMC Proceedings 2023,* **17(13):** A96

**Introduction**

According to the 2020 WHO TB Global Report, Tanzania misses 48,2099 (36%) TB cases annually from the WHO estimated 133,000 cases with treatment coverage standing at 64%. Globally, People Who Inject drugs (PWIDs) are among the most risk populations for TB, with TB infection prevalence ranging from 10% to as high as 67%, Tanzania is not spared.

**Methodology**

Community Health workers reached PWIDs at the company where they sell used plastic bottles. Twice a week, TB screening was conducted by CHWs using a standardized TB screening questionnaire to PWIDs. Presumptive TB individuals’ sputum specimen were collected on the spot and transported for investigation. Those who were not able to produce sputum were referred for further investigation at MnaziMmoja Hospital. Those diagnosed with TB were initiated on TB treatment and linked with JUZA company supervisor for follow up on adherence to medication.

**Results**

During the implementation a total of 517 PWIDs were screened for TB and 52% were presumptive TB cases. Among presumptive cases, 9% were diagnosed to have TB and all were men. This shows high burden of TB in PWID. All TB cases were initiated treatment.

**Implications**

Tailored ACF among TB key population like PWID is of paramount importance in finding missing people with TB in the community.

## A97. Male involvement in PMTCT services: the use of influential men to promote PMTCT services in Tanzania

### Neema Makyao^1^, Amani Maro^1^, Mary Nzowa^1^, Frida Ngalesoni^1^, Nyirenda Amos^1^, Kosia Agnes^2^

#### ^1^Amref Health Africa, Dar es salaam, Tanzania; ^2^Christian Social Services Commission, Dar es salaam, Tanzania

##### **Correspondence:** Neema Makyao (neema.makyao@Amref.org)

*BMC Proceedings 2023,* **17(13):** A97

**Introduction**

Globally, male involvement has been identified as a priority area to be strengthened in the prevention of mother to child transmission (PMTCT) of HIV. Since implementation of PMTCT, there has been notable high HIV testing coverage in ANC, with 98% of pregnant women knowing their HIV status in 2021. However, more progress needs to be achieved with regards to the proportion of women attending ANC who had their male partners tested for HIV which is currently at 68% as per program data. Amref uses role model fathers/ influential men who influence the community and strengthen the linkage between the community and PMTCT health facilities.

**Methodology**

We conducted meetings with community leaders involving street and village leaders. Influential men were identified by the village leader and recruited by the project with positive attitudes towards support for women on RCHS and PMTCT. We recruited 277 role model fathers, trained and capacitated in influencing the community on PMTCT services and strengthening the linkage between the community and PMTCT health facilities by promoting, advocating, and facilitating male partner engagement in early booking at first antenatal care.

**Results**

A total of 51,142 pregnant women tested for HIV at ANC, and 36,888 (72%) tested with their male partners at ANC services from January to March 2022. There is an increase in male partner testing rates across the regions. However, Geita region is leading by 90% followed by Shinyanga (86%), and Pwani by 79% and Arusha being the least performing by 50%. This is attributed by influential men sensitizing males to participate in ANC services and promoting male partner testing to increase male engagement in ANC/PMTC.

**Complications**

To enhance male involvement, the eMTCT strategy need to ensure that facilities implement integrated male HIV/SRHR service in the PMTCT. Such strategy will establish a people centered service delivery that attracts male to participate.

## A98. Enhancing detection and reporting of public health events of international concern in Zanzibar for early warning and rapid response

### Lusungu Ngailo^1^, Atuganile Musyani^1^, Rita Mutayoba^1^, Frida Ngalesoni^1^

#### ^1^Amref Health Africa, Dar es salaam, Tanzania

##### **Correspondence:** Lusungu Ngailo (Lusungu.Ngailo@Amref.org)

*BMC Proceedings 2023,* **17(13):** A98

**Background**

Zanzibar has limited capacity to detect outbreaks and this weakens its ability to promptly respond and control public health threats or events in a timely fashion, especially at community level. Despite success in indicator-based surveillance priority on other diseases have not been established. Amref Health Africa funded by CDC, in collaboration with the MoH in Zanzibar developed a model for Event-Based Disease Surveillance (EBS) to bridge the health gap between community and formal health system. The five-year project being conducted will empower the communities to detect report and participate in the response to disease outbreaks.

**Methodology**

Between September and December 2021, Amref collaborated with Ministry of Health Zanzibar to develop national EBS guidelines and training materials, sensitize 240 local influential leaders and train 29 surveillance officers from four districts of Chakechake, Wete, Mkoani and Micheweni on EBS. Establishment of alert management desks was done through procurement and installation of furniture and ICT materials. The EBS model was set up by linking district surveillance officers with health care workers (HCWs). Submission of EBS data from community to districts alert management desks is conducted through alert registers and toll-free number. The verified events are managed at local levels by district surveillance officers in collaboration with the healthcare workers.

**Results**

12 TOTs were trained; who cascaded training to 18 alert management desk officers. Between January 2021 and June 2022, out of 248 alerts that were reported from health facilities 183(75.3%) were verified timely. Among the managed events includes 23 suspected cases of food poison, 16 of COVID-19, and 3 suspected cases of measles.

**Conclusion**

Implementation of EBS has strengthened management of public health events in Pemba. There is opportunity of scaling up electronic EBS to the community level using availability of electronic system at community level (DITRA).

## A99. Prevalence and factors associated with poor knowledge of sexually transmitted infections among secondary school students in Moshi municipal, Kilimanjaro region

### Crispine Cristian^1^, Luckyrose Mushi^1^, Johnston Mukiza^1^, Happynod Kombe^1^

#### ^1^Kilimanjaro Christian Medical University College, Moshi, Tanzania

##### **Correspondence:** Crispine Cristian (crispinecristian@gmail.com)

*BMC Proceedings 2023,* **17(13):** A99

**Introduction**

Sexually transmitted infections (STIs) prevalence and sexual risk behaviors are high among youth, and knowledge about STIs is still low, especially in developing countries. STIs are important yet less emphasized health problems in Tanzania as the result, there is still a challenge among adolescents in our local settings.

**Objectives**

The study aims to determine the prevalence and factors associated with poor knowledge of STIs among secondary school students in Moshi municipal, Kilimanjaro region, Tanzania.

**Methodology**

Analytical cross -sectional study was conducted in Moshi municipal, Kilimanjaro region in July 2022. Systematic random sampling was used to select study participants. In each school, we assigned numbers to all students who were willing to participate in the study and chose a minimum of 53 students to make a total of 424 students. A total of 423 secondary school students (230 males and 193 females) aged between 11 years to 30 years were surveyed using a standard self-administered questionnaire. Data analysis was done using SPSS version 20.0.

**Results**

Of the 426, 423 students filled the questionnaires making a response rate of 99.3%. Of these, 230 (54.4%) were male and 193 (45.6%) were female. The mean age of the respondents was 17 (SD 1.64) years, ranging from 12 to 30 years. Majority of respondents 42(97.4) reported to have heard of STIs.314 (74.2%) of respondents had poor knowledge about STIs.

**Conclusion**

The proportion of good knowledge of sexually transmitted infections was substantially low therefore strengthening information, education, and communication on the issue using health clubs and mass media is highly recommended. In addition, inculcating reproductive health course in the curriculum plays a paramount importance.

## A100. Determinants of sustained viral load suppression in HIV-infected orphans and vulnerable children on antiretroviral therapy in Tanzania

### Levina Kikoyo^1^

#### ^1^PACT, Dar es Salaam, Tanzania

##### **Correspondence:** Levina Kikoyo (lkikoyo@pactworld.org)

*BMC Proceedings 2023,* **17(13):** A100

**Introduction**

The ultimate target of antiretroviral therapy (ART) is viral load suppression (VLS). While achieving VLS is a vital milestone, it is inadequate if it cannot be sustained, and this is a challenge in Tanzania. This study assessed the level and determinants of sustained VLS among orphans and vulnerable children (OVC) receiving ART in Tanzania.

**Methodology**

Data stems from the USAID KizaziKipya (2016–2021) that aimed to increase the uptake of HIV/AIDS services, and other health and social services by OVC and their caregivers. Clinical records of OVC living with HIV aged 0–18 years were tracked from care and treatment centers. OVC who had been tested twice for viral load (VL) between 2019 and 2021 and were virally suppressed at the first test, were tracked for sustained VLS at the second test. The tests were spaced at least 6 months apart. VLS was defined as viral load <1,000 copies/mL. Sustained VLS was defined as viral load <1,000 copies/mL at both first and 1second tests.

**Results**

Data on 6,016 OVC (52.8% female) aged 0-18 years were analyzed. The median duration between the VL tests was 12.2 months. Sustained VLS was observed in 93.4% of the study population. After adjusting for adherence to ART, age, sex, place of residence, and household economic status, the strongest determinant of sustained VLS was food security, whereby OVC from food secure households were twice more likely to sustain VLS than those from food insecure households (OR=2.02, 95% CI 1.22–3.33). Sustained VLS also varied by geographical location.

**Conclusion**

VLS rate among OVC was 93.4% within a median duration of 12.2 months between VL tests. Results suggest a need to address food insecurity as a critical barrier for sustained VLS among OVC. Geographical areas where VLS was less likely to be sustained should be prioritized with appropriate interventions.

## A101. Extended spectrum beta lactamase producing gram negative bacteria (esbl-gnb) colonizing neonates admitted in the neonatology units at Bugando medical centre, Mwanza-Tanzania

### Jacobo E. Machimu^1^, Vitus Silago^2^

#### ^1^Intern Health Laboratory Scientist at Muhimbili National Hospital, Dar es salaam, Tanzania; ^2^Assistant Research Fellow at Catholic University of Health and Allied Sciences (CUHAS), Mwanza, Tanzania

##### **Correspondence:** Jacobo E. Machimu (machimujacobo@gmail.com)

*BMC Proceedings 2023,* **17(13):** A101

**Introduction and Objectives**

Recent studies at Bugando medical center (BMC) have shown evidences of increased neonatal colonization with Extended-spectrum β-lactamases producing Gram negative bacteria (ESBL-GNB). Reports were shared to the neonatology units where by interventions to minimize and control colonization were initiated and are currently on going. This study aimed to investigate the current prevalence after interventions, antibiotic susceptibility patterns and factors associated with neonatal rectal colonization with ESBL-GNB at BMC, Mwanza-Tanzania.

**Methodology**

This cross-sectional hospital-based study was conducted among 259 neonates admitted in neonatology units at BMC between February and August 2021. Participants’ data were collected using structured questionnaires. A total of 476 neonatal rectal swabs were collected during study period. Swab samples were directly inoculated on MacConkey agar (MCA) plates supplemented with cefotaxime 2μg/ml for screening of ESBL-GNB. Conventional biochemical identification tests, Kirby-Bauer technique and disk combination technique were used for species identification, antibiotic susceptibility testing and phenotypic confirmation of ESBL-GNB production, respectively.

**Results**

Out of 476 rectal swabs, 182 (38.2%) had positive growth on MCA-C of which 19 samples had poly-microbial growth resulting to 201 bacteria isolates. The prevalence of rectal colonization with ESBL-GNB among neonates was 20.08% (54/259). Significantly, the use of nasogastric tubes in situ (OR [95%CI] 1.891 [1 – 3.55], p = 0.048) was associated with ESBL-GNB neonatal colonization. Neonates born by caesarean section (CS) method were not significantly associated with colonization but the risk of becoming colonized was increased by almost 2 times.

**Conclusion and Recommendations**

This study found that prevalence of neonatal colonization with ESBL-GNB (20.03%) at BMC is relatively lower than recent studies (25.4% in 2013 and 54.6% in 2016). Continuous implementation of the initiated interventions to reduce neonatal colonization including antimicrobial stewardship is recommended.

## A102. Factors influencing integration of non-communicable diseases into hiv/aids healthcare services in Tanzania

### Anzibert Rugakingira^1^, Nathanael Sirili^2^, Emmy Meta^2^, Kaushik Rumaiya^3^, Amani Kiondo^2^, Bruno Sunguya^2^

#### ^1^Ministry of Health, Dodoma, Tanzania; ^2^Muhimbili University of Health and Allied Sciences, Dar es salaam, Tanzania; ^3^Shree Hindu Mandal Hospital, Dar es salaam, Tanzania

##### **Correspondence:** Anzibert Rugakingira (ranzibert@gmail.com)

*BMC Proceedings 2023,* **17(13):** A102

**Background**

NCDs are becoming a more common cause of morbidity in LMICs, particularly among PLHIV due to the stimulation of inflammatory markers and ART-associated reactions. Integration of NCDs into HIV/AIDS services has been advocated as a key method for boosting the global HIV response's sustainability and achieving the aim of 'ending AIDS by 2030.' Various studies have been conducted to examine the models, policies, goals, and needs for integration systematically. However, studies on the factors influencing HIV & NCD integration services in Tanzania are limited.

**Objective**: To explore factors influencing the integration of NCDs into HIV/AIDS healthcare services in Tanzania.

**Methodology**

An exploratory qualitative case study was conducted at the Ministry of Health, PORALG, NACP, TACAIDS, and TaNCDP where 22 key informants were interviewed. This study was based on the experiences and decisions of the key informants related to study objectives and will be guided by SII. Thematic analysis was employed for analysis assisted by NVivo software (QSRinternational) version 12. The analysis was carried out in five stages suggested by Virginia Braun and Victoria Clarke.

**Results**

Motivation, positive attitude, awareness, adequate knowledge and skills promote integration, perceived negative attitude and additional workloads to HIV service providers inhibit integration. Adequate human resources and funds were organizational factors for integration but hindered by perceived additional costs, varying donors' interests. Moreover, adequate data collecting tools and diagnostic devices were among health system factors for integration but were limited by perceived additional health infrastructures and the need for reorientation and refresher training.

**Conclusion**

Integration increase access, improving quality, and sustaining NCDs control interventions in HIV/AIDS program.

## A103. Youths’ behaviour and sexually transmitted infections in higher learning institutions: the case of Mbeya University of science and technology, Tanzania.

### Leoncia H. Kibani^1^, Joyce Ndalijanye^1^

#### ^1^Mbeya University of Science and Technology, Mbeya, Tanzania

##### **Correspondence:** Leoncia H. Kibani (lkibani@yahoo.com)

*BMC Proceedings 2023,* **17(13):** A103

**Introduction**

Sexual transmitted infections in particular STIs, RTIs and HIV/AIDS are associated with improper sexual behaviors among victims. Studies on sexual transmitted infections to adolescents are available but little is known on prior researches on youths in higher learning institutions. This qualitative exploratory case study investigated on this area to identify the magnitude of transmission of STIs, RTIs and HIV/AIDS infections among youths to determine why engaging in risk sexual intercourse?

**Methodology**

The study was conducted at Mbeya University of Science and Technology, Tanzania. A total of 300 University students aged between (15-28) years, 50 academic and 20 non-academic teaching staff participated in the study. In practice, pilot study for organizational framework started, followed field work, then data collection and ends with the research questions. Instruments used were questionnaires, interview and focus group discussion guide. Purposive sampling was to University students, random sampling to academic and non-academic staffs. Data were analyzed thematically.

**Results**

Youths (70%) engaged in risk sexual intercourse, (89%) had more than one sexual partners. (72%) acknowledged not using condom. (94%) were familiar with HIV/AIDS but (73%) had sexual intercourse several times with more than one partners. Male youths (64%) admitted that female dressing habits contribute to today’s male reduced sexual power, and (70%) pointed on peer pressure and background residential environment to be main factors for youth’s sexual infections. Other factors mentioned by (80%) non-academic staff and (43%) non-academic staff include temptations and freedom of being away from homes, and possession of unexperienced loan money.

**Conclusion**

Sexual transmitted infections spread faster among youths in higher learning institutions because of varied temptations, freedom and possession of money. It could be better to employ almost the same effort of educating youths on STIs and RTIs as it is for HIV/AIDS. Interventions, seminars and workshops are of paramount.

## A 104. A 30 - year-old man with Marfan syndrome, post-coital coughing and dyspnea

### Fredrick M. Kalokola^1^, DeodatusMabula^1^, Patrick Ngoya^2^, Christopher Mwanansao^3^

#### ^1^Weill Bugando School of Medicine, Mwanza, Tanzania; ^2^Deaprtment of Radiology, Catholic University of Health and Allied Sciences, Mwanza, Tanzania; ^3^Department of Ophthalmology, Bugando Medical Centre, Mwanza, Tanzania

##### **Correspondence:** Fredrick M. Kalokola (kalokola85@gmail.com)

*BMC Proceedings 2023,* **17(13):** A104

**Background**

Congenital heart diseases can often be missed at birth and childhood period depending on the defect and subsequent hemodynamic disturbances. We report a man with Marfan syndrome (MFS) presenting in heart failure.

**Case report**

A 30 year- old man born with skeletal malformations presented in our clinic with post-coital coughing and dyspnea for the past 6 months. He denied history of chest pain and use of any illicit drugs. His mother had died suddenly at the age of 38 years. He had sunken eyes, long extremities with arachnodactyly and hallux valgus. He had a normal pulse and blood pressure with a displace apical impulse with grade three pansystolic murmur. A visual acuity of 6/18 in both eyes and supra- temporal lens subluxation were noted. Chest X-ray revealed cardiomegaly with a bulging ascending aorta silhouette. On trans-thoracic echocardiography, atria and ventricles were dilated including the ascending aorta and aortic root. A moderate mitral and aortic regurgitation were reported. A left ventricular ejection fraction was noted at 27%. Computed tomography of the chest demonstrated an aneurysmal aortic root and ascending aorta. Heart failure was managed with medications and a referral to cardio-thoracic surgeons for aneurysm repair.

**Conclusion**

We report a case of Marfan syndrome (MFS) presenting for the first time with advanced heart failure which was optimally managed by medical therapy. Patients with MFS die prematurely of aortic rupture. Regular imaging to detect and quantify progression of aortic dilation, β-adrenergic receptor antagonist therapy, and prophylactic aortic repair prevents premature death due to aortic dissection, rupture or regurgitation.

*Disclosure statement*: Informed consent to publish this case study and its potentially identifiable information of the patient was obtained from the individual involved. The patient gave explicit permission for the publication of this case report, including any relevant clinical details.

## A105. Improving HIV Positive case identification among key and vulnerable populations (KVP) through the social network strategy (SNS) in Dar-es-salaam

### Mitya M Kenani^1^, Evelyne Lutainulwa^1^, Judith Kipeleka^1,^ Emilia Vallency^1^, Ali Kaduma^1^, Ayoub Kibao^2^

#### ^1^Management and Development for Health, Dar es salaam, Tanzania; ^2^Dar City Council, Dar es salaam, Tanzania

##### **Correspondence:** Mitya M Kenani (mkenani@mdh.or.tz)

*BMC Proceedings 2023,* **17(13):** A105

**Introduction**

Social network strategy is an evidence-based approach, useful in reaching hidden populations at high risk of HIV infection including key and vulnerable populations (KVP) with an HIV prevalence >8.5% while the general population is at 4.7%. Using existing KVP peers, SNS contributed only seven (3%) of the total (250) HIV positive cases identified which was less the expected 10% contribution from this modality in the Period of October to December.

**Methodology**

During mobile outreach activities, we identified KVPs who elicited their networks. We later engaged network recruiters as key informants (KI) who aided reaching elicited network associates. They were provided with ten thousand shillings as transport to reach networks in hotspots, we also provided commercial condoms (Dume) as a motivation to both KIs and network associates. Daily monitoring of SNS elicitation and testing was done daily through WhatsApp groups.

**Results**

Positive identification from SNS modality Increased from only 7 positive cases which was 3% of the total 250 cases identified in October to December 2021, to 102 which is 13% of 760 HIV positive cases identified in the period of April to June 2022. Total KVP positive case identification improved from 250 cases in the period of October to December to 760 cases in the period of April to June.

**Implications**

Engagement of network recruiters as key informants in reaching elicited network associates has improved HIV positive case identification among KVP through the SNS. SNS implementers may consider this strategy in reaching elicited hard to reach networks with HIV testing services that will help in HIV case identification among KVPs.

## A106. “Kwa tatu bomba, watachanja” initiative in reaching more clients for covid 19 vaccination in the community, afyajumuishi project – Geita

### Daniel C. Mpembeni^1^, Florian Mutasingwa^1^, Nicholaus Tarimo^1^

#### ^1^Management and Development for health, Dar es salaam, Tanzania

##### **Correspondence:** Daniel C. Mpembeni (dmpembeni@mdh.or.tz)

*BMC Proceedings 2023,* **17(13):** A106

**Introduction**

COVID 19 disease caused high morbidity and mortality across countries, about 36,520 infections and 841 deaths were reported by June 2022 in Tanzania. In December 2020, WHO issued guidance of vaccinating 70% of the eligible population including 100% of elderly population (60+yrs). By May 2022, 21% of eligible clients were vaccinated in Geita less than expected target. Vaccination efforts were rejected due to low awareness, knowledge and negative myths.

**Methodology**

In June 2022, MDH collaborated with the government to conduct 7 days intensified vaccination efforts in the community through joint efforts of healthcare workers, community health workers and ten cell leaders to ensure eligible clients are reached for COVID 19 vaccination, initiative termed “Kwa TatuBomba, Watachanja”. The initiative facilitated door to door vaccination outreach services in more households, reaching clients in congregated settings (markets, football grounds, churches, mosques) to reach more clients for sensitization, mobilization and vaccination.

**Results**

The initiative led to formation of 514 teams in 170 facilities that provide vaccination services, 257,188 clients were vaccinated in 7 days which includes 32,829 elders. This has been the highest number of clients vaccinated in 7 days, compared to the highest reached before the initiative, which was 51,263 clients. In addition, 45% of the eligible population was vaccinated, which is an increment of 24% as compared before initiative. The communities were sensitized on COVID 19 vaccination and myths were easily cleared as vaccination teams had enough time to discuss with clients and sort out issues.

**Implication**

“Kwa TatuBomba, Watachanja” initiative increased COVID 19 vaccination uptake and led to positive attitudes towards COVID-19 vaccine, PO-RALG should advocate for similar interventions elsewhere. Daily monitoring of COVID 19 vaccination data and vaccine stock issues is key to enable timely sorting out of issues raised during the activity.

## A107. Former tb clients, a game changer to improve tb presumptive cases among plwhiv, a case study Biharamulo Dc-Kagera

### Frank C. Malugu^1^, Mary Temu^1^, Edgar Mpfubusta^1^, Emmanuel Sarakikya^1^, Michael Kasmir^1^, Isaac Laizer^1^, Jonathani Katemi^1^

#### ^1^Management and Development for Health, Dar es salaam, Tanzania

##### **Correspondence:** Frank C. Malugu (fmalugu@mdh.or.tz)

*BMC Proceedings 2023,* **17(13):** A107

**Introduction**

The World Health Organization (WHO) advises if TB screening is carried properly, between 10 and 20% of PLHIV will be reported as presumptive cases with at least one symptom suggestive of TB.

However, Biharamulo had low TB presumptive cases with rate of 2% by (Oct2021-Dec2021). Suboptimal screening from health care providers was one of the challenges. Engagement of TB former clients on TB screening was a strategy taken.

**Methodology**

MDH in Kagera through CDC supports the government on TB/HIV interventions.

TB unit came with a strategy which is Client centred approach by Engaging of former TB clients, Training was conducted and capacitated five former TB clients on TB package on TB screening signs and symptoms and attached them to five out of 18 supported CTC sites.

**Results**

Engaging of TB former clients for TB screening among PLHIV has been a game changer and has brought great results to improve TB presumptive cases among PLHIV.

Furthermore, this strategy led to increase TBHIV cases notification, before intervention TBHIV case were 10 clients but after intervention improved to 30 TBHIV cases. Of which 26 cases were results of 5 out of 18 sites which were included. This strategy is advised to be scaled up to other areas implementing TB/HIV interventions.

**Conclusion**

The trained TB former clients strengthened TB screening at five the health facilities which they were located presumptive cases magnificently improved among PLHIV improved, health education on TB signs and symptoms was the package supplied during each CTC visit from former TB clients to all PLHIV attending CTC.TB presumptive cases improved which lead to improvement of the overall council TB presumptive rate from 2%(Oct 2021-Dec 2021) to 11%(Jan 2022-March 2022).

## A108. Peer involvement in accelerating cervical cancer screening among women living with hiv on antiretroviral in Tabora region

### Christian Paresso^1^, Gladson Koshuma^1^, Rahel Hhawu^1^, Lucy Magesa^1^, Michael Mahande^1^, Happiness Viso^1^, Adam Mrisho^1^, Chagi Madogo^1^

#### ^1^Management and Development for Health, Dar es salaam, Tanzania

##### **Correspondence:** Christian Paresso (cparesso@mdh.or.tz)

*BMC Proceedings 2023,* **17(13):** A108

**Introduction**

Globally, cervical cancer is the fourth most common cancer among women. Women living with HIV (WLHIV) have higher risk of cervical cancer compared to women without. Cervical cancer is preventable and curable if detected early. It’s recommended at least 80% WLHIV undergo cervical cancer screening (CCS) annually, however in Tabora before the year 2020, about 10% of WLHIV received CCS annually. Inadequate trained providers contributed to low uptake of CCS.

**Methodology**

On job training and mentorships on CCS were done to 60 health care providers and 60 Peers (WLHIV previously screened and treated) from 50 high volume sites and screening supplies were provided. Peers provided health education and sensitization during clinic visits. Management of client flow at clinics was done to capture all eligible WLHIV. Outreach services were scheduled to sites with no trained providers every month.

**Results**

Before the year 2020 less than 1000 WLHIV received CCS annually with no peers involved. For the period of the years 2020-2021, about 40,000 WLHIV out of 41,367 on antiretroviral, (above than 90%) in Tabora region were screened for cervical cancer, with an average of 20,000 screened per year. About 60 health care providers and peers have been trained to provide CCS across the region.

**Conclusion**

On job training and use of peers in demand creation play a major role in accelerating cervical cancer screening and linkage to treatment services. We therefore recommend CCS programs to adopt on job training and use of peers in demand creation so as to accelerate cervical cancer screening and linkage to treatment services.

## A109. Experiences of family caregivers in caring for patients with heart failure At Jakaya Kikwete Cardiac Institute, Dar Es Salaam, Tanzania

### Tunzo L. Mcharo^1^, Edith Tarimo^2^, Masunga Iseselo^2^, Samwel Kahema^3^

#### ^1^Jakaya Kikwete Cardiac Institute, Muhimbili National Hospital, Dar es salaam, Tanzania; ^2^Muhimbili University of Health and Allied Sciences, Dar es salaam, Tanzania; ^3^Morogoro College of Health and Allied Sciences, Morogoro, Tanzania

##### **Correspondence:** Tunzo L. Mcharo (tuzolundgreen@gmail.com)

*BMC Proceedings 2023,* **17(13):** A109

**Background**

Introduction: Heart failure (HF) is still a major global health issue, and the low-income countries are particularly affected. A patient with HF requires continued medical care as well as careful supervision from family members who keep an eye on the patient's prognosis, medication compliance, and assistance with everyday activities.

Objective: To explore the experiences of family caregivers in caring for patients with HF.

**Methodology**

Methods and materials: A descriptive exploratory study design using a qualitative approach was conducted at Jakaya Kikwete Cardiac Institute (JKCI), Dar es Salaam, Tanzania. A purposive sampling technique was used to select the potential participants. A sample size of 10 caregivers of patients with HF was included in the study based on the principles of data saturation. Thematic analysis was used to derive the main theme and sub-themes.

**Results**

Three major themes were identified: Needs of caregivers of patients with HF, challenges to new roles and lifestyles, and professional support in caring for patients with HF. Caregivers struggled with caring responsibility while facing economic hardship, lack of a supportive environment in hospital, Also, providing appropriate care to patients with HF was difficult due to financial constraints, inadequate social services in the hospital setting, and lack of time to participate in community activities among the caregivers. However, caregivers reported to receive support from nurses which contributed to the positive experiences in caring of the patients with HF.

**Conclusion**

Caring for patients with HF requires physical, economic, social, and emotional support to improve quality of life to both patients and caregivers. Further studies should be conducted to assess nurses' perceptions on the importance of cooperating with caregivers in caring for patients with HF.

## A110. Rafiki wa dhahabu as a catalyst to HIV testing among priority population in mining areas: A case of Buseresere dispensary

### Elizabeth H. Lyimo^1^, Rosemary Khamsini^1^, David Kikuli^1^, Ally Mawanja^1^, Daniel Mpembeni^1^, Frank Msangi^1^, Dina Gerald^1^

#### ^1^Management and development for Health, Dar es salaam, Tanzania

##### **Correspondence:** Elizabeth H. Lyimo (drelizabeth.herman@gmail.com)

*BMC Proceedings 2023,* **17(13):** A110

**Introduction**

Despite progress in scaling up HIV Testing in Geita Region, HIV Testing and prevention among miners is suboptimal. Miners being among Priority Population with high prevalence of HIV infection, a migrant community with working hours which do not align well with HIV Testing compared to the general population making it difficult to reach them. MDH in collaboration with R/CHMT incorporated Rafiki wa Dhahabu a client centered approach to reach them.

**Methodology**

Between January and June 2022, were able to leverage the existing Social Network Testing through the initiative Rafiki wa Dhahabu that simplified reaching priority population in mining sites. HIV-positive and HIV-negative miners at high risk for HIV (recruiters) were enlisted to identify and recruit persons from networks (network associates) for HIV testing. Miners were trained to act as peers and key informant, sensitized to mobilize, facilitate HIV Testing, formation of groups to enhance provision of health education and prevention services.

**Results**

Between October 2021 and January2022 we recruited 8 key informant miners at Buseresere dispensary in Chato District who also acted as peers, demand creators and informed the HCPs on the suitable time for HIV testing at the mining sites. After initiation of Rafiki wa Dhahabu approach we were able to scale up the number of miners reached from 60 in January 2022 to 661 by June 2022 who received HIV Testing Services and Prevention Services. Among these 23 clients were newly diagnosed with HIV/AIDS and linked to care and 593 provided with prevention services like PrEP and condoms.

**Implication**

Implementation of Rafiki wa Dhahabu has increased the number of miners reached for HIV testing services by tenfold and contributed highly in attaining the annual target. Scaling up this initiative to more wards around mining sites and other migrants’ communities like fishers will increase testing coverage among priority populations.

## A111. Impact of focused hiv testing services on improving hiv case identification in Dar es Salaam

### Abdallah S. Mfaume^1^, David Sando^1^, Yusuph Chende^1^, Ally Ramadhani^1^ Kaduma^1^, Goodluck Lyatuu^1^, Kasasi Fred Mayogu^1^, Shwaib Yusuf Ojunju^1^, Linda Ernest Nkaka^1^, Salim Jarib Sultan^1^

#### ^1^Management And Development for Health, Dar es salaam, Tanzania

##### **Correspondence:** Abdallah S. Mfaume (amfaume@mdh.or.tz)

*BMC Proceedings 2023,* **17(13):** A111

**Introduction**

Dar region is estimated to have 255,000 people living with HIV/AIDS, PLHIV whereby only 83% (211,650) of PLHIV were identified through routine HIV testing strategies. In bridging the gap of low HIV identification; MDH in collaboration with the government introduced focused testing of HIV in all supported facilities. Focused testing for HIV involves Index testing which is HIV testing to all contacts of the Index clients; and Optimized Provider Initiated Testing, HIV testing after screening for all clients attending at outpatient department in all hospital settings.

**Methodology**

MDH in collaboration with R/CHMTs identified optimized PITC and index testing as a new strategy for focused HIV testing.

During focused HIV testing; HIV specific testers and expert clients/peers were hired and trained to document contacts of all HIV clients who are on care services within facilities and were contacted for HIV testing. While in optimized PITC, all clients attending at outpatient departments were screened for eligibility to test for HIV.

Performance is monitored through daily, weekly and monthly reports for monitoring and evaluation.

**Results**

Total of 6,926 POS were identified in the period of October 202 to March 2022; Index testing services contributed to 34% (2,476 PLHIV) while Optimized Provider Initiated Testing service contributed 43.7% (2,994 PLHIV). This marks 77.7% results from focused HIV testing services.

**Implication**

Both HIV specific testers and expert clients/peers need to be capacitated on proper documentation of the tools that are used during services. Expert clients/peers need more knowledge on proper screening of clients for HIV testing eligibility. All contacted clients from care services and at outpatient departments showed high acceptance on the initiatives and gained more knowledge on importance of HIV testing services.

Focused HIV testing is an effective means for HIV case identification as it reduces massive testing and wastage of resources

There is a need for continuous onsite mentorships to HIV specific testers and expert clients/peers for sustained services.

This initiative can be scaled up to all facilities with better performance outcomes.

## A112. Challenges and insights from engaging male partners of young first-time mothers

### Lilian Kapinga^1^, Jennifer Seager^2^, Melanie Yahner^3^, Emma Cook^2^, Meroji Sebany^3^,Sarah Elaraby^3^

#### ^1^Save the Children International, Dar es salaam, Tanzania; ^2^George Washington University, Washington, USA; ^3^Save the Children US, Washington, USA

##### **Correspondence:** Lilian Kapinga (Lilian.Kapinga@savethechildren.org)

*BMC Proceedings 2023,* **17(13):** A112

**Introduction**

Despite male partners’ roles in household health practices and decisions, engaging men remains challenging. The transition to becoming a parent for the first time provides opportunities to transform gender norms and support adoption of healthy behaviors, couple communication and decision-making on use of postpartum family planning (PPFP) for healthy timing and spacing of pregnancies. This study aimed at identifying challenges and insights from engaging male partners of young first-time mothers.

**Methodology**

Save the Children (SC) supported community health workers (CHW) to reach FTMs with PPFP counseling, referrals, and methods through home visits. Male partners were engaged in home visits when available. SC conducted implementation research to identify needed refinements to approaches (key informant interviews with 62 FTMs and 20 partners). Quantitative surveys of 351 FTMs assessed PPFP uptake, fertility preferences, communication, and decision-making.

**Results**

Only 43.9% of FTMs reported that partners wanted the same family size; 30.6% did not know partners’ preferences. Most FTMs reported that partners made final fertility-related decisions. Implementation learning identified limited partner participation in visits, although partners expressed interest in PPFP. Men rarely participated in visits, but asserted desire to make decisions. Some FTMs emphasized they needed partners’ approval to access PPFP; others hesitated to engage partners, preferring to use PPFP secretly. Some efforts to encourage male partner engagement, through formal policies or individual providers requiring partner accompaniment, had unintended consequences discouraging service use.

**Conclusion**

The findings underscore the need for strengthened couple communication, but FTMs expressed mixed preferences on involving their partners. Further discussion is needed around whether under certain circumstances, engaging men may impede women’s access to services or autonomy to make FP decisions on their own behalf.

## A113. Assessment of knowledge, attitudes and practices toward prevention of hepatitis b virus infection among secondary students above 18 years old at Benjamin W. Mkapa high school in Dar Es Salaam, Tanzania

### Hamis M. Isonda^1^, Elizabeth Popova^1^

#### ^1^St Joseph College of Health and Allied Sciences, Dar es salaam, Tanzania

##### **Correspondence:** Hamis M. Isonda (isonda83@gmail.com)

*BMC Proceedings 2023,* **17(13):** A113

**Introduction**

Hepatitis B is a potentially life-threatening liver infection caused by hepatitis B virus (HBV). It’s a global burden infecting 240 million worldwide. It causes chronic inflammation leading to cirrhosis and liver carcinoma. It’s transmitted either in horizontal or vertical transmission. We aimed the assessment of knowledge, attitude, and practices towards prevention of hepatitis B among secondary students at Benjamin W. Mkapa high school in Dar es salaam, Tanzania.

**Methodology**

In a cross-sectional study involved 100 advanced level students above 18 years both girls and boys. Data analyzed by using SPSS 25 version software. Ethical Clearance for the Study requested from the Institution review board of St. Joseph College of health and allied sciences (STJCHAS-IRS) before the study begins and administrative permission requested from Ilala Municipal Council, secondary education department and Benjamin W. Mkapa high school headmaster.

**Results**

A total of 100 respondents were included in this study which making 100% response rate. 56 respondents were of 18 years old 44 respondents were of 19 years make a (44%). 33% of the respondents had good level of awareness, 20% of respondents had average level of awareness and 47% had poor awareness. Out of the total respondents 30% had good attitude,27 % had average attitude and only 43% had poor attitude on the prevention of HBV vaccination.

**Conclusion**

The study revealed the gap of knowledge about the viral infection causes, modes of transmission, modes of prevention. Those data require to consider in health education.

## A114. HIV patients’ retention and attrition in care: a review of documented reasons for 12 months post-enrollment among newly enrolled people living with HIV in Tabora-Tanzania

### Beatrice Praise^1^, Josephat Magawa^1^, Sheila Kisure^1^, Ramadhani Shemtandulo^1^, Ibrahim Seba^1^, Amani Shao^1^, Adam Mrisho^1^, lilian Lema^1^, Vincent Ngelageza^1^

#### ^1^Management and Development for Health, Dar es salaam, Tanzania

##### **Correspondence:** Beatrice Praise (beatricepraise@hotmail.com)

*BMC Proceedings 2023,* **17(13):** A114

**Introduction**

Tanzania adopted the standard (95-95-95) and currently 88% of people living with HIV (PLHIV) know their status,98% on ART, and 96% are virally suppressed. Despite this progress, retention in care 12 months post enrollment remains a challenge. Attrition from care leads to poor health outcomes and HIV transmission. We aimed to review documented reasons for retention and attrition from care 12 months post enrollment to inform program implementation.

**Methodology**

We conducted a review of secondary data from CTC2 database from 151 health care facilities for documented reasons for retention and attrition 12-months post enrollment of all newly enrolled clients at the end of June 2022. Sociodemographic, clinical data and attending status were extracted and descriptive analysis was conducted using to identify magnitude for documented reasons for retention and attrition in care. Attrition from care was defined as either people who died, opted out, transferred out or missed appointment.

**Results**

Total of 14,544 clients were enrolled during the analysis period. Majority of the clients were women 8647(59%). Most of the clients, 8283(56.9%) were aged between 20 to 35 years and more than 80% were from rural areas. Out of the total clients, 2653 (18.4%) were not retained in HIV care and treatment services. Documented reasons for attrition from HIV care and treatment were being transferred out 1305(49.2%, followed by deaths 685(25.8%), interruption in treatment 378(14.2%), missing appointment 190(7.2% and opted out 95(3.6%).

**Implication**

Program implementers need to address document reasons for attrition such as transfer out, deaths, interruption in treatment, missing appointments and opting out of care sustainably. Specifically, addressing treatment interruption may lead in to reduction in avoidable deaths, which in turn may improve retention in to HIV care and treatment.

## A115. Documented causes of deaths among people living with hiv/aids: evidence from desk review of programmatic data in Tabora region-Tanzania

### Ramadhani Shemtandulo^1^, Beatrice Praise^1^, Amani Shao^1^, Faustine Polycarp^1^, Adam Mrisho^1^, Samweli Nymbalino^1^, Jane Joseph^1^, Stephen Ambonisye^1^, Said Hussein^1^

#### ^1^Management and Development for Health (MDH), Dar es salaam, Tanzania

##### **Correspondence:** Ramadhani Shemtandulo (rshemtandulo@mdh.or.tz)

*BMC Proceedings 2023,* **17(13):** A115

**Background**

About 670000 [500 000–970 000] people died from HIV/AIDS-related illnesses worldwide with more than one third being from sub-Saharan Africa. In Tanzania, with HIV prevalence of 4.7%, it is estimated 24,000 deaths every year are HIV related. However, documentation of these causes of these deaths is poorly understood. We aimed to review client’s files to establish the documented causes of deaths among PLHIV taking antiretroviral treatment in Tabora Region.

**Methodology**

This is a desk review, whereby client’s files of PLHIV death occurred from October 21 to March 22 from 151 supported health facilities in Tabora were reviewed and client’s demographic information and documented cause of death were extracted from client’s clinical files. Data were cleaned and analysed using STATA version 17. Descriptive analysis was conducted to determine the frequency and percentage of cause of death as it has been documented from client’s files.

**Results**

Out of 1150 files reviewed, 728 (63.3%) had documented death information. Fifty percent were males. The median age was 40 (IQR 32-50) years. The review showed, that 291(40%) of deaths were due to HIV related disease including Tuberculosis. Tuberculosis alone contributes 117 (16.1%) of the deaths while 146(20%) were due to infections such as malaria and typhoid fever. In addition,17% of the deaths were due to natural causes such as non-communicable disease while 109(15%) of the deaths were due to non-natural causes such as accidents.

**Conclusion**

These findings suggest that addressing HIV related documented causes of deaths can avert majority of the HIV related deaths such as TB. There is a need for training clinicians at health care facilities on documentation of causes of death as majority of files had no documentation of cause of deaths.

## A116. Prevalence and factors associated with opioid abuse among mental health patients at Mwananyamala hospital, Dar Es Salaam

### Lucas Alfred^1^, Isaya Mhando^1^

#### ^1^St Joseph College of Health Science, Dar es salaam, Tanzania

##### **Correspondence:** Lucas Alfred (lucasalfred161@gmail.com)

*BMC Proceedings 2023,* **17(13):** A116

**Background**

New estimates for Asia and Africa have contributed to the most significant increase in the global number of opioid users in the recent years. The increased prevalence of opioid misuse, use disorder in recent years has highlighted the need for improved public health approaches for reducing the tremendous harms of this illness.

**Methodology**

A cross-sectional study was conducted at Mwananyamala Hospital, Kinondoni District, Dar Es Salaam. 207 mental health individuals were recruited in the study. Using questionnaire, determination of the prevalence of opioid abuse was done. Data analysis was done using spss version 20. Where opioid abuse was cross tabulated in chi-square tes against social demographic factors at p value < 0.05.

**Results**

This study recruited 207,most of the respondents were male (91.8%) and female (8.2%). Majority of the respondents were married (55.1%), single (31.4%), separated/divorced (13%) and widow/widowed (0.5%). Primary education (65.7%), secondary education (30.9%), college/university (2.4%) and 1% never attended, the prevalence of opioid abuse among mental health patients attending at Mwananyamala Hospital was 17.9%. Lower economic status, low self-esteem, availability of the drug, depression, desperation, lack of sleep and chronic illness were associated with the opioid abuse.

**Conclusion**

This study reports a high prevalence of opioid abuse in individuals with mental illness attending at Mwananyamala Hospital. Establishment of community mental health facilities and substance rehabilitation centers is important to reduce substance abuse.

## A117. Contribution of DHFF toward improving provision of health services in the primary health facilities: A case of Gairo DC

### Dr Dastan Mshana^1^, Lawrencia Mushi^1^

#### ^1^Mzumbe University, Morogoro, Tanzania

##### **Correspondence:** Dastan Mshana

*BMC Proceedings 2023,* **17(13):** A117

**Background**

The study was aimed at evaluating contribution of Direct Health Facility Financing (DHFF) towards improving provision of health services in health facilities. This overall objective was guided by the specific objectives which were: To determine the contribution of DHFF on availability of essential medicine and medical supplies, improvement of health infrastructures, payment of workers statutory benefits and increase accountability and transparency of health facilities.

**Methodology**

Case study design was used to conduct this evaluation, and Gairo district council was the area of the study. A total 83 respondents including health providers and community members were involved in health facility governing committee was involved. Purposive and convenience sampling techniques were used to select study sample. Questionnaire and in-depth interview were the methods for data collection. Descriptive statistics was used to analyze through Microsoft excel while qualitative data used phenomenological and descriptive analysis through Atlas Ti software to analyze data collected through in-depth interview. Also content analysis used to analyze data collected from observation.

**Results**

Findings indicate that, Direct Health Facility Financing led to the improvement of provision of health services in health facilities. Specifically, it increased availability of tracer and essential medicine by 90.1% in 2017, 91.8% in 2018, 92.9% in 2019 and 95% in 2020, different from 87.1% in 2016 before the onset of Direct Health Facility Financing. Direct Health Facility Financing also led to improvement of health infrastructure by construction and renovation of incinerator, placenta pits, maintenance of machines and equipment through local contractors, payment of staff’s statutory benefits such as call, extra duty and uniform allowances and improved accountability to community. Also the study found delaying of payments process caused by prolonged process.

**Conclusion**

The study recommends for the government to amend financial memorandum to allow head of health department to act as an accounting officer to reduce prolonged process. There is also a need to assess the ability of local contractor in doing constructional assignment and the need for the government to consider marginalized facilities which serve population that involves poorer, most vulnerable groups at high risks.

## A118. The role of group discussion in improving quality of services among PLHIV and appointment keeping at Nyarugusu H.C; Afya Jumuishi Project Geita

### Shukrani Mbwaga^1^, Irene Andrew^1^, Emelda Herman^1^, Talumba Samatta^1^, Nicholous Tarimo^1^, Peter Nyiraha^1^

#### ^1^Management and Development for health, Dar es salaam, Tanzania

##### **Correspondence:** Shukrani Mbwaga (smbwaga@mdh.or.tz)

*BMC Proceedings 2023,* **17(13):** A118

**Introduction**

In addressing the UNAIDS second 95 goal, CDC recommends an appointment adherence of not less than 98%. Nyarugusu H. C experienced a Monthly decrease from February 2022 (97.25%) through May 2022 (95.2%), impacting clients’ retention, increased morbidity and mortality. HCWs did not understand well the reasons for the drop. MDH in collaboration with HCWs conducted group discussion with clients in exploring reasons for missing appointments and ways to improve appointment keeping.

**Methodology**

A group of 12 PLHIVs among 38 tracked back (31% of total returned) whereby; 8 Males and 4 Females from different locations were involved in a joint open-minded discussion to determine factors for missing appointments and opinions to prevent re-occurrence. Three major reasons were listed; clients being served without considering the order of arrival at the clinic, lack of fare to attend the scheduled clinic dates, and appointments mismatch between the CTC1 and 2 cards.

**Results**

Improved appointment keeping among clients from 95.2% in May to 98.4% in June 2022. This was a result of establishment of attendance serial numbers in serving clients that considered the order of arrival at the clinic that is first to arrive first to be served, improved appointment dates alignment between CTC1 and CTC2 cards at the exit desk using exit desk assessment tool, and establishment of three new facility led community outreach refill clinics to serve long distant clients.

**Implication**

This approach helped health care providers to rescue the situation through reaching the clients that could continue to miss their appointments due to service unfriendliness at the facility. Therefore, the engagement of clients in addressing challenges is very vital for the provision of quality client-centered services. We recommend the scale up the practice to other facilities to help address site-specific reasons for improving clients’ quality of services.

## A119. Health care linked coupons for hiv testing and counselling among key and vulnerable populations: lesson learned from Tabora region

### Angelina E. Mchome^1^, Adam Mrisho^1^, Amani Shao^1^, Rachel Kawezya^1^, Catherine Kigaigai^1^, Chagi Madogo^1^, Abelabela Rumisha^1^, Christian Paresso^1^, Bellansila Lebero^1^

#### ^1^Management and Development for Health, Dar es salaam, Tanzania

##### **Correspondence:** Angelina E. Mchome (amchome@mdh.or.tz)

*BMC Proceedings 2023,* **17(13):** A119

**Background**

The global HIV prevalence among key and vulnerable population (KVP) ranges at 9%- 23%, while in Tanzania the prevalence is 26%, slightly above the global range. Despite the efforts to reach KVP for HIV testing service (HTS), HIV prevalence is higher compared to general population. This call for the use of different strategies for reaching KVP. We demonstrate the use of health facility linked coupons in improving KVP- HTS.

**Methodology**

In February 2022, MDH in collaboration with regional and council management teams (R/CHMT) designed and introduced coupon linked with health care sites for improving HIV testing services (HTS) among KVPs. The health care facility linked coupons had provider names and mobile numbers. KVP peers and expert clients received 2-5 coupons for distribution to their friends that share the same behaviors in the community or health care facilities respectively. Upon arrival with health care facility linked coupons, clients received subsidized services.

**Results**

Since inception of coupons distribution in social network strategy between February and June 2022, 20 KVP peers’ volunteer were selected in 10 wards at the community and 20 community-based HIV/AIDS providers (CBHSPs) were selected at 10 high volume facilities in outpatient department (OPD) and at care and treatment clinic (CTC). A total of 607 coupons were distributed, 423(70%) were tested for HIV and 31(7%) clients tested positive and initiated on antiretroviral treatment (ART). While at the community, 603 coupons were distributed, (83%) 501 were tested for HIV and 51(10%) clients tested positive and initiated on ART.

**Conclusion**

We learned that using health care linked coupons, through Social Networks Strategy offered an opportunity to reach KVP for HIV testing services and link them in HIV care and treatment services that would otherwise not be reached. Having facility, ward, village name and HCW contacts facilitated the services provision.

## A120. Improving early infant diagnosis uptake during under five immunization clinics in Kagera region

### Alice Kenneth Simwinga^1^, Phiason Anatory^1^, Fadhili Nyagawa^1^, Phoebe Magatti^1^, Brenda Simba^1^ Loise Odero^1^Theodora Mbunda^1^, Neema Kyamba^1^

#### ^1^Management and Development for Health, Dar es salaam, Tanzania

##### **Correspondence:** Alice Kenneth Simwinga (asimwinga@mdh.or.tz)

*BMC Proceedings 2023,* **17(13):** A120

**Introduction**

Ministry of Health recommends Dried Blood Sample (DBS) for Early Infant Diagnosis (EID) and first dose immunization to be done at 6 weeks of age. Between 2019 -2020, it was noted that during immunization visits more than 30% HIV exposed infants were not reached for DBS, largely due to provider high workload and client’s knowledge gap. Interventions were needed to improve EID uptake among HIV exposed infants on immunization day.

**Methodology**

From January 2020, we equipped labour ward health care workers to pass key information to HIV positive mothers after delivery. Mothers were informed to bring their six weeks infants on the same day for DBS collection and first immunization and informed to remind nurses at RCH for DBS sample collection. At RCH, we trained nurses to screen for HIV status using RCH cards, track eligible HIV exposed infants, effectively link mother-infant pair to DBS collection and promote male partner involvement.

**Results**

EID coverage improved from 53% in January-March 2019 to 100% in January- March 2022. Maternal HIV status screening using RCH cards increased to 97% and reduced number of missed opportunities infants for DBS test. Same day DBS blood test and first dose of immunization demand from HIV positive mothers increased, and more than 95% HIV exposed infants were linked to DBS test on the same day of first dose of immunization at the age of six weeks. On job trained health care workers both from Labor and delivery and reproductive and Child health departments were able to share progress weekly.

**Implications**

Involvement of labor ward staff to promote EID uptake was key to improving EID collection and provide clearly defined instructions to HIV positive mothers post-delivery and provider skills, client’s awareness and acceptance of EID services. This in turn improves the contributing to reduction of mother to child transmission of HIV.

## A121. TB tuwafike zaidi: Reaching out the unreached to end TB through accredited drug dispensing outlets (ADDOs) in Tanzania

### Rose Marwa^1^, Sylivester Magobo^1^, Josephine Balati^1^, Levina Mwesiga^1^, Catherine Makoye^1^

#### ^1^Christian Social Services Commission, Dar es salaam, Tanzania

##### **Correspondence:** Rose Marwa (rmarwa10@gmail.com)

*BMC Proceedings 2023,* **17(13):** A121

**Introduction**

Sustainable full coverage of Tuberculosis (TB) services provision remains a major challenge especially in rural and under-served populations. Reaching out to the unreached to end TB through Accredited drug dispensing outlets (ADDOs) was one of the interventions in improving TB case finding in the community through TB TUWAFIKIE ZAIDI Project intervention in 8 regions of Tanzania.

**Methodology**

The project was conducted from October 2020 to May 2022. The primary intervention was engagement and capacitating ADDOs to screen clients for TB symptoms and to collect sputum from TB presumptive clients and transport to gene expert sites for diagnosis by Sputum Transporters or CHWs. Lab results were communicated back to the ADDOs/CHWs and Confirmed TB+ clients were tracked and accompanied by the CHW to the nearest DOT center for treatment initiation and Contact tracing was done to their families.

**Results**

During the project implementation period, a total of 673,644 people were screened for TB by ADDOs, whereas males were more screened than females by 52%. TB presumptive were 10265 (1.52%) out of total screened whereby only 9933 (97%) were tested. Bacteriological confirmed TB patients were 899 (9%) while All Forms of TB (AF) were 1618 (16%) and 99% among AF were linked by CHWs to Health facilities for TB treatment initiation.

**Implication**

ADDO intervention in addition to other existing community interventions like Traditional healer’s involvement and Contact tracing is essential to improve TB case notification in the community as well as reaching out the clients who do not seek health services at health facilities timely to prevent further complications of late diagnosed TB.

## A122. Enteric pathogens detected from children under five years admitted with diarrhea in Moshi-Kilimanjaro, Tanzania

### Ephrasia Hugho^1,2^, Grace Kinabo^2^, Prof Blandina Mmbaga^1^, Happiness Kumburu^1^

#### ^1^Kilimanjaro Clinical Research Institute, Moshi, Tanzania; ^2^Kilimanjaro Christian Medical University College, Moshi, Tanzania

##### **Correspondence:** Ephrasia Hugho (ephrasiah@gmail.com)

*BMC Proceedings 2023,* **17(13):** A122

**Introduction**

Globally, diarrhea is among the leading cause of mortality among children aged under five years; leading to 125000 deaths every year. Despite the availability and wide c overage of rotavirus vaccination in Tanzania still there is reasonably number of diarrhea cases been reported, some requiring admission. The aim of this study was to investigate diarrhea causing pathogens by using molecular technique.

**Methodology**

A cross sectional study was conducted among hospitalized children (0-59 months) admitted in health facilities in Moshi, Kilimanjaro. Stool samples were collected within 24 hours post admission. Total nucleic acids was extracted using QiaAmp Fast stool kit (Qiagen, Hilden, Germany) and pathogen detection was performed by polymerase chain reaction using TaqMan array card. Amplifications with ≤35 quantification cycles indicated positive pathogen target. Fisher exact test and Chi squared tests were used to compare proportion of pathogen detection across different exposures.

**Results**

Of 146 children enrolled, 56.85% were from rural areas of Moshi district. Vomiting (88.36%) and fever (60.27%) were the common clinical signs. The most prevalent pathogens detected were Enteroaggregative Escherichia coli (EAEC) 53.42% (n= 78), rotavirus 38.36% (n=56), enteropathogenic Escherichia coli (EPEC-eae) 30.82% (n=45), cryptosporidium 21.23% (n=31), Adenovirus-40/41 19.86% (n=29), Norovirus GII 14.38% (n=21), Shigella EIEC 15.07% (n=22), EPEC-bfpA 12.33% (n=18),

**Conclusion**

Diarrhea among under five children is caused by multiple pathogens. Encouraging hand washing hygiene, sanitation, safe drinking water and immunization are the key intervention. However, efforts are needed to investigate other pathogens originating from the environment that are not covered in immunization schemes, yet potential in childhood diarrhea.

## A123. Couple disagreement in fertility desire and its association with contraceptive use in Magu districts, Mwanza region, Tanzania

### Henry L. Mlay^1^, Denna Michael^3^, Innocent B Mboya^1^, Michael Ngowi^1^, Mark Urassa^3^, Gilbert Waria^1^, Jim Todd^2^

#### ^1^Kilimanjaro Christian Medical University Collage, Moshi, Tanzania; ^2^London school of hygiene and tropical medicine, London, UK; ^3^National Institute of medical research (NIMR), Mwanza, Tanzania

##### **Correspondence:** Henry L. Mlay (henrymlay54@gmail.com)

*BMC Proceedings 2023,* **17(13):** A123

**Introduction**

Countries in sub-Saharan Africa (SSA) have some of the highest fertility rates, accompanied by lowest contraceptive use and a higher unmet need for contraceptives. In Tanzania, the use of contraceptives is still low. It is unclear how family planning decisions are reached and what influences them. Factors such as disagreement in fertility desire within sexual partnerships and their association with contraceptive use are yet to be explored. The study aimed to determine theassociation between couple disagreement in fertility desire and its association with contraceptive use.

**Methodology**

A cross-sectional study was conducted among 355 couples who participated in the sero-survey in 2016 within the Kisesa open cohort. Disagreement in fertility desire occurred when the husband reported desire for a child but the wife reported not to desire and when the wife desired but the husband did not. Stata software 15 was used for data analysis. Multilevel Poisson regression model was used to determine the association between couple's disagreement in fertility desire and contraceptive use

**Results**

A total of 355 couples were included in the study. Among these couples, 28.5% had a disagreement in fertility desire. contraceptive use in the disagreed couples was 23.1% among couples in which only the husband desired a child but the wife did not. Lower prevalence of contraceptive use was among couples in which both partners desired children. Higher contraceptive use prevalence was among couples where only the husband desired children compared to when both did not. The association between couples disagreement in fertility desire with contraceptive use was not statistically significant.

**Conclusion**

Couples' disagreement in fertility desire was not significantly associated with contraceptive use, Confounders such as religion, couple's age difference and couple's intention to use family planning were significantly associated with contraceptive use. Family planning programs that aim at increasing contraceptive uptake should further assess how family planning decision-making processes are achieved in marital unions and how gender roles influence those decisions to increase contraceptive uptake.

## A124. The effect of ten days maximization campaign in improving screening for cervical cancer among women living with HIV in Dar es Salaam

### Jacqueline Mosha^1^, Kalipa Gideon^1^, Goodluck Lyatuu^1^, Wilhellmuss Mauka^1^, Lucy Magesa^1^, Roseline Urio^1^ Dorcas Kushoka^1^ David Sando^1^

#### ^1^Management and Development for Health, Dar es salaam, Tanzania

##### **Correspondence:** Jacqueline Mosha (jmosha@mdh.or.tz)

*BMC Proceedings 2023,* **17(13):** A124

**Introduction**

Women Living with HIV (WLHIV) are susceptible to Cervical Cancer. In Tanzania, the prevalence of Cervical Cancer among WLHIV ranges from 7.3% to 11%. The Ministry of Health emphasized routine screening for early detection and control of pre-malignancies. Unfortunately, in Dar es Salaam, only 9% of WLHIV ever had at least one cervical cancer screening. Thus, the Ten Days Maximization (TDM) Campaign aimed to maximize screening among WLHIV.

**Methodology**

From July to September 2021, MDH-supported health facilities in Dar es Salaam engaged Cervical Cancer Prevention (CECAP) focal and peers who were trained and oriented on screening tools. The 10 days were focused on WLHIV aged 15-49 years, attending CTC clinics that had not been screened for the past 1 year. They were sensitized through health education to participate in screening. Screeners had daily targets monitored by CHMTs, and performance was reported using a specific tool shared via WhatsApp group.

**Results**

A total of 89 CECAP focal paired with respective peers were involved in the TDM campaign in 89 MDH-supported health facilities in Dar es Salaam, each facility had on average 2 screeners, each with a target of 15 clients to screen per day. An average of 40 WLHIV attended CTC during the campaign per day, and 100 % were sensitized, of which 95% of eligible were screened for Cervical Cancer. Each screener could screen between 10 and 20 clients daily reaching a target of 60-70%. Hence, Screening increased fourfold during the TDM campaign (19,378) as compared to routine screening (4,961).

**Implication**

The engagement of WLHIV peers created awareness of the importance of cervical cancer screening. Daily monitoring using a tailored tool in collaboration with the facility authority is the best practice to be adopted. Ten Day Maximization Campaign had increased rates of Cervical Cancer screening than routine screening.

## A125. Facility based single-visit approach: reaching more women for cervical cancer screening and treatment of precancerous lesions

### Amos Nsheha^1^, Shabani Ndama^1^, Henry Musila^1^, Sally Talike Chalamila^1^, Jimson Mgaya^1^, Merensiana Mvungi^1^, Nassoro Yahaya^1^, David Maganga^1^

#### ^1^ HJF Medical Research International, Mbeya, Tanzania

##### **Correspondence:** Jimson Mgaya (jmgaya@wrp.or.tz)

*BMC Proceedings 2023,* **17(13):** A125

**Introduction**

Cervical cancer is the leading cause of cancer-related morbidity and mortality in Tanzanian women. By 2018, it was estimated that 9,772 women were being newly diagnosed with cervical cancer and 6,695 women died of cervical cancer each year. Women living with HIV have 6 times higher risk of developing persistent precancerous lesions and progressing to higher stages that have serious outcomes. The number of women living with HIV that received cervical cancer screening services in 2018 was 3,553 and precancerous lesions treatment rates ranged from 58% to 80% between March and September.

**Methodology**

Henry Jackson Foundation Medical Research International-Tanzania (HJFMRI-T) implemented facility-based single-visit-approach in Mbeya, Songwe, Rukwa and Katavi regions. Prior to that, community campaigns were being implemented. Facility-based single visit approach entails screening and treating of precancerous lesions during a single visit of woman at the facility. To enable this, HJFMRI-T purchased and distributed cryotherapy units and their associated carbon dioxide cylinders to 125 facilities and ensured that all the necessary supplies were available from October 2020 to September 2021. Additionally, HJFMRI-T improved demand creation at the facility and community levels by sharing cervical cancer screening ‘gap’ versus the assigned targets with the health care providers.

**Results**

By October 2021, HJFMRI-T has been able to surpass its cervical cancer screening target of 41,305 by screening 47,731 (116%). The program also was able to treat 884 (94%) women living with HIV out of 938 that were found to have precancerous lesions, a 14% increase from previous performance.

**Conclusion**

Facility-based single-visit-approach is an important strategy to target women living with HIV for cervical cancer screening. This strategy also avoids extra cost for implementing campaigns in the community that usually capture HIV negative women.

## A126. A case study of digital health bridging health facilities and community health systems; the D-tree model

### Grace Mapunda^1^, Kenneth Ogendo^1^, Neema Kafwimi^1^

#### ^1^D-tree International, Dar es salaam, Tanzania

##### **Correspondence:** Grace Mapunda (gmapunda@d-tree.org)

*BMC Proceedings 2023,* **17(13):** A126

**Introduction**

Fragmented and redundant digital healthcare innovations are a challenge globally resulting in digital health solutions failing to reach national scale, but when digital solutions are designed and implemented correctly it has the potential to improve quality, coordination and accessibility of care. D-tree has several case studies in Tanzania demonstrating digital health solutions that reimagine the health systems — leading to better health for Tanzanian families and communities.

**Methodology**

Since 2004, we have designed and implemented innovative digital solutions together with partners and the government of Tanzania by applying human-centred design approach. We recognise government engagement as critical for long term sustainability and impact of our programs, and therefore align with the government from the beginning of programs and ultimately support in scaling and institutionalising the system. In Tanzania, this approach has been employed in our current flagship projects: JamiiniAfya in Zanzibar, and Afya-Tek in Tanzania mainland.

**Results**

Through digital technology we have linked communities and health facilities with 3,149 digitally enabled community and facility health workers, reaching over 1.5 million families. The in-built algorithms and decision support features have improved the quality of healthcare services with a significant increase in the number of follow-up visits, and referrals for needed care. In the Afya-Tek program, over 24.5k referrals have been issued since November 2020, with 85% referral closure rates. Additionally, our flagship program, JamiiniAfya is now rolled out at a national scale with Zanzibar's Ministry of Health endorsement.

**Conclusion**

Linkages between communities and health facilities are crucial for achieving UHC. Digital health can bridge these gaps effectively as demonstrated by the projects and needs to be scaled. However, the existence of fragmented systems is challenging. Unifying the digital tools and strengthening stakeholders collaboration is critical.

## A127. Prevalence and factors associated with acute kidney injury in patients undergoing percutaneous coronary intervention at a tertiary healthcare facility in Tanzania

### Faisal Hooda^1^, Nadeem Kassam^1^, Samina Somji ^1^, Mariam Noorani^1^, Fatma Bakshi^1^, Robert Mvungi^1^

#### ^1^Aga Khan Hospital in Dar es salaam, Dar es salaam, Tanzania

##### **Correspondence:** Faisal Hooda (faisalhooda@gmail.com)

*BMC Proceedings 2023,* **17(13):** A127

**Background**

Acute kidney injury (AKI) is a serious but preventable outcome of percutaneous coronary intervention (PCI): the most plausible cause being radiocontrast-induced nephropathy; others include: pre-existing renal insufficiency from underlying illnesses and also acute renal insult caused by hypoperfusion from cardiac causes. The prevalence, associated risk factors and outcomes of AKI post-PCI are unclear and unexplored in our setting.

**Objectives**

The study sought to determine the prevalence of AKI post-PCI at The Aga Khan Hospital, Dar es Salaam (AKH, D), Tanzania. Secondary objectives included: factors associated, with and outcomes of acute kidney injury (AKI) post-percutaneous coronary intervention (PCI).

**Methods**

A retrospective cross-sectional analytical study was carried out at the AKH, D; enrolling 227 adults who underwent a percutaneous coronary intervention procedure from August 2014 to December 2020. AKI was defined in 2 streams: according to the Acute Kidney Injury Network (AKIN) and by the Contrast Induced (CI) AKI by the Kidney Disease: Improving Global Outcomes (KDIGO) criteria. Our study also looked at factors associated with AKI and outcomes of post-PCI AKI by using bivariable and multivariable logistic regression.

**Results**

A total of 22 (9.7%) patients sustained AKI. Majority of whom were of African descent, and median age of our cohort was 62 years. When bivariable and multivariable analysis were run, they did not show any statistically significant factors associated with AKI. However, the in-hospital mortality rate was 9% for patients with AKI versus 2% for patients without AKI. Patients with AKI had a relatively longer hospital stay (4 days vs. 2 days respectively), required ICU care and organ support; including haemodialysis.

**Conclusion**

Nearly 1 in 10 patients undergoing PCI are likely to develop AKI. This correlates with worsening morbidity and increased in-hospital mortality. Innovative strategies are required to promptly identify and prevent AKI post-PCI.

## A128. Using digitalization to create transparency in healthcare utilization in Zanzibar to inform Health financial strategy development

### Abdul-latif Haji^1^, Ramadhani Abdul^1^ , Wahida Maabad^1^, Heri Marwa^2^, Peter Risha^2^, Mohamed Habib Al-Mafazy^2^

#### ^1^Ministry of Health, Zanzibar, Tanzania; ^2^PharmAccess Foundation, Dar es salaam, Tanzania

##### **Correspondence:** Abdul-latif Haji (abdulatif75@gmail.com)

*BMC Proceedings 2023,* **17(13):** A128

**Introduction**

Currently, health services in Zanzibar are free for all at the point of care and is financed through taxes and donor support. Inadequate financial resources and low Government funding allocation reduce the ability to implement free care policy. The health sector budget amounted to only 7.7%9 (Zanzibar Health budget brief, 2020) of the total budget, lower than the Abuja Declaration’s target (15%). This abstract highlight the contribution of the project toward the review of the health financing strategy for better healthcare.

**Methodology**

Since September 2020, The Ministry of Health (MoHZ) using a digital tool, (Open IMIS®) started to enrol all citizens and issued a MoHZ-approved “KadiyaMatibabu” that has a unique number. At each household, social economic information was collected. All healthcare facilities in a district (8 districts) were connected to the Open IMIS®. Upon seeking care from a connected facility, the information on service use is captured and uploaded into the Open IMIS®. MOHZ carries out periodic data analysis to inform on equity, volume, and cost of healthcare to inform on opportunities for new sources of financing.

**Results**

About 680,000 individuals from 144,290 households were enrolled between September 2020 to June 2022. About 117,000 patient visits were captured in the same period. A review of the utilization data has revealed that the under 5’s and those above 60 years of age constitute about 50% of all facility visits. About 6.8% of free care users were insured and contributed to 5% of the total cost of medicines. Non-residents, 2.5% of all enrollments, consumed more than 3.5 % of all services and more than 3.2% of the total cost of medicines. The information has supported advocacy for new sources of financing.

**Implication**

The findings have provided evidence and supported advocacy for new sources of financing as part of the review of the health financing strategy.

## A129. The role of demand side financing in improving access of reproductive, maternal and child health services at Manyara region

### Theodora Kiwale^1^, Lemali Mbise^1^,Liberatha Shija^1^, Anunsiatha Mream^1^,Emma Mushi^2^, Johnson Yokoyana^1^, Jonia Bwakea^1^

#### ^1^PharmAccess Foundation, Dar es salaam, Tanzania; ^2^Manyara Regional Health Management Team, Manyara, Tanzania

##### **Correspondence:** Theodora Kiwale (t.kiwale@pharmaccess.or.tz)

*BMC Proceedings 2023,* **17(13):** A129

**Introduction**

Over a period of 3 years MomCare project intended to demonstrate how integration of digital and mobile technology into proven clinical quality improvements and demand side financing interventions can transform maternal and newborn outcomes. The project aimed to overcome the financial and quality of care barriers faced by low-income pregnant women and drive uptake of services.

**Methodology**

The MomCare project goals were achieved through digitalizing and scaling up SafeCare quality improvement methodology to improve the quality of care as well as provide access to prepaid, predefined care bundles through a ring-fenced mobile wallet. The payment structure, enabled funding for maternal and neonatal care services uncovered by National Improved Health Insurance Fund (NiCHF). Pay-for-quality model was also used for providers and patients as a way to keep participants motivated in quality attainment and sustaining.8 facilities getting all 3 aforementioned strategies i.e. SafeCare quality improvement support, the whole predefined care bundle as well as top up for quality and client incentives were termed full package whilst the 32 receiving only the first two were termed light package. For data transparency, both light and full package facilities had access to data dashboards which provided real time reports of the provided services.

**Results**

Number of clients starting ANC in the 32 light package facilities increased by 14% from 15,082 per year in 2019 to 17,162 per year by the end of 2021. Proportion of pregnant women attaining at least 4 ANC visits prior delivery increased from 67 % to 100% meanwhile facility delivery raised from 78% in 2019 to 98% by the end of June 2022. Attendance of PNC at 3-7 days improved from of 44% of all deliveries in 2019 to 54% whilst 69% of mothers who delivered are completing the recommended PNC visits.

**Implication**

With the above results it has been shown that the steady availability of improved services enabled by the predefined MomCare bundles and the demand side financing led to increase in number and proportion of mothers getting services at the 32 light package facilities. This shows that improved quality of service on itself can be incentive for mothers to access services.

## A130. Family planning method choices among people with disability: experience from implementing scaling up family planning programme in Tanzania mainland and Zanzibar

### Moke Magoma^1^, Anna Temba^1^, Kathryn O’Connell^1^, Fredrick Msigallah^3^, Suse Matamwa^2^, Deus Ngerangera^1^, Lilian Lukumai^1^, Danielle Garfinkel^1^

#### ^1^EngenderHealth, Dar es salaam, Tanzania; ^2^FCDO Tanzania, Dar es salaam, Tanzania; ^3^CCBRT, Dar es Salaam, Tanzania

##### **Correspondence:** Moke Magoma (MMagoma@engenderhealth.org)

*BMC Proceedings 2023,* **17(13):** A130

**Introduction**

The unique needs of PWDs influence their choices and eligibility for some family planning (FP) methods. However, available national HMIS tools don’t capture information on PWDs and targeted efforts are required to address this gap. The United Nations Convention on the Rights of Persons with Disabilities and the Tanzanian Persons with Disabilities Act recognizes the rights of PWDs need for, and use of, high-quality sexual and reproductive health services.

**Methodology**

A descriptive analysis of Scaling up Family Planning (SuFP) programme outreach service data for February 2020-June 2022. SuFP is a five-year integrated and inclusive FP programme implemented in 600 health facilities across 13 regions in Tanzania. PWDs were identified using a modified Washington Group Criteria. Clients reporting having a lot of difficulty or unable to do any activity were classified as PWD in the respective disability category. People with albinism and severe skin conditions were also categorized as PWDs.

**Results**

1,007,782 outreach clients were reached between February 2020-June 2022, of whom 1.0% (N=10,537) were PWDs. Physical, hearing impairments and intellectual accounted for most disabilities 30%, 19% and 16% respectively. Approximately 9% were adolescents and 98.5% were females. Tanga, Kilimanjaro and Dar es Salaam accounted for 20%, 16% and 15% of all PWDs reached. Approximately 61.8% had never used a FP method before. Implants (77%) were most preferred followed by injectable (9%), IUCD (7%,) Pills (4%,) Condom (2%) and BTL (1%) vs implants (57.4%), injectable (12.9%), IUCD (9.9%,) Pills (9.8%,) Condom (8.1%), BTL (1.6%) and EC 0.3% in the general population.

**Implication**

Despite the National HMIS inability to capture PWD health service data, results from the SUFP program indicate that capturing and monitoring PWD SRH service data is feasible and crucial to ensuring equitable health service utilization. Method choices among PWDs may differ from overall preference of the general.

## A131. Continuous monitoring of FP and RH commodities supply chain system in Tanzania Mainland and Zanzibar: experience from SuFP Programme

### Eric Shoo^1^, Moke Magoma^1^, Lilian Lukumai^1^, Kathryn O’Connell^1^, Danielle Garfinkel^1^, Ramadhan Mlange^1^, Deus Ngerengera^1^

#### ^1^Engender Health Tanzania, Dar es salaam, Tanzania

##### **Correspondence:** Eric Shoo (Eshoo@engenderhealth.org)

*BMC Proceedings 2023,* **17(13):** A131

**Introduction**

The recent national LMIS improvement at all levels, including changing MSD E9 to E10 with customer portal aimed at strengthening commodity security tracking and ordering. Specifically, eLMIS was redesigned from quarterly to bimonthly reporting and ordering in Tanzania Mainland and from quarterly to monthly ordering in Zanzibar. Nevertheless, FP/RH commodities stock-outs still occur. Services are variously affected when essential commodities are lacking.

**Methodology**

Conducted monthly and quarterly MSD E10 and eLMIS monitoring for stock availability at national, zonal and health facility levels complemented by health facility visits in 13 SuFP Programme-supported regions in Tanzania. Approximately 100 health facilities from SuFP Programme regions are jointly visited quarterly during which commodity stock-level gaps are identified and addressed through on-job mentorship and implementation of joint improvement plans. Zonal commodities security meetings with key actors in SRH commodity security are also used to address challenges.

**Results**

Stock-out levels of FP commodities at MSD were rare between July 2020- June 2022 except for a small shortage of Implanon NXT and Depo Provera injection. The stock out reported through eLMIS reduced from 18% (April – June 2020) to 7.3% (April – June 2022) while the stock out reported through health facility visits assessment witnessed a reduction from 50% (July – September 2020) to 5% (April – June 2022). Understocked health facilities declined from 25% to 17% and adequately stocked increased from 11% to 23%. Nevertheless, overstocked and unknown stock status health facilities increased from 13% to 19% and 0% to 37% respectively.

**Implication**

Continuous monitoring of the MSD E10 and eLMIS enabled timely recognition of commodity stock levels which informed timely and appropriate remedies. Joint planning and implementation remain key to these achievements.

## A132. Factors affecting linkage to HIV care and treatment services among orphans and vulnerable children newly diagnosed with HIV in Tanzania

### Amon Exavery^1^

#### ^1^Pact, Dar es Salaam, Tanzania

##### **Correspondence:** Amon Exavery (aexavery@pactworld.org)

*BMC Proceedings 2023,* **17(13):** A132

**Introduction**

In 2019, only 78% of Tanzanian children living with HIV were on antiretroviral therapy (ART), suggesting issues with linkage to HIV care and treatment (C&T) services. This study examined factors affecting linkage to C&T services among orphans and vulnerable children (OVC) newly diagnosed with HIV.

**Methodology**

OVC enrolled in the community-based USAID-funded Kizazi Kipya project in 24 regions of Tanzania during 2017–2020 who were HIV negative or unknown status at enrollment were further assessed for HIV risks, after which they were referred for HIV testing if at risk. The referrals were tracked by the project, and referral feedback documented. OVC newly tested HIV positive were linked to HIV care and treatment centers (CTCs) to start HIV C&T services. Linkage to CTC was considered successful if the OVC was assigned a CTC-issued identification number, otherwise it was unsuccessful. Factors associated with linkage to CTC were identified using multivariable logistic regression.

**Results**

Of the 6,174 OVC (53.4% female) aged 0–17 years who newly tested HIV positive, 96.4% were successfully linked to a CTC for C&T services as of 30 March 2021. In the multivariable analysis, factors with significant positive association with successful linkage to CTC were living in an urban area (OR=1.43, 95% CI 1.04–1.94) and having health insurance (OR=1.58, 95% CI 1.02–2.44). Caregiver age was a negative factor (OR=0.99, 95% CI 0.979–0.999).

**Conclusion**

The project support to OVC newly diagnosed with HIV resulted in a high rate (96.4%) of linkage to CTC in Tanzania. Although the unreached were few, there is a need to devise additional strategies so that no one is left behind, by addressing critical barriers including rural residences where problems with transport and distance to facilities are substantial, lack of health insurance as well as prioritizing OVC living with elderly caregivers.

## A133. Red blood cell parameters and associated factors among infants attending sangabuye and makongoro health centres, Mwanza, Tanzania

### Rotgen A. Elichilia ^1^, Caroline A Minja^2^ Delfina R Msanga^3^ Mariam M Mirambo^4^

#### ^1^Intern medical laboratory scientist at Muhimbili National Hospital, Dar es salaam, Tanzania; ^2^Department of Biochemistry and Molecular biology, Catholic University of Health and Allied Sciences, Mwanza, Tanzania; ^3^Department of Pediatrics, Catholic University of Health and Allied Sciences, Mwanza, Tanzania; ^4^Department of Microbiology and Immunology, Catholic University of Health and Allied Sciences, Mwanza, Tanzania

##### **Correspondence:** Rotgen A. Elichilia (myahudi20@gmail.com)

*BMC Proceedings 2023,* **17(13):** A133

**Background**

Red cell parameters are useful anaemic and inflammatory markers in diagnosis and monitoring of diseases. Result interpretation for these parameters is population and ethnic specific. However, among infants, establishment of normal values is difficult due to recruitment difficulties, together with the dynamic growth and development patterns. This study aimed to assess RBC parameters and associated factors among infants attending Sangabuye and Makongoro Health Centres in Mwanza Tanzania.

**Methodology**

A cross sectional hospital-based study involving 200 infants attending post-natal clinics was conducted. Using a structured questionnaire, demographic and other study related data was collected and tested in the auto analyzer (Dymind DH-76). Data was analyzed using STATA version 13.

**Results**

Majority of study participates were female 164 (54.7%) with a mean (SD) age of 6 (±3) months. The mean Hb, MCV, and Erythrocyte count was 10.3 (± 1.25) g/dl, 73.1 (± 9.3) mm3 and 4.47 (± 0.87) x 106/ mm3 respectively. By two sample t- test, only infants’ current breast-feeding status had an effect on erythrocyte count (p=0.0009). On univariate analysis age (p=0.001) and body temperature (p=0.001) had effects on erythrocyte count while body temperature alone had effect on both erythrocyte count (p=0.009) and haemoglobin (p=0.026)

**Conclusion**

The Red Cell Parameters values determined from this study are of immerse importance in patient care and hematological dependent clinical trials. This study has addressed the population gap among infants. This study has highlighted a lower erythrocyte count value in our setting compared to standard reference interval. We recommend further locally derived age-specific studies, with large samples size and wide range assessment of disease screening.

## A134. Finding missing people with TB: Traditional healers premise a potential high yield community entry point experience from Geita region, Tanzania

### Waida R. Jabiri^1^ Rose Olotu^1^, Godwin Munuo^1^Frida Ngalesoni^1^, Aisa Muya^1^John Msaki^2^ Paschal Wilbroad^3^Michael Mashala^4^CleophacyProspery^4^

#### ^1^Amref Health Africa in Tanzania, Dar es salaam, Tanzania; ^2^National TB and Leprosy Program, Dar es salaam, Tanzania; ^3^USAID Tanzania, Dar es salaam, Tanzania; ^4^Regional Health Management Team - Geita Region, Tanzania

##### **Correspondence:** Waida R. Jabiri (waida.ramadhani@amref.org)

*BMC Proceedings 2023,* **17(13):** A134

**Background**

It is estimated that 60-80% of the people in African countries, including Tanzania, use traditional medicine (TM) as their primary source of health services. Geita region account for about 2,557 registered traditional healers (TH) who provide traditional health care services in their communities. Recently, the region has realized several TH suffering from TB indicating TB transmission within TH premises. Amref Health Africa in Tanzania in collaboration with partner CBOs is implementing the USAID AfyaShirikishi interventions which are aiming at reducing TB prevalence in the community.

**Methodology**

In collaboration with Local Government Authorities (DTH Coordinators, WEO & VEO) a total of 52 TH premises with high volume clients were visited purposely between July 2021 and March 2022. The engaged group were oriented on TB screening and linked with 48 Community Health Care Workers (CHWs) who conducted TB screening using a standardized TB screening tool, community TB register and referral form. Sputum specimen were collected from presumptive TB individuals and transported to the nearby TB diagnostic health facility.

**Results**

A total of 211 people attended to the traditional healers between July, 2021 to June 2022 were screened for TB and 97 among them were presumptive for TB. All 97 presumptive TB individuals were referred for testing and 27(28%) were confirmed to have TB of which 8(30%) cases were from a family member of one traditional healer. All cases were linked with health facilities and initiated TB treatment.

**Conclusion**

The results call for meaningful engagement of Traditional Healers in finding missing individuals with TB to reach the End TB goal. This calls for a robust TB screening intervention among traditional healers’ premises and their clients.

## 135. The contribution of integrative medicine practice to patient improvement

### Elizabeth H. Lema^1^

#### ^1^Cornwell Company, Dar es salaam, Tanzania

##### **Correspondence:** Elizabeth H. Lema

*BMC Proceedings 2023,* **17(13):** A135

**Background**

Diabetes mellitus is a major risk factor for Atherosclerotic Cardiovascular Disease (ASCVD) and premature mortality. Tanzania is experiencing a higher burden of non-Communicable disease (NCDs) with ASCVD being the most prevalent especially among adults aged 40 years and above. Modifiable risks are variable amongst different population and central to the development of ASCVD. This study aimed to identify magnitude of ASCVD among a diabetic cohort and its associated risk enhancing factors amongst a cohort of patients in Tanzania.

**Methods**

An observational prospective study conducted at the Aga Khan hospital, Mwanza. Individuals 10 – year risk was calculated based on the ASCVD risk calculator developed by American Health Association (AHA) and American college of cardiology (ACC) in 2013. Multivariable analysis was applied to determine risk enhancing factors for individuals who had a high 10-year risk of ASCVD.

**Results**

The study recruited a total of 99 participants over a period of 3 months. The median age of the study population was 60 years [IQR: 52-68] years, most of whom were of African origin (n=89, 89.9%), having a BMI of >30kg/m^2^ (n=57, 57.6%)and suffering with concomitant Hypertension (n=64, 64.6%). Majority of our cohort had a high 10 -year risk of suffering ASCVD (n=59, 59.6%)The studyidentified duration of Diabetes Mellitus (>10 years) (OR 8.15, 95% CI 2.47–31.20) and increased creatinine (OR 1.03, 95% CI 1.01–1.05) to be risk enhancing factors associated with high 10 year risk of ASCVD amongst our diabetic cohort.

**Conclusion**

This study demonstrates the predictive burden of ASCVD amongst diabetics and validates the great need of comprehensive patient centered approach for primary prevention of ASCVD which includes optimal glycemic and blood pressure control and the need of statin as cholesterol lowering agent.

## A136. Integrating gender-based violence screening services among client attending outreach services: lessons from scaling up family planning programme in Tanzania

### Moke Magoma^1^, Deus Ngerengera^1^, Lilian Lukumai^1^, Suse Matamwa^1^, Katanta Simwanza^1^, Ana Aguilera^1^, Kathryn O’Connell^1^, Renu Golwalkar^1^, Danielle Garfinkel^1^,

#### ^1^EngenderHealth Tanzania, Dar es salaam, Tanzania

##### **Correspondence:** Moke Magoma (MMagoma@engenderhealth.org)

*BMC Proceedings 2023,* **17(13):** A136

**Introduction**

Gender-based violence (GBV) is widespread in Tanzania—approximately 40% of women aged 15 to 49 experience physical violence and 17% experience sexual violence during their lifetimes (TDHS 2016). Overall, 40% of women aged 15-49 ever experience emotional, physical and/or sexual violence. Women who experienced GBV are less likely to access and utilize health care services and to make their own decisions regarding their Sexual and Reproductive Health (SRH).

**Methodology**

A descriptive analysis of Scaling up Family Planning (SuFP) programme implementation outreach service data for February 2020-June 2022. SuFP) programme-a five-year integrated and inclusive FP programme implemented in 600 health facilities across 13 regions in mainland Tanzania and Zanzibar. All clients reached during outreach service were screened for GBV using the national GBV screening tool.

**Results**

A total of 1,007,782 outreach clients were reached between February 2020-June 2022, of whom 48% (N=485,040) were screened for GBV and 5% (N=23,070) were categorized as GBV survivors (2.2% were male and 97.8% female), (18.5% adolescent (10-19 years) and 81.5% (20+ years)) with 6.7% (N=32,514) incidences. Of all the GBV incidents recorded, emotional, physical, and sexual violence accounted for 62%, 28%, and 10% respectively. A total of 3,998 clients were referred for post care services and further management.

**Implication**

Adaptation of GBV screening tool used in the outreach services provided the opportunity for strengthening service providers’ capacity to identify and link GBV /VAC survivors to appropriate care and support. Ensuring GBV/VAC screening at all service delivery points is imperative for timely identification and linking survivor to services.

## A137. Tanzania immunization registry (TIMR) and DHIS2 interoperability for immunization information use

### Ismail Koleleni^1^, Tuzo Engelbert^1^. Wilfred Senyoni^1^

#### ^1^HISP Tanzania, Mazinde Street, Dar es salaam, Tanzania

##### **Correspondence:** Mr. Ismail Koleleni (ismailkoleleni@hisptanzania.org)

*BMC Proceedings 2023,* **17(13):** A137

**Introduction**

Prior to the Electronic Immunization Registry (TImR) in 2016, immunization information was digitalized down to the district level, and paper-based at the health facility level. Monthly paper reports were submitted to District managers, then into DHIS2, and Vaccine Information Management System (VIMS). The system generated routine aggregate registered children receiving vaccinations, vaccines administered and identified children who are off schedule reports for sharing with data managers at district and above levels. The parallel systems (i.e., TImR, VIMSandDHIS2) led to data duplication and discrepancy among systems, and little to no collaboration among departments, hence inconsistent immunization information reports.

**Methodology**

The MoH with its partners, HISP Tanzania, JSI and PATH embarked on a mission of integrating Immunization Systems and improving the accessibility of quality immunization data within the national DHIS2 platform. HISP Tanzania ensured systems interoperability between EIS and DHIS2, starting with two paperless regions (i.e., Mwanza and Kilimanjaro region), and capacitated them to report using TImR and remove manual reporting through HMIS registers. TImR was configured through a web-based portal to aggregate and send immunization data into DHIS2, and share vaccine and logistics information to VIMS for management purposes. VIMS, on the other hand, sends stock data to TImR for management and distribution of immunization stock at the health facility level.

**Results**

Interoperability addressed systems fragmentation challenges by providing a common shared source of immunization information for data managers at national and sub-national levels for decision making. HMIS and EPI departments can now use the shared DHIS2 platform for analyzing and disseminating immunization information for better planning and monitoring performance. Stakeholders can share information and strengthen the collaboration among participating actors. The interoperability has reduced the burden of redundancy data collection for healthcare workers, as well as management costs in ‘paperless' regions.

**Implications**

The integration between Electronic Immunization Systems (EIS) and DHIS2 provided a benchmark for strengthening immunization data management and information use at national and sub-national levels. HISP Tanzania continues to plan and work with MoH and its stakeholders to roll out the integration beyond the two pilot regions. Also, capacitating users towards using digital tools for reporting to sustain the integration of EIS and DHIS2 as well as the EIS.

## A138. Prevalence of mental health problems and associated factors among secondary school adolescents and young people in Kilimanjaro region

### Jackline T. Shirima^1^, James Samwel Ngocho^1^, Rehema Ahmed Mavura^1^, Innocent B Mboya^1^, Lisbeth Mhando^1^

#### ^1^Kilimanjaro Christian Medical Center, Moshi, Tanzania

##### **Correspondence:** Jackline T. Shirima (Jacklinethadei@gmail.com)

*BMC Proceedings 2023,* **17(13):** A138

**Introduction**

Half of mental health problems in adults begin before 14 years. When not treated, adolescents and young people continue to be at high risk for abuse, suicide, substance use, and school failure which have long-term consequences that negatively impact physical, mental health, and economic productivity. The study aimed to determine the prevalence of mental health problems and associated factors among secondary school adolescents and young people in the Kilimanjaro region.

**Methodology**

The study utilized secondary data from two repeated cross-sectional Regional School-Based Student Health Surveys conducted in 2019 (survey 1) and 2022 (survey 2) among students (20-24 years) from four districts in Kilimanjaro region. SPSS 20 was used for data analysis. Chi-square test was used to compare mental health problem proportions by survey year and other participant characteristics. Multivariable logistic regression analysis to estimate odds ratios and 95% CI to determine factors associated with mental health problems at 5% threshold level.

**Results**

Students with median age 15, females (53.9%), survey 1 (65%) participated in the study. Among 4955 secondary school students, the overall prevalence of mental health problems was 29.2% (survey 1; 27.4% and survey 2; 32.6%). The most frequent mental health problem was ever being worried (11.2%). Higher odds of mental health problems were among those aged 20-24 years (aOR=3.37, 95% CI 1.44-7.90), females (aOR=1.39, 95% CI 1.12-1.74), survey year (aOR=1.37, 95% CI 1.01-1.86), current substance users (aOR=2.09, 95% CI 1.65-2.64), ever had sex (aOR=1.56, 95% CI 1.17-2.07), ever bullied (aOR=2.54, 95% CI 1.87-3.46), ever missed class (aOR=1.28, 95% CI 1.02-1.61).

**Conclusion**

The study utilized secondary data from two repeated cross-sectional Regional School-Based Student Health Surveys conducted in 2019 (survey 1) and 2022 (survey 2) among students (20-24 years) from four districts in Kilimanjaro region. SPSS 20 was used for data analysis. Chi-square test was used to compare mental health problem proportions by survey year and other participant characteristics. Multivariable logistic regression analysis to estimate odds ratios and 95% CI to determine factors associated with mental health problems at 5% threshold level.

## A139. Implementation of afya ya kijani: a physiotherapist-supported healthy lifestyle program for individuals with diabetes

### Kelvin Haukila^1^, Janet Bettger^2^, Colleen Burke^3^, Mathew Shayo^1^, Pendo Shayo^1^, Katherine Norman^3^

#### ^1^Kilimanjaro Christian Medical Center, Moshi, Tanzania; ^2^Temple University, Philadelphia, USA; ^3^ DUKE University, Durham, USA

##### **Correspondence:** Kelvin Haukila (kelvinhaukila@gmail.com)

*BMC Proceedings 2023,* **17(13):** A139

**Introduction**

Diabetes is a leading risk factor for death and disability worldwide. In Tanzania, the rapidly increasing prevalence is accelerating the burden of chronic non-communicable diseases (NCDs). Disease self-management programs are effective for improving healthy lifestyles of people with diabetes but clinician-supported interventions can be challenging to implement in low-resourced settings and for patients less likely to attend the clinic.

**Methodology**

Prospective pragmatic intervention study of a disease self-management program to promote health lifestyles among adults with diabetes. Patients attending a hospital-based diabetes clinic were referred for follow-up by a physiotherapist for evaluation, education, counseling and goal setting for a healthier lifestyle. Data were analysed descriptively.

**Results**

Sixty-seven people were recruited; to-date 21 completed 4 months in the program. Patients' mean age was 61 years, 60% females 40% males, 50.7% were still working (not retired), and all were from Kilimanjaro region. Each person received an instruction booklet with a health tracking log for blood pressure and blood sugar levels, medication, activity, diet, tobacco and alcohol use. Patients agreed to specific goals in the first meeting: becoming physically active, eating healthy and reduced alcohol intake were most common. The physiotherapist sent two text messages and provided telephone counseling each week. Patients reported to complete daily goals 79.01% on average in the first month Challenges to longer term maintenance include difficulty in incorporating this program in diabetes clinics as most of these clinics are very busy due to the unbalanced ratio of patients to clinicians.

**Implication**

This program was feasible and showed a positive direction towards comprehensive management of not only diabetes but also other chronic NCDs sharing similar risk factors.

## A140. Achieving the elimination threshold for trachomatous trichiasis in Sumbawanga district, Tanzania

### Alex Msumanje^1^, Lali Chania^1^, Ntuli Mwaingwisya^1^, Katina Sommers^1^, Juma Ngakola^1^, George Kabona^2^, William Ngella^1^

#### ^1^IMA World Health- Tanzania, Dar es salaam, Tanzania; ^2^Program Manager for NTD in Tanzania, Dar es salaam, Tanzania

##### **Correspondence:** Alex Msumanje (AMsumanje@imaworldhealth.org)

*BMC Proceedings 2023,* **17(13):** A140

**Background**

Trachomatous trichiasis (TT) is the sight-threatening result of conjunctiva scarring from repeated trachoma infections. A simple, outpatient eyelid surgery can correct the in-turned eyelashes. In 2014, IMA was given targets for each district by the Ministry of Health (MoH) and was assigned to implement trichiasis case management in Mtwara, Pwani, Rukwa, Kagera, Simiyu, and Mwanza. In 2020, the MoH conducted trachoma impact surveys, targeting districts that had achieved targets. The findings revealed persistence of unmanaged TT in previously intervened districts.

**Results**

The survey findings prompted the MoH to revise guidelines for TT elimination as follows. 1.Train one case finder per sub-village in every district 2. Achieve ≥90% households visited for case finding by trained case finder in every district 3. Achieve ≥90% of TT suspects by case finders screened by TT screener in every district 4. Manage ≥95% of confirmed cases by TT screeners in every district 5. Present to the government authorities documentation of intervention activity completed in every district. In 2022, IMA worked with District Eye Coordinator, village leaders and case finders in Sumbawanga and achieved, 98.2% of households were visited by case finders for case finding, 93.8% of eligible household member screened for TT by case finders, 799 TT suspects were listed by case finders, 98.2% of suspects were screened for TT by screeners, 412 cases were confirmed TT by screeners, 100% of confirmed TT cases were managed by TT surgeon.

**Conclusion**

IMA, and the DEC for Sumbawanga, have established one static surgical site for a TT surgeon to manage any remaining cases in the area. Full execution of the intervention by government eye care workers, and close monitoring of the interventions by the MoH, has contributed to achieving targets for Sumbawanga.

## A141. Knowledge, attitude and practices on HPV vaccination among female undergraduate students at MUHAS

### Yusta F. Kimario^1^

#### ^1^Muhimbili university of health and allied science (MUHAS), Dar es salaam, Tanzania

##### **Correspondence:** Yusta F. Kimario (yustakimario94@gmail.com)

*BMC Proceedings 2023,* **17(13):** A141

**Background**

Human papilloma virus (HPV) types 16 and 18 have been proven as primary causes of cervical cancer which is the fourth most common cancer in women worldwide, hence the introduction of HPV vaccines. Knowledge and practices on HPV vaccination among female students is vital as these may strongly determine intention to recommend vaccination to others and themselves in the future.

Aim: To assess the knowledge, attitude and practices regarding HPV vaccination among female undergraduate students.

**Methodology**

Across-sectional study was conducted using a convenient sampling technique through self-administered questionnaire,359 participants were recruited. Data were analyzed using an SPSS version 23 software.

**Results**

Overall level of knowledge being low as majority 324(90.3%) failed to answer a total of 4 and above knowledgeable questions. 282(78.6%) had negative attitude on HPV vaccination whereby293 (81.6) believed that the HPV vaccine provides more benefit than harm in preventing cervical cancer and 234(65.2) would have vaccinated against HPV if the vaccination was freely available. Only 25(7.0%) have ever been vaccinated with minimal number of doses received being one shot by 22(6.0%) and only one had succeed to receive full dose. Major reason for not being vaccinated being lack of awareness by 93(25.9%). About 257(71.6%) are unaware of the cost. 305(85.0%) would wish to receive vaccination if it offers protection against cervical, anal cancer and genital warts.

**Conclusion**

There is low knowledge level on HPV vaccination among female undergraduate students at MUHAS being worst among non-medical students; this has attributed to majority with negative attitude hence low coverage of HPV vaccination. There is a need for more awareness programs and education about the effective methods to prevent cervical cancer together with vaccination programs. HPV vaccines need to be subsidized to make them affordable for university students.

## A142. Scaling up COVID-19 vaccine uptake in general population: Experience from Kigoma region, Tanzania

### Hans Maro^1^, Redempta Mbatia^1^, Damian Laki^1^, Alexander Christopher^2^, Julius Zelothe^1^, Daniel Magesa^1^, Mushubira Balinda^2^, Frederick Ndossi^1^, Kokuhabwa Mukurasi^2^

#### ^1^Tanzania Health Promotion Support, Dar es salaam, Tanzania; ^2^Centre of Disease Control Tanzania, Dar es salaam, Tanzania

##### **Correspondence:** Hans Maro (hmaro@thps.or.tz)

*BMC Proceedings 2023,* **17(13):** A142

**Introduction**

Tanzania officially launched its Covid-19 vaccination Programme in July, 2021 aiming to reduce risk of hospitalization and mortality especially among those at higher risk. Despite the benefits of COVID-19 vaccine, but still Kigoma region were lagging far behind the target of vaccinating 70 % by December, 2022 of 18yrs and above. By May, 2022 only 86,379 (6.3%) of the eligible population in Kigoma were vaccinated.

**Methodology**

THPS, through CDC funds, started vaccination scale up in February, 2022, aiming to increase COVID-19 vaccine uptake in general population by conducting Strategizing meetings with regional and district health pollical leaders, R/CHMTs, Facility in charges.; with a goal of reaching the target by December, 2022. Also, grassroot political//influential leaders; among strategies were, Every HF must conduct one outreach per day, Vaccinators will be paid by number vaccinated, increase facility vaccination points, Daily performance monitoring at facility level.

**Results**

By 28th July, 2022, 715,381 (52%) were fully vaccinated with 768,354 received at least one dose of Covid-19 vaccine in Kigoma. The average weekly vaccination is approximately 85,000 as of July, 2022.

**Implication**

Pay per performance, involvement of R/CHMTs, political and grassroot leaders; availability of vaccines and outreach services are key to reaching general population with the COVID 19 vaccination.

## A143. Use of experienced patients for improved adherence and retention to care and treatment among PLHIV in Shinyanga, Pwani and Kigoma regions

### Noela Nsanzugwanko^1^, Japhet Daud^1^ Willium Hosea^2^ Aaron Tesha^1^ Benedicta Masanja^3^ Redempta Mbatia^1^ Eva Matiko^4^ Angelo Minja^1^

#### ^1^Tanzania Health Promotion Support, Dar es salaam, Tanzania; ^2^Kigoma region Health Management Team, Kigoma, Tanzania; ^3^Tanzania Promotion Support, Dar es salaam, Tanzania; ^4^CDC Tanzania, Dar es salaam, Tanzania

##### **Correspondence:** Noela Nsanzugwanko (nnsanzugwanko@thps.or.tz)

*BMC Proceedings 2023,* **17(13):** A143

**Background**

Tanzania Health promotion support (THPS) through PEPFAR supported in Pwani, Kigoma and Shinyanga regions had 141,060 clients current on HIV care and treatment by March 2022; with 15,669 (11%) having over 10 years of ARV since HIV diagnosis. Small miners, long distance truck drivers, mobile farmers, charcoal makers, female sex workers and drug users, are at a higher risk of interrupting ART. A total of 1,157 clients had treatment interruption by March 2022.

**Methodology**

For continuity of treatment, THPS adapted evidence-based adherence and retention strategies through meaningful engagement of experienced patients (EP) as peer educators and champions of extended linkage case management, continuous follow up and counselling. The EP support include MMD for stable patients, provider-patient tie, reminder calls, 3 boxes approach, map cue to capture client additional demographic information, and same day tracing for missed appointments. Sub populations specific interventions were designed to ensure they continue with their economic activities without treatment interruptions.

**Results**

Experienced patients are stable and have good outcomes with 12,515 (80%) being on multi months drugs and 15,300 (98%) are virally suppressed. In comparison, 125,926 PLHIV with less than 10 years on ART 78% are on MMD and 90% are virally suppressed by March 2022.

**Conclusion**

Local EP experiences are very important on helping programs design informed and acceptable adherence and retention strategies among PLHIV. Continuous care, adherence, psychosocial support and retention strategies by the EP will help recipients of care remain connected to health services. Adaptation of these strategies countrywide and taking them to scale will result to improved clinical outcomes among PLHIV.

## A144. Feasibility of implementing non-communicable disease (ncd) and hiv integrated services in hiv care and treatment clinics of ilala city council, Dar Es Salaam- Tanzania

### Jacqueline Meshack^1^

#### ^1^Muhimbili University of Health and Allied Sciences, Dar es salaam, Tanzania

##### **Correspondence:** Jacqueline Meshack

*BMC Proceedings 2023,* **17(13):** A144

**Introduction**

A primary concern with non-communicable diseases (NCDs) is the morbidity and mortality that they cause. Integration is an evidence-based strategy, suggested to combat co-morbidities of chronic diseases specifically NCDs and HIV. The suitability of NCDs/HIV integration from implementers (providers, council leaders) in Tanzania is less explored. We explored factors influencing feasibility of integrating NCDs/HIV services in HIV clinics located in the primary health care facilities in urban Dar es Salaam.

**Methodology**

We conducted 20 Key Informant Interviews with 17 providers and 3 council leaders from five HIV clinics high client volume, focusing on those initiated on antiretroviral treatment (ART). A semi-structured interview guided by Consolidated Framework of Implementation Research (CFIR) assessed intervention characteristics, inner settings and individual characteristics influencing the implementation of evidence-based practices into routine care. Data collected were transcribed, translated from Swahili to English, and analyzed using a deductive framework analysis approach.

**Results**

Integrating NCD/HIV services at HIV clinic is feasible as participants were aware of the NCD burden among HIV clients and described the advantages of integration to include reduction in cost, time, and movement in accessing services. Continuous engagement, clinical experience in service provision, documentation of services in client forms, teamwork, task sharing and motivation, were identified to facilitate integration services. There were also HIV clinics infrastructure challenges and insufficient guideline implementation that could impede the provision of integrated NCDs/ HIV service. However, this study has revealed it is possible to implement integrated NCDs/ HIV intervention at the clinics.

**Conclusion**

This study has shown it may be feasible to implement an integrated NCDs/HIV intervention at HIV clinics of PHC level. More advocacy and monitoring of guideline use by ministry of health while collaborating with CHMTs and PHC providers as key stakeholders in NCD/HIV integration endeavors when developing and implementing strategies.

## A145. Outcomes of exposure to SBC platforms in improving nutritional practices among women of reproductive age and children under

### Victoria Marijani^1^, Alex R. Rutto^1^, Lydia Clemmons^1^, Joyceline E. Kaganda^1^, Carolyn O’Donnell,^1^Ben Stephens1, JoanitaAigi Muruve^1^,

#### ^1^Save the Children International, Dar es salaam, Tanzania

##### **Correspondence:** Victoria Marijani (Victoria.Marijani@savethechildren.org)

*BMC Proceedings 2023,* **17(13):** A145

**Introduction**

The prevalence of childhood stunting in Tanzania is 31%, much higher than the global estimates at 22.2%, making it a public health concern. Improved nutrition practices during the first 1000 days of a child’s development can prevent stunting. Studies have shown that exposure to multiple social behavior change (SBC) platforms contributes to improved nutrition practices. This study aimed at assessing behavior uptake among project beneficiaries enrolled in SBC platforms.

**Methodology**

Focusing on the first 1000 days, Lishe Endelevu utilized multiple SBC platforms to promote maternal, infant, and young children nutrition (MYCN) and WASH behaviors. These included nutrition counseling at ANC/PNC clinics, home visits and nutrition education by community health workers, and mHealth messaging. A midline survey was conducted collecting quantitative and qualitative data to assess behavior uptake among project beneficiaries enrolled in SBC platforms.

**Results**

Results showed that exposure to nutrition knowledge through the SBC platforms led to improved IYCF practices: 31.2% of children 6-23 months receiving a minimum acceptable diet (baseline=11.9%), 18.5% of women of reproductive age consuming a diet of minimum diversity (baseline=11.7%), 33 percent of households had improved and non-shared toilet facilities with hand washing stations with running water and soap (baseline=13%). 9.5% of those who received counselling from CHWs were more likely to wash their hands during the 6 critical times compared to those who did not receive CHW counselling at 5.7%, therefore there is a strong association.

**Conclusion**

Generally, results revealed that there is a correlation in terms of participants’ exposure to various SBCC platforms with their improved outcomes in nutrition and WASH behaviors. To achieve expected results, the SBC approaches should be very specific and messages should be simple and clear in each platform.

## A146. Transforming father’s roles in nutrition by understanding men’s social expectations

### Victoria Marijani^1^, Lydia Clemmons^1^, Sylvia Rutagumirwa^1^, Joyceline Kaganda^1^

#### ^1^Save the Children International, Dar es salaam, Tanzania

##### **Correspondence:** Victoria Marijani (Victoria.Marijani@savethechildren.org)

*BMC Proceedings 2023,* **17(13):** A146

**Introduction**

A challenge in nutrition is how to effectively engage fathers, who often see nutrition services and home practices as women's spaces and roles and leave them to women. An unintended consequence of engaging fathers in health and nutrition activities is that men begin to advice or even oversee women in areas that are traditionally under women's control, eroding their autonomy.

**Methodology**

USAID/Lishe Endelevu project implemented an intervention design that aimed to positively engage fathers in a male friendly way by understanding family systems and cultural norms in line with gender roles. In designing the SBC communication plan and activities to engage family members, a gender analysis was conducted, through interviews and focus group discussions with men and women. Group discussions used photo pile sorts to understand social perceptions of "ideal" gender roles for men and women.

**Results**

Findings revealed wide variation between perceptions of "normal" men and "ideal" men, and outlined actions embedded in gender roles that fathers can take, grouped as protect, provide, and decide (P-P-D). For example, fathers can protect family health, provide nutritious food for the family, and decide together, including financial planning for nutrition and health care. Creating an enabling environment at the household level makes it easier for men to adopt improved.

**Implications**

The social change strategy has shown that it can reduce the contradictions between the "ideal" and "normal" man. It transforms norms by empowering women and liberating men from the heavy social expectations and peer pressure that oblige them to demonstrate their power and dominance over women.

## A147. Advancing respectful maternity care through birth companionship practice in public facilities in Tanzania, updates from Kigoma and Katavi

### Sunday Dominico^1^, Alex Mputa^1^, Banzi Msumi^1^, Agnes Mbanza^1^

#### ^1^Thamini Uhai, Dar es salaam, Tanzania

##### **Correspondence:** Sunday Dominico (sdominico@thaminiuhai.or.tz)

*BMC Proceedings 2023,* **17(13):** A147

**Introduction**

Providing respectful care to women during childbirth is a key component of good quality maternal health care and a human rights matter. Having a companion of choice throughout childbirth is widely accepted to be a critical component of respectful maternity care. Thamini Uhai, who systematically introduced birth companionship practice during childbirth in public facilities in Tanzania, have developed a cost-effective implementation model to be used in low resources settings.

**Methodology**

The third phase of Birth companionship project implementation (May 2021 – April 2023) involves 23 implementing facilities in Kigoma and Katavi regions, 10 facilities increase from previous phase. Components of implementation model are: 1) labor rooms infrastructure upgrade to ensure visual and audio privacy 3) orientation of health care providers 4) community sensitization 5) recruitment and training of community health workers (CHWs) 6) Conducting community score card to ensure accountability 7) distribution of comfort measure tools 8) quarterly supportive supervision.

**Results**

Birth companionship intervention in Kigoma and Katavi now covers a total of 12 councils and 23 facilities, in catchment population of about 2million people. There is growing interest at the national level, with the Ministry of Health advocating for learning visits to Katavi to understand the implementation model. Two learning visits was done by government entity and partner and Thamini Uhai invited to share experience at the first ever national RMNCAH scientific conference in November 2021.

**Implication**

Birth companionship model has been scaled up to 10 new implementing facilities and well sustained in 13 existing facilities with minimal project support. Birth companionship facilities have been used as learning sites to others including government entities and implementing partners for possible replication. Further scale-up is feasible to ensure countrywide practice.

## A148. The use of partograph in monitoring labour among obstetric care providers in ulanga district council

### Benatus Sambili ^1^

#### ^1^SolidarMed, Ifakara, Tanzania

##### **Correspondence:** Benatus Sambili (b.sambili@solidarmed.ch)

*BMC Proceedings 2023,* **17(13):** A148

**Background**

Prolonged labour is a leading cause of death among mothers and newborns in developing countries, which can be prevented by accessing skilled delivery services such as plotting partograph during the progress of labour. Unfortunately, this inexpensive and effective tool is not extensively applied as it should be in many African countries, Tanzania inclusive. Understanding the current level of partograph use and factors influencing its use among obstetric care providers (OCPs) will be crucial to improving the quality of obstetric care services and reducing maternal and perinatal wastages.

Objective: To determine the use of partograph in monitoring labour among obstetric care providers in Ulanga District Council.

**Methodology**

This cross-sectional study used a mixed-methods approach. A quantitative study included all the partographs for births five months before the study. A checklist retrospectively determined the documentation and the level of partograph parameters used to monitor labour progress at five health facilities. For the qualitative study, ten in-depth interviews were conducted. Participants were drawn purposively from the labour and delivery room for homogenous sample. They were interviewed using an IDI guide specifically designed for this purpose.

**Results**

Level of partograph use was92.4%. Blank and inadequately filled partographs were 6.2 and 1.4%, respectively, with a statistical significance between good birth outcomes and partograph use. Availability of guidelines, being knowledgeable about partograph, perceived as not a time-consuming task, received maternal and newborn care training, favorable maternal and fetal outcomes, presence of facility management support, regular mentorships and supportive supervision enabled the use partograph. Shortage of OCPs and consequent workload, different levels of competencies among HCPs, unavailability of partograph, lack of equipment and medical supplies, not easy to use, poor attitude towards partograph, lack of motivation, negligence, laziness, congested and non-spacious labour wards hindered partograph use. Other factors included working experience and OCPs profession.

**Conclusion**

It remains Confirming the strategy of the Ministry of Health, the introduction of a neonatal care unit with all necessary trainings and equipment can result in a significant increase in survival rate of new-born over time unclear at which cost and efficiency, the intervention can be scaled up to national level.

## A149. Awareness of risk factors, complications, and care of type 2 diabetes mellitus among adults aged above 18 years living at Boko in Dar Es Salaam

### Kahungo S Mkumbwa^1^

#### ^1^ St. Joseph College of Health and Allied Sciences, Dar es salaam, Tanzania

##### **Correspondence:** Kahungo S Mkumbwa (kahungosalehe7@gmail.com)

*BMC Proceedings 2023,* **17(13):** A149

**Introduction**

Diabetes mellitus (DM) is classified based on the pathogenic procedure characterized by persistent hyperglycemia. DM is the main cause of renal failure, non-traumatic lower-extremity amputations, and visual impairment. It also predisposes to cardiovascular diseases. The worldwide predominance of DM has risen dramatically in recent decades.

**Methodology**

Analysis of questionnaire of 100 adult participants was done by using EPI info version 7. Ethical clearance and consent forms were obtained from the concerned parties, while privacy and confidentiality were being strictly observed during all times of the study.

**Results**

The study revealed poor knowledge on awareness of risk factors of DM type 2 only 16.67% of the respondent knew risk factors of diabetes mellitus type 2, as well as inadequate knowledge about complications of DM type 2 among participants only 16.67% was aware of it as well and the knowledge on awareness of DM type 2 care was 34.11% which indicates an extensively poor understanding of DM type 2 in general among the participants.

**Conclusion**

This study was done on a few representative samples of Boko residents in Dar es Salaam which reflects the poor knowledge and awareness about diabetes in Dar es Salaam, Tanzania. This emphasizes that there is still a need for education on diabetes mellitus.

## A150. Engagement of laboratory stakeholders in reducing stock out of hvl/eid commodities in Tanzania

### Moses Madeje^1^, Yefta Emmanuel^1^, Christopher Henjewele^1^, Fredrick Mrema^1^, Redempta Mbatia^1^, Benedicta Masanj^1^, Lilian Shija^2^, Peter Mkama^3^

#### ^1^Tanzania Health Promotion Support (THPS), Dar es salaam, Tanzania; ^2^Centre for Disease prevention and Control (CDC), Dar es salaam, Tanzania; ^3^National Health Public Laboratory (NHPL), Dar es salaam, Tanzania

##### **Correspondence:** Moses Madeje (mmadeje@thps.or.tz)

*BMC Proceedings 2023,* **17(13):** A150

**Background**

Recurrent stock outs of HVL/EID testing reagents in Tanzania have been due to suboptimal quantification; low adherence of order entry to supplier, supply plans and incomplete set shipment. This interrupted HVL/EID sample testing resulting into high sample backlog and delayed results leading to suboptimal client management.

THPS through CDC advocated for full engagement of implementing partners (IPs) in provision of technical assistance on national HIV commodities quantification and forecast meetings.

**Methodology**

An analysis of HVL/EID commodities months of stocks (MoS) from September 2021 – April 2022 to understand trends of availability in the country was done. Also, THPS and other IPs participated in the ‘National Quantification’ activities which adjusted pipeline data and shipment supply plans. A distribution plan was developed by stakeholders based on stock on hand, test volume and sample backlog to avoid incomplete set delivery. Joint MOH-THPS supportive supervision and On-job-training to MSD staff and HVL/EID testing labs were done.

**Results**

Most of HVL/EID commodities for all platforms available in Tanzania, i.e. Abbott, CAP/CTM, COBAS4800, COBAS6800/8800, and GeneXpert were received at MSD in December

**Conclusion**

Full involvement of IPs in the national health commodities quantification activities, data analysis and assumptions as Technical Assistance increases quality of logistic data, reduces logistics data quality discrepancies, improves supply plan, prevents stock outs and improve health commodities availability hence uninterrupted HVL/EID testing countrywide.

## A151. The impact of Zanzibar’s national, government-owned, digital community health program on maternal and child healthcare in Zanzibar

### Mariam Mapande^1^, Tracey Li^1^, Mohammad Abdalla Rashid^1^

#### ^1^D-tree International, Dar es salaam, Tanzania

##### **Correspondence:** Mariam Mapande (mmapande@d-tree.org)

*BMC Proceedings 2023,* **17(13):** A151

**Case Report**

Community health requires individuals to take responsibility for their own health. Instead of relying solely on health facilities, individuals are encouraged to lead a generally healthy lifestyle to improve their own health and prevent the spread of infectious diseases. CHVs have the ability to promote these positive healthy behaviors whilst observing the local culture and needs of their communities. In view of this, the Ministry of Health updated the national community health strategy which led to the roll out of the “Jamii ni Afya” program. The roll out involved sensitization meetings with different district and community stakeholders, and the recruitment and training of over 2000 CHVs who are now delivering essential reproductive, maternal and child health services to their communities. Prior to the roll out of the community health program, many Zanzibaris relied on the use of traditional medicines during pregnancy and to treat childhood illnesses, and did not understand the importance of seeking treatment from health facilities. However, since CHVs started delivering services to promote birth delivery at facilities, immunization for children, and issuing referrals to pregnant women and children with danger signs, over 85 percent of pregnancy clients enrolled in the program now deliver at a facility compared to the baseline of 66 percent, and over 95 percent of clients that are issued with a referral complete the referral. These results demonstrate that CHVs are very effective at instigating positive behavior changes within their communities, which will lead to improved health outcomes amongst the population.

**Background**

In 2019, the Zanzibar Ministry of Health, supported by D-tree International, launched the Jamii ni Afya (“Communities are Health”) program. This is a national-scale community health program that utilizes technology to improve health service delivery. It is the first and only community health program in Zanzibar to be led and managed by the government. The program aims to bring high quality health services to every household in Zanzibar by equipping Community Health Volunteers (CHVs) with digital tools that enable them to deliver personalized health promotion, counselling, and referral services to their communities.

**Conclusion**

The national community health program in Zanzibar demonstrates how CHVs can play a major role in improving population health outcomes. Whilst there have been many previous community health programs in Zanzibar, the Jamii ni Afya program is the only one to have reached national scale. Due to its success, the government of Zanzibar continues to lead the program’s implementation and ownership, and is actively working to ensure the long-term sustainability of the program.

## A152. Use of geospatial technologies to increase access to vmmc services in hard to reach population in Simiyu region, Tanzania

### Peter Tulizo^1^, Shangwe Kimati^1^, Roki Mugeniwalwo^1^, Rita Mutayoba^1^, Christiana Mmasa^2^, Upendo Msanjila^2^

#### ^1^Amref Health Africa – Tanzania, Dar es salaam, Tanzania; ^2^University of Maryland Baltimore (UMB), Maryland, USA

##### **Correspondence:** Peter Tulizo (Peter.Sewa@Amref.org)

*BMC Proceedings 2023,* **17(13):** A152

**Background**

Voluntary medical male circumcision (VMMC) reduces the risk of heterosexual transmission of HIV by 60%. Apart from HIV, VMMC also provides protection against other sexually transmitted infections (STIs) to men and cervical cancer to female partners, and prevention of penile cancer in men. (Nzamwita and Biracyaza 2021b).

Simiyu region being one of the regions with low male circumcision prevalence (30%) VMMC programs were started in the last nine years, and ever since about 251,737 men have been reached by the VMMC services (DHIS2).

With time, interventions to reach men for circumcision became difficult. With the ambitious target of reaching 51,228 males aged 15+ with VMMC services in FY2022/2023, Amref Health Africa Tanzania through CDC support deployed geospatial technologies i.e. GIS mapping of hotspot areas to identify potential area of uncircumcised men aged years 15+.

**Methodology**

We collected data from hotspots (areas where most men congregate including mining, fishing, auction and market spots) as catchment areas for VMMC static facilities and satellite sites that helped to strategically plan the location of VMMC satellite services with potential of having more uncircumcised men. We gathered data on variables such as; hotspot's geographic location (coordinates), men major economic activities, infrastructure availability of VMMC services in nearby health facilities, and the catchment population. The collected data were overlaid with other demographic, program performance, and service delivery data to identify the VMMC gaps and needs in Simiyu region.

**Results**

Information on GIS mapping, showed fishing and mining activities are major economic activities that gather more men hence a potential of having uncircumcised men, followed by auction/open markets. Maps showed also wards with a high number of males aged 15+ are located in fishing communities in hard to reach wards , and multiple hotshpot are located in communities that are more than 25 km from static sites. The program used results from this exercise to establish new static sites and satellelite sites; as a results performance of Simiyu region increased by 37% from 8,108 males aged 15 years and above from October-December(Q1)FY2021 to 21,762 males above the same age by March(Q2)FY2022.

**Conclusion**

The utilization of geospatial technology provides a new roadmap to facilitate program in allocation of resources where are highly needed.

## A153. Data management best practice during covid 19 vaccination campaign and clearing backlog in Geita region

### Homobonius R. Longino^1^, Mashavu Yussuf^1^, Adam Amir^1^, Mary Mwanyika Sando^1^, Frank Mapendo^1^, Eva Ngwayemba^1^, Immakulata Uchad^1^, Marygoreth John^1^, Hamis Karisha^1^

#### ^1^Africa Academy for Public Health, Dar es salaam, Tanzania

##### **Correspondence:** Homobonius R. Longino (hlongino@aaph.or.tz)

*BMC Proceedings 2023,* **17(13):** A153

**Background**

Data management for COVID 19 Vaccination has been challenge in general population mostly during Surge activity campaign leading to data inconsistence. Geita Region formulated a best practice of data management during the Surge campaign for general population and ensuring data entered in Chanjo COVID system. This practice will enable other region to improve data collection and managing backlog and ensure quality of data during campaign in the general population.

**Methodology**

This exercise aimed to assess the quality of data of COVID 19 Vax surge which was conducted within seven days. The activity involved 164 Health care workers for data entry of 257,188 Doses. Tools were developed to monitor entry and quality of data reported and was piloted in 22 facilities during surge campaign. A cohort was established on vaccination data for easy monitoring of backlog and Document Review was done to ensure completeness of documentation in Register.

**Results**

Maximization (Surge campaign) produced 257,188 clients vaccinated within seven days. Documentation of patient information’s was complete in ChanjoCovid National Register. On system Assessment data produced met all criterial for use in decision making where Consistence of data between Register, Daily Reports, community daily monitoring, IVD DHIS2 were 100% Except CovidChanjo entry was done by 40%. Tool for monitoring entry was employed to ensure all data are being entered in the system which covered one month as shown on the graph below.

**Conclusion**

Backlog management for COVID 19 data in the maximization campaigns need to apply the above methodology through strengthening reporting and monitoring of data on daily basis by establishing cohorts of all vaccination done.

## A154. Prevalence and factors associated with under and over nutrition among in-school adolescents in urban Tanzania

### Mashavu Yussuf^1^, Dominic Mosha^1^, Sachin Shinde^2^, Mary Mwanyika Sando^1^, Wafaie Fawzi^2^, Isaac Lyatuu^1^, Frank Mapendo^1^, David Sando^3^, Amani Tinkasimile^1^

#### ^1^Africa Academy for Public Health, Dar es salaam, Tanzania; ^2^Harvard T. H Chan School of Public Health, Massachusetts, USA; ^3^Management and Development for Health, Dar es salaam, Tanzania

##### **Correspondence:** Mashavu Yussuf (myussuf@aaph.or.tz)

*BMC Proceedings 2023,* **17(13):** A154

**Introduction**

Malnutrition is among major public health problems globally with the burden being higher in Asian and Sub-Saharan African countries including Tanzania. In rural Tanzania a study reported that 18% of adolescents were stunted, 14% had thinness and 5.2% adolescents were overweight/obese. There is scarce understanding of malnutrition among adolescents aged 10-14 in urban Tanzania. We aimed to investigate prevalence and determine factors associated with stunting, underweight and overweight among adolescents in urban Tanzania.

**Methodology**

A cross-sectional study analysed data of 1,219 in-school adolescents aged 10 – 14 years in Dar es Salaam, Tanzania. Multivariable logistic regression determined factors associated with stunting, underweight and overweight. Analyses accounted for school-level clustering and associations were deemed statistically significant at p value < 0.05 during adjusted analyses.

**Results**

Overall, 11.6% of adolescents were stunted, 7.8% were underweight, 8.7% were overweight and 4.3% obese respectively. Average PDQS score was 18.1 (SD 3.2). Age, gender and wealth quintiles were significant factors associated with stunting. Age, gender and number of siblings in the household were significant factors associated with underweight. Gender, mother’s occupation and wealth quintiles were significant factors associated with overweight. Female adolescents were less likely to be stunted [AOR 0.68; 95%CI 0.47 – 0.98], less likely to be underweight [AOR 0.64; 95%CI 0.43 – 0.95] but more likely to be overweight compared to males [AOR 1.66; 95%CI 1.10 –2.51].

**Conclusion**

Tanzania is currently facing double burden of under and overnutrition. Significant drivers of malnutrition include age, gender, socio-economic status and number of siblings in the household. There is an urgent need to revise health policies and interventions to curb this double burden of malnutrition and prevent associated diseases in adulthood.

## A155. Strengthening health information data use and data quality in Tanzania mainland through the introduction of district of excellence

### Mustafa Ngwila^1^, Wilfred Senyoni^1^, Frida Mndeme^1^

#### ^1^HISP Tanzania, Mazinde Street, Dar es Salaam, Tanzania

##### **Correspondence:** Mustafa Ngwila (ngwilamustafa56@gmail.com)

*BMC Proceedings 2023,* **17(13):** A155

**Introduction**

The Health Information System Program (HISP) Tanzania has over a number of years been technically supporting the Ministry of Health (MoH) in managing routine health data management through implementation of District Health Information Software version 2 (DHIS 2). After the national rollout of DHIS2 in 2014, a lack of avenues for testing different approaches has emerged, as all sites are routinely using DHIS2. There was, therefore, a need to have a place where different.

**Methodology**

In order to achieve DoE objectives, four thematic areas were designed to approach the initiative The areas include Data Management and Information Use that shall strive to enhance data management and the use of data for decision-making in the health sector at different levels. Capacity Development involves well-planned sessions to orient managers at the councils and the health service providers at the health facility levels before any training session. Training on Digital Solutions, Tools and Procedures are the major two sets of capacity-building training sessions that will be provided. Research and Documentation on examining the existing tools and approaches, proposing new appropriate ones, documenting best practices, and others. Also, Documentation and Knowledge Dissemination translating research findings into practical details helpful for the DoE objectives. Digital Innovations, the DoE is also ground for testing and generating best practices for the implementation of digital innovations developed by the HISP community.

**Results**

The expected outcomes and impact of the DoE on Strengthening health information systems in Tanzania includes; Institutionalization of data management and use processes at district and facility levels. Also, wider dissemination of health information and organizational performance to key stakeholders and the general public. Self-reliance in program monitoring and planning at the district and facility level.

**Conclusion**

This project is expected to provide a place where different approaches and interventions will be crafted, addressed and tested. In the long run, the DoE will strengthen Tanzania Health Information Systems (HISs) by improving the use of Health Information data for decision-making at all levels.

## A156. Harnessing digital technologies to improve adolescent health in Tanga, Tanzania.

### Imani Irema^1^, Mary Mwanyika Sando^1^, Julieth Bitabo^5^, David Sando^2^, Abbas Ismail^3^, Agnes Mchome^6^, Charles Mkombe^6^, Mashavu Yussuf^1^, Wafaie Fawzi^4^

#### ^1^Africa Academy for Public Health, Dar es salaam, Tanzania; ^2^Management and Development for Health (MDH), Dar es salaam, Tanzania; ^3^University of Dodoma, Dodoma, Tanzania; ^4^Harvard T.H.Chan School of Public Health, Massachusetts, USA; ^5^Tanzania Food and Nutrition Centre, Dar es salaam, Tanzania; ^6^Tanga City Council, Tanga, Tanzania

##### **Correspondence:** Imani Irema (iirema@aaph.or.tz)

*BMC Proceedings 2023,* **17(13):** A156

**Introduction**

Tracking longitudinal data on adolescent health risks, behaviors and outcomes is critical to inform interventions and policies; however, these data are lacking in low- and middle-income countries (LMICs). In Tanzania, an adolescent health study was conducted on January 2022 in Tanga region among 1262 adolescents (10 to 19). The study aimed at addressing this knowledge gap, gaining an understanding and tracking of adolescent health and nutrition through standardized survey instrument.

**Methodology**

Adolescents receive monthly messages sent using SMS BOT platform. The platform is installed in android device and includes 3 applications: (1) A trigger is used to create and broadcast the messages to the adolescents. Messages are automatically personalized to include participants names. (2) A listener receives the messages, process them using predefined algorithms and sends the correct replies to the adolescents. (3) An exporter is used to export the messages from the android device in csv format for further analysis.

**Results**

Out of 1262 participants that were engaged, 910 agreed to receive the monthly messages. 791 of the agreed individuals received the messages in the first month (March) and 112 responded that the message was helpful. Having showed promising results in the first month, the SMS BOT platform is a capable means of getting in touch with the adolescents through the personalized messages which will emphasize the importance of nutrition and dietary diversity, healthy eating, and lifestyle practices.

**Conclusion**

Digital approaches are promising interventions in reaching the important age group of adolescents with personalized and tailored health and nutrition interventions to prevent diseases in adulthood.

## A157. Use of DREAMS ambassadors in improving uptake of hiv pre-exposure prophylaxis (prep) among agyw aged 15-24 in 17 facilities of Shinyanga region

### Agnes R. Kirato^1^, Jane Kashumba^1^, Jane Kitalile^2^, Jaston Sanga^1^, Yudas Ndungile^3^, Amos Scott^1^, Fredrick Ndossi^1^, Geofrey Mabu^4^

#### ^1^Tanzania Health Promotion Support ( THPS), Dar es salaam, Tanzania; ^2^Centre for Disease Control ( CDC Tanzania), Dar es salaam, Tanzania; ^3^Shinyanga Regional Medical Officer, Shinyanga, Tanzania; ^4^Shinyanga TACAIDS Regional coordinator, Shinyanga, Tanzania

##### **Correspondence:** Agnes R. Kirato (akirato@thps.or.tz)

*BMC Proceedings 2023*, **17(13):** A157

**Background**

Use of AGYW Ambassadors, coupled withpeer-to-peerr support led to improved PrEP uptake among vAGYW and provided a platform to address social stigma as potential barriers for PrEP uptake. AGYW prefer confidential services and discrete packaging that do not identify them as PrEP users.

**Methodology**

Since October 2021, THPS trained a pool of 32 AGYW as DREAMS ambassadors from working at 17 facilities in Shinyanga region to provide patient-centered services in vAGYW in a friendly, efficient, and respectful environment, and created a supportive environment for provision of PreP. Ambassadors collaborate with healthcare workers to conduct screening using the standardized eligibility screening tool coupled with peer to peer counselling and support, providing first hand PrEP sensitization on the benefits of PrEP for AGYW. They also address medication associated stigma thus increasing uptake of PrEP, AGYW Ambassadors also facilitate quarterly Clubs; these being forums where AGYW meet and discuss their well-being and barriers to PrEP uptake.

**Results**

There was a 16-fold increase in uptake of PrEP for new clients among vAGYW across DREAMS supported facilities from 44 in October 2021 to 704 as of June 2022; surpassing Shinyanga region annual PrEP target among vAGYW by 159% (704/443)

**Conclusion**

The Determined, Resilient, AIDS-Free, Mentored and Safe Women (DREAMS) Initiative plays a key role in HIV prevention through promotion of pre-exposure prophylaxis (PrEP) with anti-retroviral drugs for at risk adolescent girls and very young women (vAGYW) aged 15-24 in Tanzania. Hover, the uptake of PrEP among vAGYW in Shinyanga region had remained slow.

## A158. Role of healthcare workers' context in providing cervical cancer screening to women living with hiv in Kinondoni, Dar Es Salaam

### Notikela M. Nyamle^1^, Emmy Metta^1^, Dunstan R Bishanga^1^

#### ^1^Muhimbili University of Health and Allied Sciences, Dar es salaam, Tanzania

##### **Correspondence:** Notikela M. Nyamle (nyamlenotikela@gmail.com)

*BMC Proceedings 2023,* **17(13):** A158

**Background**

Globally an estimate of 604,000 new cases and 342,000 deaths in 2020 were attributed to cervical cancer. Among East African countries, Tanzania has the highest burden of cervical cancer. Cervical cancer screening coverage for Women Living with HIV/AIDs in Tanzania ranges from 3% to 46% which is below the WHO target coverage of 75% by the year 2030. Workplace context plays an important role in providing cervical cancer screening by altering health care workers’ motivation, engagement, positive patient interaction, and confidence to initiate dialogue for cervical cancer screening among women living with HIV.

**Methodology**

A case study approach was used for a depth description of the role of healthcare workers’ context in providing cervical cancer screening to women living with HIV. A total of twelve healthcare workers were recruited for in-depth interviews done in three HIV Care and Treatment Centres (CTC) of government-based primary health care facilities in Kinondoni, Dar es Salaam. The inductive content analysis process was followed in analysing the data.

**Results**

Findings of this study revealed that; training enhanced healthcare workers’ self-efficacy in cervical cancer screening by building competencies for cervical cancer screening and enhancing professional development; guidelines utilization exposed healthcare workers to evidence-based practices and assured consistency of screening services. Healthcare workers’ workload influenced task distribution, while performance feedback informed cervical cancer screening improvement progress and motivated the performance of healthcare workers through job satisfaction. However, they were challenged by a lack of refresher training courses and limited access to guidelines.

**Conclusion**

Health care workers acknowledge the importance of training, guidelines, workload, and performance feedback. However, they are challenged by a lack of refresher courses and limited access to guidelines. Refresher training courses and cervical cancer screening guidelines orientation strategy are recommended.

## A159. Prevalence and factors associated with covid-19 vaccine uptake among geriatric population in Kilimanjaro region, Tanzania

### Kelvin M. Musa^1^, Shadrack lwila^1^, Pulkeria Shayo^1^, Innocent B Mboya^1^, FaustineChinilo Kimondo^1^, Christer Kyando^1^

#### ^1^Kilimanjaro Christian Medical University College, Moshi, Tanzania

##### **Correspondence:** Kelvin M. Musa (kelvinmusa67@gmail.com)

*BMC Proceedings 2023,* **17(13):** A159

**Introduction**

COVID-19 is a public health emergency and an international concern. Older adults are at higher risk of developing a severe form of COVID-19. COVID-19 vaccines are considered effective in lowering the risk of getting serious illness and death in older adults. Understanding different factors associated with COVID-19 vaccination is crucial in promoting vaccination uptake in this population.

**Methodology**

Community-based cross-sectional study was conducted in Moshi rural and urban districts of Kilimanjaro region among 391 older adults (60+ years). A multistage random sampling technique was used to select study participants and interviewed using a questionnaire. All statistical analyses were performed using SPSS version 20.0. Multivariable logistic regression, estimated adjusted odds ratio (OR) and 95% confidence intervals (CI) for factors associated with vaccination uptake. A p-value<0.05 declared a statistically significant association.

**Results**

Among 391 older adults (60+ years), the mean (sd) age was 68.4 (8.0), 198 were female and 224 (57.3%) received any of the available COVID-19 vaccines at least once. The majority (82.1%) of those vaccinated felt the vaccine was safe and effective. Higher odds of COVID-19 vaccine uptake was among males [AOR = 1.69, 95% CI = 1.13-2.529 p=0.011 ], those living in urban [AOR = 2.00, 95% CI = 1.33-3.00 p=0.001], and those who trust in the government [AOR = 3.06, 95% CI: 1.45–6.48, p=0.003].

**Conclusion**

Nearly six in every ten of older adults in Kilimanjaro region have received at least one vaccination dose. Efforts to increase vaccine uptake should target females, rural residents and promoting awareness on safe and effectiveness of COVID-19 vaccine.

## A160. Magnitude and predictors of high 10- year cardiovascular risk among diabetic patients – a single center study in Tanzania

### Nadeem Kassam^1^, Robert Mayenga^1^, Iris Matei^1^, Exuper Chuwa^1^, Gijsberta Jengo^1^, Eric Aghan^2,^ Harrison Chuwa^3^, Kamran Hameed^3^

#### ^1^Department of Internal Medicine, Aga Khan hospital, Mwanza, Tanzania; ^2^Department of Family Medicine Aga Khan University Medical College East Africa; ^3^Department of Internal Medicine, Aga Khan Hospital, Dar es Salaam, Tanzania

##### **Correspondence:** Nadeem Kassam

*BMC Proceedings 2023,* **17(13):** A160

**Background**

Diabetes mellitus is a major risk factor for Atherosclerotic Cardiovascular Disease (ASCVD) and premature mortality. Tanzania is experiencing a higher burden of non-Communicable disease (NCDs) with ASCVD being the most prevalent especially among adults aged 40 years and above. Modifiable risks are variable amongst different population and central to the development of ASCVD. This study aimed to identify magnitude of ASCVD among a diabetic cohort and its associated risk enhancing factors amongst a cohort of patients in Tanzania.

**Methods**

An observational prospective study conducted at the Aga Khan hospital, Mwanza. Individuals 10 – year risk was calculated based on the ASCVD risk calculator developed by American Health Association (AHA) and American college of cardiology (ACC) in 2013. Multivariable analysis was applied to determine risk enhancing factors for individuals who had a high 10 year risk of ASCVD.

**Results**:

The study recruited a total of 99 participants over a period of 3 months. The median age of the study population was 60 years [IQR: 52-68] years, most of whom were of African origin (n=89, 89.9%), having a BMI of >30kg/m^2^ (n=57, 57.6%) and suffering with concomitant Hypertension (n=64, 64.6%). Majority of our cohort had a high 10 -year risk of suffering ASCVD (n=59, 59.6%) The studyidentified duration of Diabetes Mellitus (>10 years) (OR 8.15, 95% CI 2.47–31.20) and increased creatinine (OR 1.03, 95% CI 1.01–1.05) to be risk enhancing factors associated with high 10-year risk of ASCVD amongst our diabetic cohort.

**Conclusion**

This study demonstrates the predictive burden of ASCVD amongst diabetics and validates the great need of comprehensive patient centered approach for primary prevention of ASCVD which includes optimal glycemic and blood pressure control and the need of statin as cholesterol lowering agent.

## A161. Perception, attitude and hesitancy as factors associated with uptake of covid-19 vaccines among adults across Tanzania june, 2022

### Pilly H. Ndobeji^1^, Deusdedith Boniphace Bulimbe^1^, Devis Grant Mafuru^1^, Raymond Mchande^1^, Ferdinand Allute^1^, John Alfred^1^, Mahsin Rashid^1^, Secilia Kapalata^, 2^, Deogratius Bintabara^1, 2^

#### ^**1**^School of Medicine and Dentistry, the University of Dodoma, Dodoma, Tanzania; ^**2**^Department of Community Medicine, the University of Dodom, Dodoma, Tanzania

##### **Correspondence:** Pilly H. Ndobeji (ndobejipilly@gmail.com)

*BMC Proceedings 2023,* **17(13):** A161

**Background**

Herd immunity at population level in Tanzania is promising campaign against COVID-19 which has bottlenecks, among of them are negative attitude, poor perception and hesitancy. This study surveys the views of adults across Tanzania by looking into attitude, perception and hesitancy towards COVID-19 vaccine uptake and determining their Vaccination Status.

**Methods**

A cross-sectional study design was conducted from June to July 2022. A multistage sampling used to recruit total of 22,844 participants aged 18 years and more. Conveniently selecting regions Arusha, Iringa, Morogoro and Mwanza. Pretested Online questionnaire was used and SPSS and Pearson chi-square were used for analysis of obtained data and their association respectively.

**Results**

In this study, 61.2% and 66.7% of all participants scored positive attitude and good perception towards COVID-19 vaccines respectively. 63.3% agreed to presence of several side effects from the vaccines and 61.2% and 59.0% agreed that vaccines are safe and effective respectively. 71.2% of participants showed Hesitancy to get vaccinated or encourage family/friend to get vaccinated or both, from which Government employees and adults living with chronic diseases had the least hesitancy levels of 63.6% and 68.3% respectively while Unemployed and adults aging from 18-29 years had higher levels of Hesitancy of 73.3% and 71.5% respectively. The study population showed overall poor uptake of COVID-19 vaccines of 20.1%.

**Conclusion**

We report that more than half of the Tanzanian adults have positive attitudes and perceptions towards the COVID-19 vaccine, with majority of them being hesitant to get vaccinated which explains to overall poor uptake of the vaccines. To increase COVID-19 vaccines uptake strategic interventional efforts should focus towards clearing people’s hesitancy following misconceptions on various concepts of COVID-19vaccines at Community level with chief involvement of Community Health Workers.

